# Tree community resource economics control soil food web multifunctionality

**DOI:** 10.1038/s41586-026-10455-1

**Published:** 2026-05-06

**Authors:** Ludovic Henneron, David A. Wardle, Matty P. Berg, Stephan Hättenschwiler, Jürgen Bauhus, François Buscot, Sylvain Coq, Thibaud Decaëns, Nathalie Fromin, Pierre Ganault, Lauren M. Gillespie, Kezia Goldmann, Radim Matula, Alexandru Milcu, Bart Muys, Johanne Nahmani, Luis Daniel Prada-Salcedo, Michael Scherer-Lorenzen, Kris Verheyen, Janna Wambsganss, Paul Kardol

**Affiliations:** 1 Department of Forest Ecology and Management, Swedish University of Agricultural Sciences, Umeå, Sweden; 2 ECODIV USC 1499, Université de Rouen Normandie, INRAE, Rouen, France; 3 Department of Ecology, Environment and Geoscience, Umeå University, Umeå, Sweden; 4 Amsterdam Institute for Life and Environment, Section Ecology and Evolution, Vrije Universiteit, Amsterdam, the Netherlands; 5 GELIFES, Community Conservation Group, Groningen University, Groningen, the Netherlands; 6 CEFE, Université de Montpellier, CNRS, EPHE, IRD, Montpellier, France; 7 Chair of Silviculture, Faculty of Environment and Natural Resources, University of Freiburg, Freiburg, Germany; 8 Future Forests Cluster of Excellence, University of Freiburg, Freiburg, Germany; 9 Department of Soil Ecology, UFZ - Helmholtz Centre for Environmental Research, Halle (Saale), Germany; 10 German Centre for Integrative Biodiversity Research (iDiv) Halle-Jena-Leipzig, Leipzig, Germany; 11 Department of Forest Ecology, Faculty of Forestry and Wood Sciences, Czech University of Life Sciences Prague, Prague, Czech Republic; 12 Montpellier European Ecotron, Université de Montpellier, CNRS, Montferrier-sur-Lez, France; 13 Department of Earth & Environmental Sciences, KU Leuven, Leuven, Belgium; 14 Geobotany, Faculty of Biology, University of Freiburg, Freiburg, Germany; 15 Forest & Nature Lab, Department of Environment, Ghent University, Melle-Gontrode, Belgium; 16 Research Institute for Forest Ecology and Forestry (FAWF), Landesforsten Rheinland-Pfalz, Trippstadt, Germany; 17 Department of Forest Mycology and Plant Pathology, Swedish University of Agricultural Sciences, Uppsala, Sweden

**Keywords:** Ecological networks, Food webs, Ecosystem ecology, Forest ecology

## Abstract

Plants affect terrestrial ecosystem functioning by shaping microenvironments^1^ and by providing the primary production that fuels energy flow into food webs^2^. However, how plant community properties affect ecosystem functioning via energy fluxes in food webs has been little studied^3,4^, especially for the soil food webs that channel most plant-derived energy^2,5^. Applying a food web energetics approach^6,7^, we show that the resource economics of dominant tree species control soil food web multifunctionality across European forests. Tree communities dominated by resource-acquisitive species promoted faster rates of multiple soil trophic functions than did communities dominated by resource-conservative species. These effects were primarily driven by higher-quality litter and warmer forest microclimates, leading to increased metabolic activity of soil organisms^8^. Accordingly, tree species composition explained a large portion of variation in soil food web multifunctionality, comparable to that explained by biogeographic differences among locations. By contrast, mixtures of three tree species had weakly negative effects relative to single-species stands, mostly due to shifts in energy channelling from living fine roots to litter and a cooling effect on forest microclimate. This occurred despite an overyielding effect in aboveground tree biomass production, suggesting contrasting diversity effects above- and belowground. Our findings emphasize the importance of plant functional traits related to resource economics as drivers of soil food web functioning^5,9^ and demonstrate how climate-driven shifts in tree community composition may alter forest soil functioning.

## Abstract (web summary)

Plant community properties affect ecosystem functioning via energy fluxes in food webs, with the leaf and root resource economics of dominant tree species controlling soil food web multifunctionality.

## Main

Biological communities are experiencing major changes across trophic levels and habitats globally as a result of climate change, land transformation and species invasions^10^. Ecosystem processes such as productivity, decomposition and biogeochemical cycling are shaped by multiple community-level properties, including diversity and composition of species but also of trait spectra^11–13^. Yet, most research linking community properties to ecosystem functioning has focused on single trophic levels or simple food chains, despite growing recognition of the need for multitrophic perspectives to better understand these relationships and their relevance in real-world ecosystems^4,14^. In terrestrial ecosystems such as forests, grasslands and croplands, management often relies on the selection and mixing of plant species with particular trait values to enhance ecosystem functioning and the provisioning of associated services^15^. Plant diversity and community composition have frequently been shown to operate as major drivers of net primary productivity^3,16^, as well as of consumer communities across trophic levels, especially in aboveground producer-based (‘green’) food webs^16–18^. Nonetheless, the broader influence of plant community properties on terrestrial ecosystem functioning through cascading effects across trophic levels remains little explored, as few studies have explicitly applied a quantitative food web approach in this context^3,4^.

**Fig. 1:**
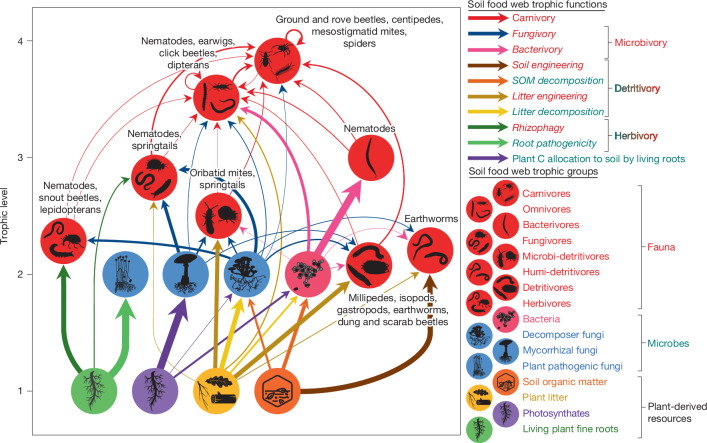
Illustration of the trophic functions and topography of the soil food web. Dominant taxonomic groups in faunal trophic groups are shown with text within the figure. Arrows indicate energy flows among trophic groups, with arrow widths corresponding to trophic interaction strengths. Arrow colours show how energy fluxes were aggregated by resource and consumer types to quantify trophic functions of the soil food web. These are related to five broad trophic functions: carnivory, the consumption of fauna; microbivory, the consumption of microbes (‘bacterivory’ and ‘fungivory’); plant C allocation to soil by living roots, the consumption of photosynthates and rhizodeposits from plant roots by mycorrhizal fungi and decomposer microbes; herbivory, the consumption of plant roots by microbes (‘root pathogenicity’) or fauna (‘rhizophagy’); and detritivory, the consumption of detritus (‘plant litter’ or ‘soil organic matter, SOM’) by microbes (‘decomposition’) or fauna (‘engineering’). Decomposition refers to the assimilation and mineralization of dead organic matter through respiration. Engineering refers to the physical modification, maintenance or creation of habitats by feeding activity (Methods). Italicized functions are subsets of broader functions (not italicized) between the dotted lines. Trophic functions in blue-green and dark red indicate those mediated by microbes and fauna, respectively. Trophic interaction strengths shown here are based on biomass data averaged across all 64 plots, but plot-specific biomass data were used to calculate trophic interaction strengths for each local food web. To simplify the representation, trophic guilds (finer level) were aggregated into trophic groups (coarser level) by averaging their trophic interaction strengths, weighted by the relative biomasses of the corresponding guilds within each group. See Supplementary Fig. 1 for a more detailed illustration of the food web topology and trophic interaction strengths among trophic guilds. Silhouettes were reproduced from the Noun Project (https://thenounproject.com) and PhyloPic (https://www.phylopic.org/). Plant roots, created by ohyeahicon under a CC BY 3.0 Universal Public Domain licence; leaf litter, created by Imogen Oh under a CC BY 3.0 Universal Public Domain licence; root litter, created by Amantaka under a CC BY 3.0 Universal Public Domain licence; wood litter, created by Hafid Firman Syarif under a CC BY 3.0 Universal Public Domain licence; soil organic matter, created by Foodicons Collection under a CC0 1.0 Universal Public Domain licence. Plant pathogenic fungi, created by Guillaume Dera under a CC0 1.0 Universal Public Domain licence; mycorrhizal fungi, created by Guillaume Dera under a CC0 1.0 Universal Public Domain licence; decomposer fungi, created by Guillaume Dera under a CC0 1.0 Universal Public Domain licence; bacteria, created by Matthew Crook under a CC BY-SA 3.0 Universal Public Domain licence; herbivore—nematodes, created by Kamil S. Jaron under a CC0 1.0 Universal Public Domain licence; herbivore—snout beetles, created by Kanako Bessho-Uehara under a CC0 1.0 Universal Public Domain licence; herbivore—lepidopterans, created by T. Michael Keesey under a CC PDM 1.0 Universal Public Domain licence; detritivores—gastropods, created by Gareth Monger under a CC BY 3.0 Universal Public Domain licence; detritivores—millipedes, created by Gemma Martínez-Redondo under a CC0 1.0 Universal Public Domain licence; detritivores—isopods, created by Birgit Lang under a CC PDM 1.0 Universal Public Domain licence; humi-detritivores—earthworm left-hand sided, created by RS under a CC0 1.0 Universal Public Domain licence; humi-detritivores—earthworm right-hand sided, created by Guillaume Dera under a CC0 1.0 Universal Public Domain licence; microbi-detritivores—springtails, created by Kamil S. Jaron under a CC0 1.0 Universal Public Domain licence; microbi-detritivores—oribatid mites, created by Birgit Lang under a CC BY 3.0 Universal Public Domain licence; fungivores—nematodes, created by Arcadia Science under a CC0 1.0 Universal Public Domain licence; fungivores—springtails at the top, created by Kamil S. Jaron under a CC0 1.0 Universal Public Domain licence; fungivores—springtails at the bottom, created by Birgit Lang under a CC BY 3.0 Universal Public Domain licence; bacterivores—nematodes, created by Gareth Monger under a CC BY 3.0 Universal Public Domain licence; omnivores—nematodes, created by Arcadia Science under a CC0 1.0 Universal Public Domain licence; omnivores—earwigs, created by Darren J. Parker under a CC0 1.0 Universal Public Domain licence; omnivores, created by T. Michael Keesey under a CC PDM 1.0 Universal Public Domain licence; predators—ground beetles, created by Michael Day under a CC0 1.0 Universal Public Domain licence; predators—centipedes, created by Birgit Lang under a CC0 1.0 Universal Public Domain licence; predators—mesostigmatid mites, created by Kamil S. Jaron under a CC0 1.0 Universal Public Domain licence.

Trophic interactions play a major role in underpinning ecosystem functioning because heterotrophic organisms regulate multiple ecosystem processes primarily through their food consumption^6^. Accordingly, quantifying energy flow along trophic links in food webs^19,20^ has been proposed as a powerful tool to mechanistically understand the relationships between community properties and ecosystem functioning in complex multitrophic systems^4,6^. Energy fluxes in food webs reflect trophic functions such as herbivory, detritivory, microbivory and predation (Fig. 1), and therefore provide a common currency for assessing ecosystem functioning across trophic levels^6^. These trophic functions can be combined to measure food web multifunctionality, which represents the simultaneous performance of multiple trophic functions carried out by the food web^6,7^. However, the application of food web energetics for an integrated understanding of how plant community properties affect ecosystem functioning remains in its infancy^21–23^, particularly for belowground food webs that play a critical role in ecosystem functioning^5,7,24^.

In most terrestrial ecosystems, the majority of energy derived from primary production directly enters the soil as freshly dead organic matter, that is, plant litter^2^. Consequently, the dominant channel of energy flow is through the detritus-based (‘brown’) food web^5^, which underpins decomposition and nutrient cycling processes and ultimately controls the availability of growth-limiting nutrients to plants^25^. It also allows photosynthetically fixed carbon (C) to be stabilized in the soil or released back to the atmosphere through respiration, with major implications for ecosystem C cycling and sequestration^26^. Additionally, terrestrial ecosystems host an important belowground living root-based food web that involves root herbivory and plant C allocation to mycorrhizal fungi and rhizosphere microbes^5^, and which strongly influences the size and turnover of root biomass as well as the acquisition of belowground resources by plant roots^25^.

Plant community composition is well known to exert a strong control over ecosystem processes related to the soil food web^13^. This has traditionally been attributed to the functional traits of dominant plant species that are directly involved in the acquisition, processing and conservation of resources (that is, ‘economic traits’^9^), and which shape the quantity and nutritional quality of trophic resources such as litter^5^ and rhizodeposits^27^. However, empirical evidence that explicitly relates plant economic traits at the community level to the functioning of soil food webs is still lacking. Plant diversity is generally thought to promote soil processes by increasing the quantity and diversity of trophic resources and by fostering favourable micro-environmental conditions^28^. In line with this, two recent experiments have shown that plant species diversity can foster multitrophic energy flow across both above- and belowground food webs^22,23^. Nevertheless, how plant diversity and composition affect soil food web functioning in real-world ecosystems remains poorly understood and to date has not been explored simultaneously across multiple sites and climate zones.

**Extended Data Fig. 1:**
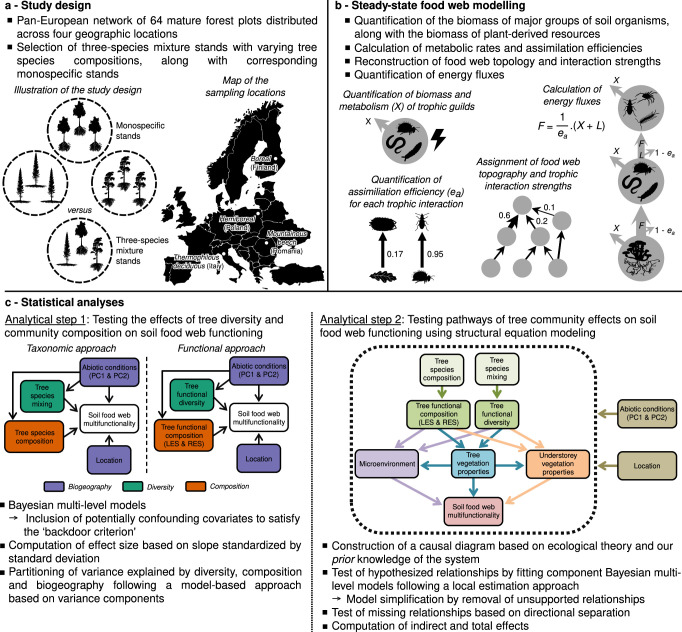
Overview of the study workflow, including sampling design (a), modelling of soil food web energy fluxes (b), and statistical approaches. (**c**). In panel c, the two left insets show model’s causal diagrams for each approach, while the right inset illustrates the structural equation metamodel. LES, leaf economics spectrum; RES, fine-root economics spectrum. Functional diversity was quantified as trait variability among tree species (functional dispersion). Functional composition was quantified as the community-weighted mean (CWM) of trait values. To reduce dimensionality, a principal component analysis (PCA) was applied to tree CWM traits, resulting in two major axes corresponding to the leaf and fine-root economics spectra (Extended Data Fig. 4a). Likewise, the first two PCA axes of abiotic conditions were used to represent potentially confounding environmental covariates (Supplementary Fig. 2a). The structural equation metamodel summarizes our a priori hypotheses on how tree community properties mediate soil food web multifunctionality. A full causal diagram of all variables and hypothesized relationships is provided in Supplementary Fig. 3. The basemap in **a** was reproduced from Publicdomainvectors (publicdomainvectors.org) under a CC0 1.0 Universal Public Domain licence. Silhouettes in **a** and **b** were reproduced from PhyloPic (https://www.phylopic.org/). *Pinus sylvestris*, created by Guillaume Dera under a CC0 1.0 Universal Public Domain licence; *Picea abies*, created by Guillaume Dera under a CC0 1.0 Universal Public Domain licence; *Betula pendula*, created by Guillaume Dera under a CC0 1.0 Universal Public Domain licence; decomposer fungi, created by Guillaume Dera under a CC0 1.0 Universal Public Domain licence; detritivores—isopods, created by Birgit Lang under a CC PDM 1.0 Universal Public Domain licence; fungivores—nematodes, created by Arcadia Science under a CC0 1.0 Universal Public Domain licence; fungivores—springtails at the top, created by Kamil S. Jaron under a CC0 1.0 Universal Public Domain licence; fungivores—springtails at the bottom, created by Birgit Lang under a CC BY 3.0 Universal Public Domain licence; predators—ground beetles, created by Michael Day under a CC0 1.0 Universal Public Domain licence; predators—centipedes, created by Birgit Lang under a CC0 1.0 Universal Public Domain licence; predators—mesostigmatid mites, created by Kamil S. Jaron under a CC0 1.0 Universal Public Domain licence. A silhouette in **b** was reproduced from the Noun Project (https://thenounproject.com). Leaf litter, created by Imogen Oh under a CC BY 3.0 Universal Public Domain licence.

Here, we investigated how different properties of naturally assembled tree communities, including their diversity (three-species versus single-species stands) and their composition in terms of taxonomic identity and trait values, influence multiple soil trophic functions across a variety of environmental contexts in European forests. We hypothesized that: (1) tree species mixing, through associated increases in functional diversity, positively affects soil food web multifunctionality; and (2) tree species composition exerts a strong effect on soil food web multifunctionality, owing to the key role of the resource economic traits of dominant tree species. We tested these hypotheses using a pan-European network of 64 mature forest plots distributed across four geographic locations in different countries (Finland, Poland, Romania and Italy), spanning boreal to Mediterranean climates and representing highly contrasting European forest types (Extended Data Fig. 1a). Within each location, we followed a stratified sampling design by selecting three-species mixture stands with varying tree species compositions, along with corresponding monospecific stands (Extended Data Fig. 1a, Extended Data Table 1 and Methods). This plot selection was performed so as to minimize covariation with potential confounding factors (Supplementary Fig. 2a), and thereby increase our ability to infer the effects of tree species mixing and composition on soil food web functioning.

To quantify energy fluxes in the belowground food web of each plot, we applied steady-state food web modelling based on ecosystem energetics^6^ (Extended Data Fig. 1b). This approach is grounded in the principle that, for a given trophic group within a food web, energy uptake through food consumption must balance energetic demands, including energy lost during food assimilation, and by metabolism (respiration^8^) and predation. We first quantified the biomasses, metabolic rates and assimilation efficiencies of major groups of soil organisms, including microbes and fauna, along with the biomass of plant-derived resources, that is, living fine roots and associated photosynthates, plant litter and soil organic matter (SOM). We then gathered soil organisms into trophic groups, established food web topology and quantified trophic interaction strengths to allow the calculation of energy fluxes^6,7^ (Fig. 1, Supplementary Fig. 1 and Methods).

**Fig. 2:**
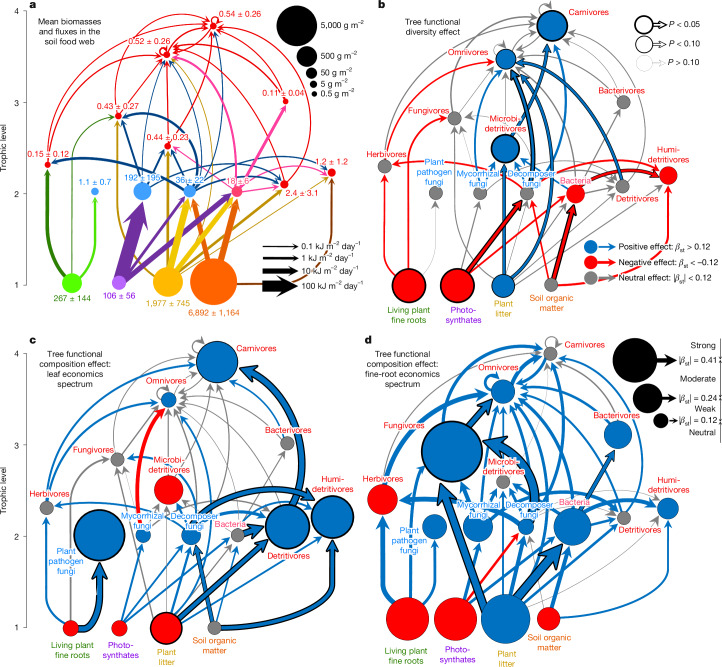
Effects of tree functional diversity and composition on soil food web functioning. **a**, Trophic group biomasses (circles, g dry weight m^−2^) and energy fluxes (arrows, kJ m^−2^ d^−1^) of the soil food web under average conditions. These are based on predicted values derived from a Bayesian linear mixed-effects model (equation 1; *n* = 64 plots; Methods) using mean values across all plots for each predictor. Circle areas and arrow widths are scaled by the cube root of posterior mean biomasses and energy fluxes, respectively. Text associated with circles shows the posterior mean and 95% credible intervals of trophic group biomass (posterior *n* = 10,000). Trophic group names are shown in other panels. The colours used for trophic functions and groups are the same as in Fig. 1. **b**–**d**, Effects of tree functional diversity (**b**) and composition (**c**, LES; **d**, RES) on trophic group biomasses and energy fluxes of the soil food web. Circle areas and arrow widths are scaled by the posterior mean of effect sizes, which are partial slope coefficients standardized by the standard deviation (*β*_st_), derived from Bayesian linear mixed-effects models (equation 2; *n* = 64 plots; Methods). Blue, red and grey circles and arrows indicate positive, negative and neutral effects, respectively (that is, *β*_st_ > +0.12, *β*_st_ < −0.12 and −0.12 ≤ *β*_st_ ≤ +0.12). The *P* values were derived from posterior distributions using a two-tailed test.

**Extended Data Fig. 2:**
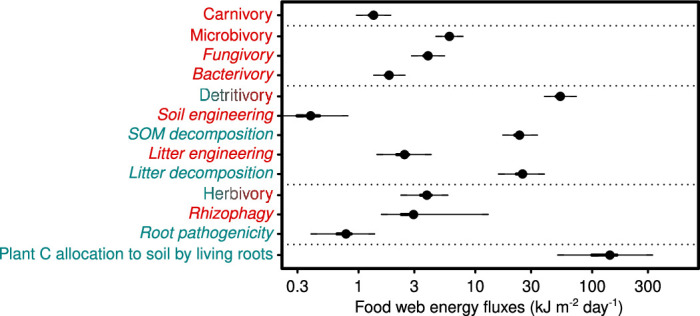
Soil food web trophic functions (aggregated energy fluxes per function) under average conditions. These are based on predicted values derived from a Bayesian linear mixed-effects model (equation 2; *n* = 64 plots; see Methods) using mean values across all plots for each predictor. Circles represent the posterior means, with thick and thin error bars representing 50% and 95% posterior credible intervals, respectively (posterior *n* = 10,000). The *x* axis is displayed on a logarithmic scale. Trophic functions are based on energy fluxes in the soil food web, aggregated by resource and consumer types (Fig. 1). Italicized functions are subsets of broader functions (not italicized) between the dotted lines. Trophic functions in blue-green and dark red indicate those mediated by microbes and fauna, respectively.

**Extended Data Fig. 3:**
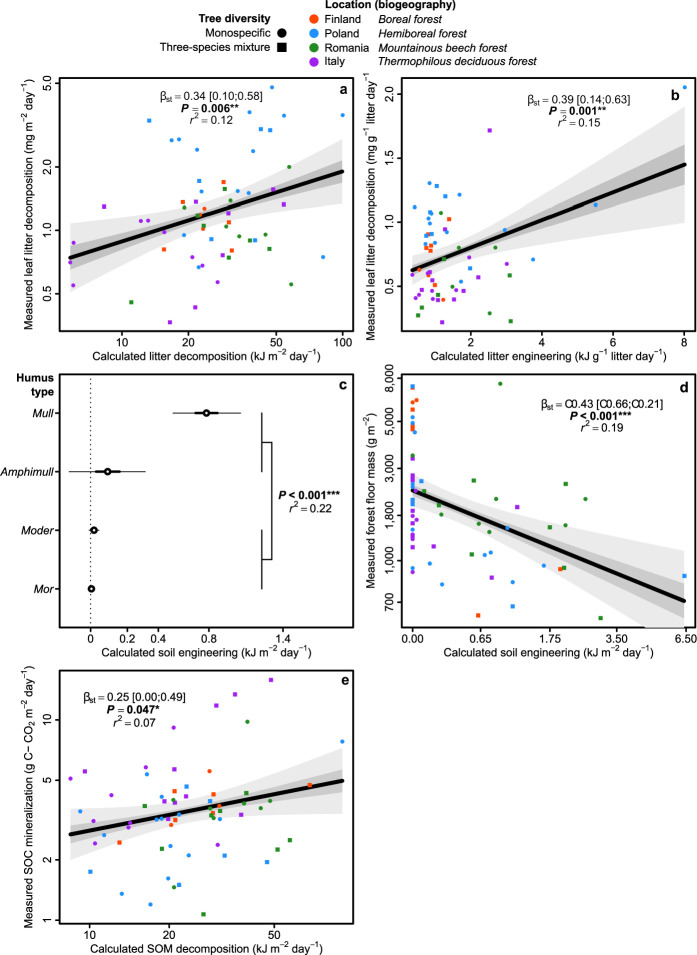
Relationships between calculated soil food web trophic functions and measured soil functions. Relationships were tested using Bayesian linear models (*n* = 64 plots). For all panels, calculated soil food web trophic functions and measured ecosystem processes or properties are displayed on the *x* and *y* axes, respectively. Thick dark lines and circles represent posterior means (posterior *n* = 10,000). Thick and thin error bars, along with dark and light grey filled area, represent 50% and 95% posterior credible intervals, respectively (posterior *n* = 10,000). β_st_ is the posterior mean of the slope coefficient standardized by the standard deviation (effect size). Values in square brackets are the 95% posterior credible intervals of the standardized slopes. For panels a, c, d, and e, the *x* and *y* axes are displayed on a logarithmic scale. The *p*-values were derived from posterior distributions using a two-tailed test. *, *p* < 0.050; **, *p* < 0.010; ***, *p* < 0.001.

Energy fluxes were then aggregated by resource and consumer types to quantify ten trophic functions of the soil food web^6,7^: plant C allocation to soil by living roots, root pathogenicity, rhizophagy, litter decomposition, litter engineering, SOM decomposition, soil engineering, bacterivory, fungivory and carnivory (Figs. 1, 2a and Extended Data Fig. 2). Here, decomposition refers to the assimilation and mineralization of dead organic matter through microbial respiration, whereas engineering refers to the physical modification, maintenance or creation of habitats^1^ by faunal detritivory (see the Methods for further justification). These ten trophic functions are directly linked to various aspects of ecosystem functioning, including nutrient cycling and supply to plants, soil C cycling and sequestration, soil structure maintenance and biocontrol through predation^5,7,24^. For those calculated trophic functions for which direct measurements of corresponding ecosystem processes were available, we found good agreement between the two (Extended Data Fig. 3 and Supplementary Results). We then quantified soil food web multifunctionality^6,7^ by averaging the range-standardized values of these ten trophic functions.

To test our hypotheses, we performed Bayesian multi-level modelling to assess how tree community properties affect soil food web multifunctionality, while statistically controlling for potential confounding factors to ensure unbiased causal inferences (Extended Data Fig. 1c and Methods). We first adopted a traditional taxonomic approach to evaluate the effect of mixing three tree species and to quantify, by variance partitioning, the relative importance of tree species mixing, tree species composition (combination of species) and biogeography, that is, variation in abiotic conditions and associated tree species turnover across locations (Supplementary Fig. 2a). To gain deeper mechanistic insights, we also adopted a functional (trait-based) approach^14,29,30^ to assess the effects of both functional diversity and functional composition of tree communities, alongside biogeographic differences among locations. This enabled us to contrast patterns consistent with the ‘niche complementarity’ hypothesis, which states that functionally more diverse communities promote ecosystem functioning through greater complementarity effects^29^, with patterns consistent with the ‘mass ratio’ hypothesis, which states that ecosystem functioning is primarily driven by the mean attributes (trait values) of species weighted by their relative contribution to community biomass^31^.

**Extended Data Fig. 4:**
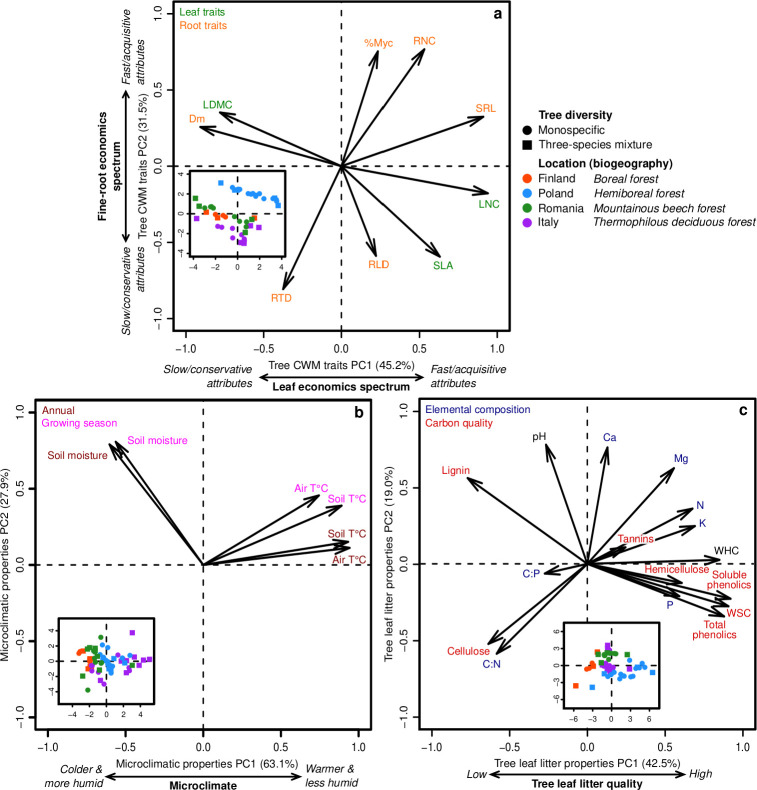
Principal Component Analysis (PCA) of tree functional composition based on community-level tree traits (a), microclimatic properties (b), and community-level tree leaf litter properties (c). Community-level tree traits and leaf litter values were expressed as community-weighted means (CWMs), weighted by relative basal area for tree traits and by leaf litterfall mass per tree species for leaf litter properties. Soil and air microclimate variables were measured at 8 cm depth and 50 cm above the ground, respectively. Variable loadings represent the correlations of variables with corresponding principal components. Insets show plot coordinates (*n* = 64 plots). Mean score values for the two first PCA axes of tree functional composition per species composition for each location are shown in Extended Data Table 1. Correlation and contribution of tree CWM traits to the principal components (panel a) are shown in Supplementary Table 7. LNC, leaf nitrogen content; SLA, specific leaf area; LDMC, leaf dry matter content; RNC, root nitrogen content; SRL, specific root length; RTD, root tissue density; Dm, mean root diameter; RLD, root length density; %Myc, ectomycorrhizal colonisation intensity; T°C, temperature; WSC, water soluble compounds; WHC, water holding capacity. Tannins are condensed tannins.

To achieve this, we characterized tree functional diversity and composition using three leaf traits and six fine-root traits commonly used to describe ecological strategies along the plant economics spectrum^9,32^, and which reflect a trade-off between resource acquisition (that is, fast strategy) and resource conservation (that is, slow strategy; Methods). Functional diversity was measured as the variability in trait values among tree species (functional dispersion), whereas functional composition was measured using the community-weighted mean (CWM) of trait values. We then reduced the complexity of tree functional composition to two dimensions (Extended Data Fig. 4a). The first dimension corresponded to the leaf economics spectrum (LES), which ranges from slow/conservative to fast/acquisitive leaf attributes^9^, and was also aligned here with a fine-root gradient which ranges from low to high belowground resource foraging efficiency. The second dimension corresponded to the fine-root economics spectrum (RES), which ranges from slow/conservative to fast/acquisitive fine-root attributes, and was also aligned here with a fine-root gradient of soil exploration strategies which ranges from ‘do-it-yourself’ to ‘outsourcing’ attributes^32^. To investigate the mechanisms underlying the effects of tree community properties on soil food web multifunctionality, we performed structural equation modelling incorporating a wide range of environmental drivers, including tree and understorey vegetation, microclimate, tree leaf litter quality and soil fertility (Extended Data Fig. 1c and Supplementary Fig. 3).

## Diversity effects on soil functioning

**Fig. 3:**
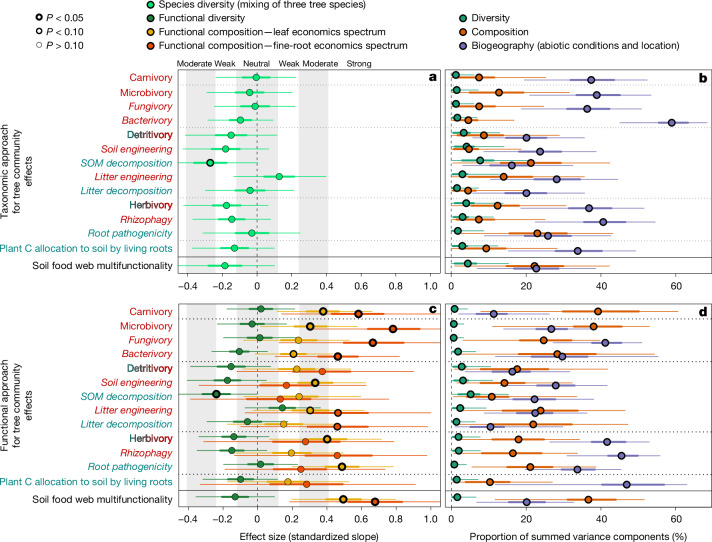
Effects of tree diversity and composition on soil food web functioning. **a**,**c**, Effect sizes of tree diversity and composition using taxonomic (**a**) and functional (**c**) approaches. Effect sizes calculated as partial slope coefficients standardized by the standard deviation. **b**,**d**, Decomposition of the total variance explained by tree diversity, tree composition and biogeography (that is, abiotic conditions and location), as determined by variance partitioning for the taxonomic (**b**) and functional (**d**) approaches. Variance components of each predictor group were standardized by the sum of all group-level variance components, including the residuals. Effects of diversity and composition of tree communities were estimated using both taxonomic (**a**,**b**) and functional (**c**,**d**) approaches based on Bayesian linear mixed-effects models (equations 1 and 2, respectively; *n* = 64 plots; Methods). Circles represent the posterior means, with thick and thin error bars representing 50% and 95% posterior credible intervals, respectively (posterior *n* = 10,000). The *P* values were derived from posterior distributions using a two-tailed test. Both the LES and RES representing tree functional composition ranged from slow/conservative to fast/acquisitive attributes (Extended Data Fig. 4a). Trophic functions are based on energy fluxes in the soil food web, aggregated by resource and consumer types (Fig. 1). Italicized functions are subsets of broader functions (not italicized) between the dotted lines. Trophic functions in blue-green and dark red indicate those mediated by microbes and fauna, respectively. Soil food web multifunctionality is the average of standardized values of each of the ten trophic functions of the soil food web. For statistical results, see Supplementary Table 1. SOM, soil organic matter.

Contrary to our first hypothesis, we found soil food web functioning to be little affected by tree species mixing and functional diversity (Fig. 3). The mixing of three tree species had rather negative effects on a number of trophic functions of the soil food web, that is, moderately negative for SOM decomposition, and weakly negative for plant C allocation to soil by living roots, rhizophagy and soil engineering (Fig. 3a and Supplementary Fig. 4). Tree species mixing also had a weakly positive effect on litter engineering, but neutral effects on the five remaining trophic functions. Similar results were observed for tree functional diversity (Figs. 2b and 3c), which is in contrast to what would be expected based on the ‘niche complementarity’ hypothesis^29^. Accordingly, tree species mixing and functional diversity had a weakly negative effect on soil food web multifunctionality (Fig. 3a,c), which was mediated through multiple mechanisms.

**Fig. 4:**
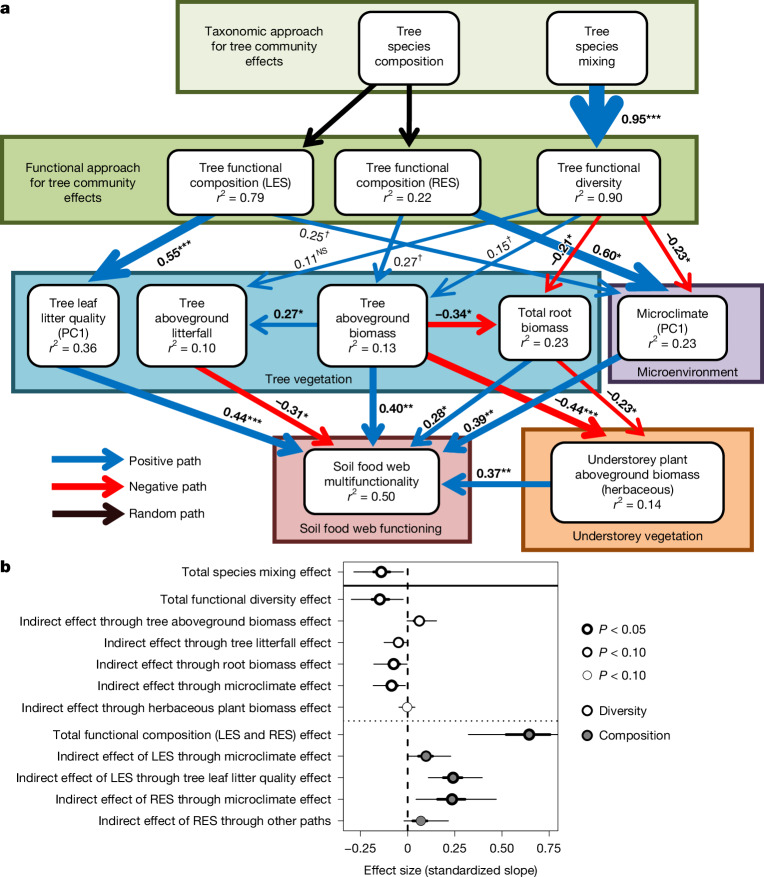
Mechanistic insights into the mediation of tree diversity and composition effects on soil food web functioning. **a**, Best-supported Bayesian multi-level structural equation model (*n* = 64 plots; Methods). Arrows indicate the direction of causality, and their widths are proportional to the path coefficients (posterior means of partial slope coefficients standardized by the standard deviation; posterior *n* = 10,000). The *r*^2^ values shown inside the boxes are the proportions of variance explained by explanatory variables present in this illustration. Both the leaf economics spectrum (LES) and fine-root economics spectrum (RES) representing tree functional composition ranged from slow/conservative to fast/acquisitive attributes (Extended Data Fig. 4a). ‘Microclimate’ ranged from colder and more humid to warmer and less humid (Extended Data Fig. 4b). ‘Tree leaf litter quality’ ranged from low to high nutritional quality (Extended Data Fig. 4c). The model was well supported by the data (Fisher’s *C* = 74.7, d.f. = 94, *P* = 0.929). None of the independence claims implied by the model was statistically significant at *α* = 0.05 (Supplementary Table 2). Relationships with abiotic context variables, and correlated errors between variables, are not shown here for clarity. See Supplementary Table 3 for further statistical results. Significant effects (*P* < 0.05) are reported in bold. ^NS^*P* > 0.10; ^†^*P* < 0.10; *P < 0.05; **P < 0.01; ***P < 0.001. **b**, Effect sizes of the indirect and total effects of tree diversity (white circles) and composition (grey circles) on soil food web multifunctionality, mediated by changes in tree and understorey plant communities, and microenvironment. Circles represent the posterior mean of effect sizes, with thick and thin error bars representing 50% and 95% posterior credible intervals, respectively (posterior *n* = 10,000). The *P* values were derived from posterior distributions using a two-tailed test. NS, not significant; PC, principal component.

First, tree species mixing negatively affected soil food web multifunctionality by both decreasing root biomass and increasing tree litterfall (Fig. 4), which shifted the resource-based energy channelling in the soil food web from living fine roots to litter (Fig. 2b and Supplementary Fig. 4). Previous studies have emphasized the key importance of living plant roots for soil microbes^33,34^ and fauna^35,36^, primarily through the provision of readily available resources such as rhizodeposits and photosynthates^34^. This finding therefore suggests that the apparent diversity-induced shift in tree resource allocation from belowground to aboveground^37,38^ modifies the overall quality of trophic resources for soil organisms towards less readily available energy, thereby impairing soil food web multifunctionality.

Second, tree species mixing negatively affected soil food web multifunctionality by cooling the forest microclimate at ground level, leading to a decrease in mean soil and air temperature (Fig. 4 and Extended Data Fig. 4b). This cooling effect is probably driven by an enhanced canopy packing^39,40^, which decreases radiative flux to the soil surface, and by a thicker litter layer that increases soil thermal insulation^41^. According to the ‘metabolic theory of ecology’^8^, metabolic rates of organisms are highly temperature-dependent. Consequently, the overall cooling of forest microclimate by trees^41,42^ could reduce the metabolic activity and energy demand of soil organisms, thereby reducing energy fluxes within the soil food web^43^. This finding supports the emerging view that microclimate modulation is an important, although often overlooked, mechanism contributing to plant diversity effects on ecosystem functioning^40^. An alternative explanation is that lower energy fluxes in mixed stands arise from bottom-up effects, whereby cooler ground-level conditions decrease belowground primary productivity and input of plant root litter, which in turn lead to a decline in secondary productivity and the metabolic demands of consumer populations. However, our data show that the effects of microclimate on soil food web multifunctionality occurred independently of root biomass (Fig. 4).

Previous studies have shown that tree species mixing can increase aboveground tree biomass and productivity through resource use complementary^37,44^. In line with this, we observed a 17% overyielding overall in aboveground tree biomass in three-species relative to single-species stands (Extended Data Table 1; see also Fig. 4a and Supplementary Table 1). Meanwhile, tree species mixing has previously been found to reduce the biomass of absorptive fine roots without affecting overall soil occupation, pointing to morphological root trait adjustments associated with higher resource use efficiency^38^. Consistent with this, the buffering of forest microclimate induced by tree species mixing^45^ was associated in our study with lower mean temperatures and reduced thermal energy at ground level. Our results indicate that these changes can collectively lead to a relatively weak decline in soil food web multifunctionality. Together, these findings suggest that tree diversity can exert contrasting effects on ecosystem functioning in different compartments of real-world forests^46^, whereby positive responses in aboveground productivity may coincide with, or even contribute to, negative responses belowground. It is important to note, however, that the diversity contrast in our study was limited to comparisons between single-species and three-species stands. Future research across broader gradients of tree species richness is therefore needed to better characterize the shape of the relationship between tree diversity and soil food web multifunctionality more generally. Although the relatively low species diversity tested here reflects typical conditions in European forest stands at the spatial scale that we considered^47^, it remains an open question how tree diversity influences soil food web multifunctionality in more diverse forests in other regions of the world, in which rare functional attributes may play a larger role in shaping soil food web processes.

## Composition effects on soil functioning

**Extended Data Fig. 5:**
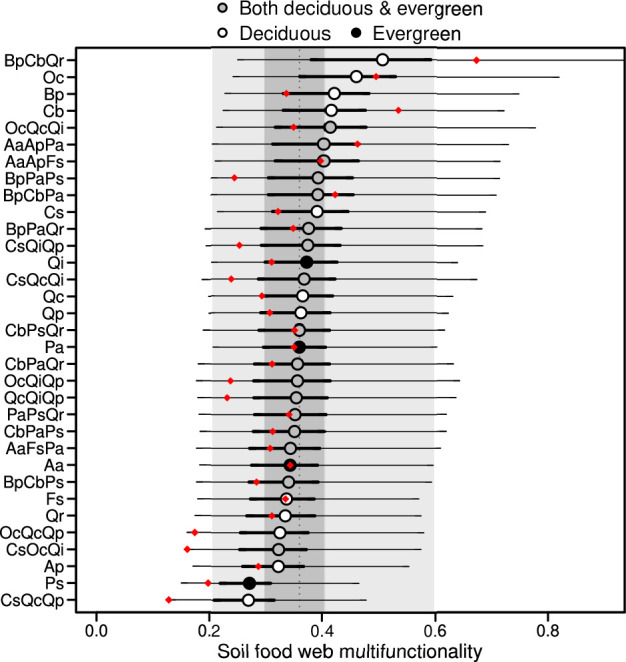
Random effects of tree species composition on soil food web multifunctionality. Circles represent the posterior means of predicted values derived from the Bayesian linear mixed-effects model used in the taxonomic approach (equation 1; *n* = 64 plots; see Methods). The dotted vertical line represents the general intercept of the model. Thick and thin error bars, along with dark and light grey filled area, represent 50% and 95% posterior credible intervals, respectively (posterior *n* = 10,000). Red diamonds represent the mean observed values of soil food web multifunctionality for each tree species composition. Aa, *Abies alba*; Ap, *Acer pseudoplatanus*; Bp, *Betula pendula*/*pubescens*, Cb, *Carpinus betulus*, Cs, *Castanea sativa*; Fs, *Fagus sylvatica*; Oc, *Ostrya carpinifolia*; Pa, *Picea abies*; Ps, *Pinus sylvestris*; Qc, *Quercus cerris*; Qi, *Q. ilex*; Qp, *Q. petraea*; Qr, *Q. robur*.

Our data show that tree species composition had a large influence on soil food web multifunctionality. The effect of species composition was over five times stronger than that of species mixing, accounting for 22.3% versus 4.4% of the summed variance components, respectively (Fig. 3b). This relatively minor role of species mixing per se, relative to species composition, is consistent with previous studies^46^, although it may partially reflect the limited range of tree species richness represented in our study. The effect of species composition within geographic locations was of similar magnitude to that of biogeography, which explained 22.7% of summed variance components. Tree species varied substantially in their effects, with species such as *Ostrya carpinifolia*, *Betula pendula*/*pubescens* and *Carpinus betulus* having positive effects and *Pinus sylvestris* having negative effects on soil food web multifunctionality (Extended Data Fig. 5). Tree compositional effects were especially strong for trophic functions such as root pathogenicity, litter engineering and SOM decomposition (Fig. 3b).

Soil food web multifunctionality was even more strongly associated with the functional composition of tree communities (quantified as CWM values of traits that are related to resource economics) than with species composition within geographic locations alone (36.7% versus 22.3% of summed variance components; Fig. 3b,d). This pattern is consistent with expectations derived from the ‘mass ratio’ hypothesis^31^, whereby ecosystem functioning is primarily determined by the traits of the most abundant species. Although complementarity may also contribute to ecosystem functioning, our analyses explicitly evaluated mass ratio effects through CWM traits alongside complementarity effects through trait dispersion, allowing these mechanisms to be considered as distinct, although potentially co-occurring, drivers^29^. The strong influence of functional composition probably reflects the fact that it also captures both species turnover and intraspecific variability across geographic locations. Moreover, unlike species composition, which considers only species presence or absence, functional composition incorporates the relative abundances of species within communities^29^. Functional composition also explained more of the variation in soil food web multifunctionality than did biogeography (20.1%) and functional diversity (1.6%; Fig. 3d). Further analyses showed a strong positive correlation between the variance components of functional composition and biogeography, suggesting that much of the biogeographic influence on soil food web multifunctionality is mediated through community-level variation in economic traits across locations (Extended Data Table 2 and Supplementary Results). Together, these results emphasize the importance of plant traits, in tandem with abiotic factors, in shaping the functioning of real-world ecosystems^46^.

In accordance with our second hypothesis, both dimensions of functional composition, that is, the LES and RES, had strong effects on soil food web multifunctionality. Specifically, tree communities dominated by species with fast/acquisitive attributes promoted higher soil food web multifunctionality than communities dominated by species with slow/conservative attributes (Fig. 3c). This finding provides empirical support for the theoretical framework proposing that community-level plant strategies related to economic traits play a key role in controlling soil food web functioning^5^. More broadly, it aligns with a growing body of evidence highlighting the importance of plant economic traits for terrestrial ecosystem functioning^9,48–51^. Positive effects of acquisitive attributes were observed across all trophic functions, including both lower and higher trophic levels (Fig. 3c,d). This pattern contrasts with earlier findings from experimental grasslands, in which the influence of plant community properties on consumer communities was found to weaken with increasing trophic level^16^.

The positive effects of leaf acquisitive attributes of tree communities were most pronounced for root pathogenicity, litter engineering, soil engineering and carnivory (Figs. 2c and 3c). We found that the effect of the LES on soil food web multifunctionality was mostly mediated by the quality of tree leaf litter (Fig. 4 and Extended Data Fig. 4c). Specifically, tree communities with acquisitive leaf attributes produced leaf litter of higher nutritional quality, which in turn promoted greater soil food web multifunctionality. There is a broad consensus that litter quality plays a key role as a major driver of soil food webs^5,52^, and our study provides empirical evidence that one of the primary ways that plant economic traits control soil food web functioning is through the nutritional quality of the litter they produce^2,5^.

**Extended Data Fig. 6:**
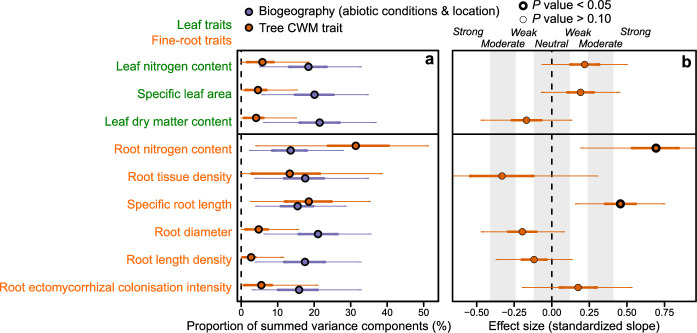
Community-weighted means (CWMs) of tree traits as drivers of soil food web multifunctionality. a, Decomposition of the total variance explained by community-weighed means of each tree trait, and biogeography (abiotic conditions and location), as determined by variance partitioning. Variance components of each predictor group were standardized by the sum of all group-level variance components, including the residuals. b, Effect sizes calculated as partial slope coefficients standardized by standard deviation. Circles represent the posterior means, with thick and thin error bars representing 50% and 95% posterior credible intervals, respectively (posterior *n* = 10,000). Effects of tree CWM traits were estimated based on Bayesian linear mixed-effects models (equation 3; *n* = 64 plots; see Methods). The *p*-values were derived from posterior distributions using a two-tailed test.

The positive effects of fine-root acquisitive attributes of tree communities on trophic functions were particularly strong for rhizophagy, litter decomposition, litter engineering, bacterivory, fungivory and carnivory (Figs. 2c and 3c). Notably, we found that the effect of the RES on soil food web multifunctionality was mostly mediated by the forest microclimate, with a similar but weaker pattern observed for the effects of the LES (Fig. 4 and Extended Data Fig. 4b). Specifically, tree communities with acquisitive attributes were associated with a warmer and less humid microclimate, that is, higher mean soil and air temperature and lower mean soil moisture. This could potentially be explained by lower interception of solar radiation by their canopies and/or higher water uptake by their roots^9,53,54^, the latter leading to a lower cooling effect of water evaporation (as an endothermic process) within the soil^41^. A warmer forest microclimate can, in turn, promote soil food web multifunctionality by increasing the metabolic activity of soil organisms^8^. These findings emphasize that plant economic traits can also control soil food web functioning indirectly through their effects on micro-environmental conditions^1,40^. Further examination of the effects of individual CWM traits revealed that soil food web multifunctionality was best explained by fine-root nitrogen content, which had a strongly positive effect, accounting for 31.4% of the summed variance components (Extended Data Fig. 6). This result suggests that the positive effect of fine-root acquisitive attributes of tree communities may also be linked to the higher nutritional quality of root litter^2,5^. Fine roots of acquisitive plant species, which have higher nitrogen content and greater metabolic activity, are also associated with increased rhizodeposition rates, thereby stimulating microbial activity in the rhizosphere^27,55^.

## Conclusions

Our study provides empirical evidence that tree communities control the functioning of belowground food webs across European forests, mainly through compositional effects related to the community-level leaf and fine-root economic traits. We found that tree communities dominated by species with fast/acquisitive attributes promoted soil food web multifunctionality compared with those dominated by species with slow/conservative attributes. This effect was mainly due to the higher nutritional quality of the trophic resources and the warmer forest microclimate that they provide. Both the species and functional composition of tree communities had large effects on soil food web multifunctionality, comparable in magnitude to biogeographic variation across locations. By contrast, tree species mixing (three-species versus single-species stands) and functional diversity had much smaller and, generally, negative effects reflecting a shift in energy channelling from fine roots to litter, alongside a cooling effect on forest microclimate. This was observed despite tree species mixing having positive effects on aboveground tree biomass production, highlighting the contrasting responses of aboveground and belowground subsystems to higher plant diversity.

Our findings carry important implications for forest management and biodiversity policy. Simply promoting tree species mixing may not necessarily enhance ecosystem functioning belowground even if it stimulates aboveground productivity. Instead, strategic selection of tree species based on their functional traits is likely to be more effective in sustaining the multiple functions provided by soil food webs. However, as climate change is expected to intensify drought and temperature stress, widespread tree mortality is anticipated^56,57^, particularly among more acquisitive tree species^58^. Beyond the immediate loss of ecosystem functionality following forest die-back, such mortality could drive long-term shifts in tree communities towards more drought-tolerant, conservative species, either through natural dynamics or climate-adaptive management^56^. Our study indicates that these compositional shifts could slow down soil food web processes, with cascading consequences for nutrient cycling, C turnover and forest regeneration. Altogether, this study highlights the value of combining food web energetics with trait-based approaches to predict ecosystem responses to environmental change. Incorporating these insights into forest policy and management will be critical for designing resilient forest systems that maintain multifunctionality into the future.

## Methods

### Study sites and sampling design

We used a pan-European network of 64 mature, uneven-aged forest plots (30 × 30 m^2^) consisting of three-species mixture stands (34 plots) and corresponding monospecific stands (30 plots; Extended Data Table 1). These plots are part of the FunDivEUROPE (Functional Significance of Forest Biodiversity in Europe) exploratory platform^59^, and were established across European forests over 2011–2012 to investigate the role of the diversity and composition of regionally common and economically important tree species on ecosystem functioning. The studied plots were distributed across four locations featuring different European forest types and spanning a large biogeographic gradient: North Karelia (Finland), Białowieża (Poland), Râşca (Romania) and Colline Metallifere (Italy), corresponding to typical boreal, hemi-boreal, mountainous beech and thermophilous deciduous (Mediterranean) forests, respectively (Supplementary Table 4 and Supplementary Fig. 5a). In each location, plots were carefully selected based on tree species richness and composition while minimizing as much as possible covariation with potentially confounding environmental factors such as topography and soil conditions^59^ (Supplementary Fig. 2a). Plot selection was performed so as to include monospecific stands of all tree species from the local species pool and replicate the three-species mixture treatment with different tree species combinations while maximizing community evenness (Extended Data Table 1). This allowed strict avoidance of a dilution gradient, such as would occur in a design with monospecific stands of only one species combined with mixture stands including this species, along with a clear distinction between the effects of species mixing and composition. Our stratified plot selection procedure enabled us to mimic formal biodiversity experiments, given that such manipulative approaches are virtually impossible to undertake in mature forests owing to the high longevity of tree species. Tree species diversity and composition in the studied plots were predominantly the result of natural community assembly from the regional species pool, combined with local forest management practices. The investigated levels of species richness, that is, one versus three tree species, are typical for European forest ecosystems (https://forest.eea.europa.eu/topics/forest-biodiversity-and-ecosystems/forest-ecosystems), boreal forests in Asia and North-America, as well as managed forests and plantations worldwide, although clearly much less diversified than many (sub)tropical forests and some mid-latitude temperate forests^47^. Overall, our sampling design encompassed a total pool of 13 tree species, including 12 ectomycorrhizal and one arbuscular mycorrhizal tree species, and the local species pool ranged from three to five tree species per location (Extended Data Table 1 and Supplementary Table 4).

### Soil organism sampling and analysis

In each plot, we assessed energy fluxes through the soil food web by measuring the biomass of major groups of organisms within this food web^22,23,43,60^, including both microbial (bacteria and fungi) and faunal (nematodes, microarthropods and macroinvertebrates) groups. Biomass data for all soil organism groups were expressed per unit surface area (g dry weight m^−2^) at the plot level. Further details on biomass calculation and the trophic classification of soil organisms are provided in the Supplementary Methods.

#### Sampling

Soil organisms were sampled in all plots during the phenological spring of 2017 (Supplementary Table 4), a period of high soil biological activity. We sampled both the litter layer (unfragmented aboveground litter, OL horizon) and the soil layer (including both fragmented/humified organic matter and mineral soil, OF/OH/A horizons). In each plot, we selected five 10 × 10-m^2^ subplots, with samples taken equidistantly from three trees of either the same species in monospecific stands or of different species for three-species mixture stands (Supplementary Fig. 5b). For each subplot, soil samples for microbial analyses and nematode extraction were collected by taking five soil cores (10-cm depth, 5.3-cm diameter), spaced approximately 35 cm apart around the equidistant point between the three trees, weighted by tree individual size, that is, individual diameter at breast height. The five cores were gently sieved through 6-mm mesh (to avoid damaging nematodes), homogenized and pooled at the subplot level for nematode extraction. Pooled soil was then sieved through 2-mm mesh for microbial analyses. All subsamples were stored at 4 °C until further processing. For microarthropod extraction, an intact core (10-cm depth, 10-cm diameter), including both the litter and soil layers, was collected from each of three subplots along a southwest–northeast transect and stored at 4 °C until further processing. For the hand-sorting of soil macroinvertebrates, an intact monolith (25-cm depth, 25 × 25-cm^2^ surface), including both the litter and soil layers, was collected from each of the same three subplots. To express all data per unit surface area, an extra core was sampled, sieved through 2-mm mesh, dried at 105 °C for 48 h and weighted to measure soil bulk density.

#### Microorganisms

The biomass of bacteria (gram-positive and gram-negative), arbuscular mycorrhizal fungi and non-arbuscular mycorrhizal fungi was quantified at the plot level using phospholipid fatty acid data^61^. Fungal community data based on metagenomic amplicon sequencing and bioinformatics^62^ were used to partition non-arbuscular mycorrhizal fungal biomass into five trophic guilds: ericoid mycorrhizal fungi, ectomycorrhizal fungi, general saprotrophic fungi, wood saprotrophic fungi and plant pathogenic fungi (Supplementary Methods). The biomass of each fungal trophic guild was calculated by multiplying its relative abundance, that is, number of reads divided by the total number of reads for the five trophic guilds, by the total non-arbuscular mycorrhizal fungal biomass.

#### Nematodes

Nematodes were extracted for each subplot within 72 h after sampling from approximately 100 g of fresh soil using a modified sugar flotation method^63^, before being heat-killed and fixed in 4% formaldehyde. Nematodes were then pooled and counted at the plot level, and a subsample of approximately 160 randomly selected individuals were identified to family level. The biomass of nematode families was calculated based on body mass data retrieved from the Nemaplex database (http://nemaplex.ucdavis.edu). Nematode families were assigned to five trophic guilds^64^: herbivores, bacterivores, fungivores, omnivores and carnivores.

#### Microarthropods

Microarthropods were extracted from the intact core (including both the litter and soil layers) within 72 h after sampling for each subplot using the Berlese–Tullgren funnel method^65^, and were fixed in 70% ethanol. Microarthropods were then counted and identified to species level for collembola and to order level for mites. Microarthropod biomass was estimated based on an allometric model using body length data retrieved from the BETSI database (https://portail.betsi.cnrs.fr) for collembola, and data from the literature for mites^60^. Microarthropod taxa were assigned to seven trophic guilds^64^ belonging to the following broad trophic groups: microbi-detritivores, fungivores, omnivores and carnivores.

#### Macroinvertebrates

Macroinvertebrates were hand sorted in the field for each subplot and fixed in 70% ethanol. Macroinvertebrates were then counted and identified to species level for Lumbricidae, Isopoda, Diplopoda, Chilopoda and Araneae; and order to family level for other taxa^66^. All macroinvertebrate individuals were weighed for body mass. Macroinvertebrates were assigned to 23 trophic guilds^64^ belonging to the following broad trophic groups: herbivores, detritivores, humi-detritivores, omnivores and carnivores.

### Metabolic rates

We used soil microbial respiration and biomass data^67^ to calculate the metabolic rates of microbial groups, whereas we used a model based on individual body mass, environmental temperature (mean soil temperature during the growing season; see ‘Microclimate’ section below) and phylogenetic grouping^68^ to calculate the metabolic rates of faunal groups (Supplementary Methods).

### Assimilation efficiencies

Assimilation efficiencies (that is, the proportion of consumed food assimilated by digestion) specific to each food type (plant-derived resource or prey) for faunal consumers were calculated using a model based on food N content^69^, and we also applied a temperature correction of assimilation efficiency^70^ (Supplementary Methods). Assimilation efficiencies of all food resources were set to 1 for microbial consumers, given their external digestion system.

### Plant-derived resources

In each plot, we quantified the biomass of the following plant-derived (basal) resources of the soil food web: living fine roots^38^ (absorptive roots belonging the first three root orders) and associated photosynthates/rhizodeposits^34^, litter (including dead leaves^67^, dead roots^71^ and dead wood) and SOM^72^ (Supplementary Methods). Photosynthates/rhizodeposits refer to organic compounds provided by roots to mycorrhizal fungi, or released directly by roots into the soil. To estimate the biomass of rhizodeposits, we used a mass ratio of 0.4 between net rhizodeposition (the portion of rhizodeposited C remaining in the soil after microbial utilization and respiration) and root biomass, based on the results of a meta-analysis of fixed C partitioning in plant–soil systems^34^. The biomass of rhizodeposits was calculated by multiplying the biomass of living fine roots by this factor.

### Food web reconstruction

We constructed our soil food web from 47 network nodes, including six plant-derived (basal) resources and 41 trophic guilds of soil organisms (consumers) that were differentiated based on multiple traits^64^ (Supplementary Table 5). To establish the topology of the soil food web and quantify trophic interaction strengths (Supplementary Fig. 1), a food web interaction matrix was constructed based on basic food web principles, and a priori knowledge of soil organism biology and key traits of consumers following the approach in ref. ^7^, except that microbes were considered here as consumers rather than basal resources (Supplementary Methods). Briefly, the food web interaction matrix was calculated by multiplying five matrices representing different trait dimensions: phylogenetically defined feeding preferences, density dependence, predator–prey interactions related to body mass ratio and hunting strategy, prey defence mechanisms and spatial niche overlap related to vertical stratification. The five matrices relied on the following assumptions, respectively^7^: (1) there are phylogenetically conserved differences in feeding preferences of consumers; (2) food consumption is density (biomass) dependent, that is, consumers will preferentially feed on food resources that are locally abundant owing to a higher encounter rate; (3) the strength of predator–prey interactions is primarily defined by the optimum predator–prey mass ratio, that is, a predator is typically larger than its prey, but certain predator hunting traits can modify the optimum predator–prey mass ratio; (4) the strength of predator–prey interactions can be weakened by prey defence traits, that is, prey with efficient physical, chemical or behavioural protection are consumed less; (5) the strength of trophic interactions between a consumer and its food resource is modulated by the overlap in their spatial niches related to vertical stratification, with greater overlap leading to a stronger interaction. Food web reconstruction was carried out separately for each plot to account for plot-specific density dependence.

### Food web energy fluxes and functions

For the calculation of energy fluxes, we assumed a steady state of the soil food web^6^. This means that the energy flowing into a given feeding guild of the food web through food consumption balances the energy lost by excretion, metabolism and predation of that feeding guild. Energy fluxes to each feeding guild within the food web (kJ m^−2^ d^−1^) were then calculated based on the trophic interaction matrix using the following equation^6^: $F=\frac{1}{{e}_{{\rm{a}}}}\times (X+L)$, where *F* is the total flux of energy into the feeding guild, *e*_a_ is the diet-specific assimilation efficiency, *X* is the community metabolism of the feeding guild and *L* is the energy loss to predation (see the Supplementary Methods for further details). To simplify the representation of the food web, we aggregated biomass and energy flux matrices at broader trophic group levels by summing the rows and columns of trophic nodes belonging to the same trophic group (Fig. 1 and Supplementary Table 5).

We then calculated five broad trophic functions of the soil food web (Fig. 1): carnivory, the sum of energy fluxes outgoing from fauna to their faunal consumers; microbivory, the sum of energy fluxes outgoing from microbes to their faunal consumers; herbivory, the sum of energy fluxes outgoing from living fine roots to their consumers; plant C allocation to soil by living roots, the sum of energy fluxes outgoing from photosynthates and rhizodeposits (via living fine roots) to their microbial consumers; and detritivory, the sum of energy fluxes outgoing from detritus (dead organic matter, including plant litter and SOM) to their consumers. Additionally, we calculated eight more specific trophic functions (Fig. 1): bacterivory and fungivory, the sum of energy fluxes outgoing from bacteria and fungi to their faunal consumers, respectively; root pathogenicity and rhizophagy, the sum of energy fluxes outgoing from living fine roots to their microbial and faunal consumers, respectively; litter decomposition and litter engineering, the sum of energy fluxes outgoing from plant litter to their microbial and faunal consumers, respectively; and SOM decomposition and soil engineering, the sum of energy fluxes outgoing from SOM to their microbial and faunal consumers, respectively. Decomposition refers to the assimilation and mineralization of dead organic matter through respiration, a process that is mediated mainly by microbes in soil^60^. Engineering refers to the physical modification, maintenance or creation of habitats^1^, which is a major way through which soil faunal detritivory affects the decomposition, transformation and translocation of dead organic matter^7,73^. Such inferences from energy fluxes about effects that are not purely trophic are especially justifiable in soil where habitat and food resources are tightly interlinked^7^. As such, the faunal consumption of litter results in the conversion of litter into faeces which in turn accelerates its decomposition through chemical and physical changes^73^, as a form of litter engineering. Similarly, the faunal consumption of SOM is linked to biopedturbation and soil structure maintenance^73^ in a manner that represents soil engineering.

We quantified soil food web multifunctionality based on ten trophic functions: plant C allocation to soil by living roots, root pathogenicity, rhizophagy, litter decomposition, litter engineering, SOM decomposition, soil engineering, bacterivory, fungivory and carnivory. Defining high multifunctionality by fast rates of multiple functions simultaneously, we calculated soil food web multifunctionality using the ‘averaging’ approach^74^, that is, the main range-standardized values for the ten trophic functions (Supplementary Methods). Consistent results were found when using the alternative ‘threshold’ approach^74^ (see ‘Sensitivity analyses’ in the Supplementary Methods). Additionally, we adopted the ‘single functions’ approach to help illuminate which individual functions drive trends in the effects of tree communities on soil food web multifunctionality^74^.

### Tree functional (trait-based) properties

Functional diversity and composition of tree communities were characterized using a set of nine plant functional traits known to be directly involved in resource economics^9,32^. These traits included three leaf traits: leaf nitrogen content, specific leaf area and leaf dry matter content; and six fine (absorptive) root traits^71^: root nitrogen content, root tissue density, specific root length, mean root diameter, root length density and ectomycorrhizal colonisation intensity. Trait data of each tree species were mostly derived from plot-specific measurement in the field, but specific leaf area and leaf dry matter content data for Poland and Italy were retrieved from the TRY plant trait database^75^ (https://www.try-db.org). Using trait values from databases has limitations because of intraspecific trait variability. However, the sensitivity analysis that we performed revealed that the associated uncertainty in trait values had little bearing on our overall inferences (see ‘Sensitivity analyses’ in the Supplementary Methods). See the Supplementary Methods for further details and Supplementary Table 6 for trait values of each tree species and geographic location.

To quantify functional diversity, we calculated the functional dispersion (FDis) index corresponding to the mean distance of each species to the centroid of all species within the multidimensional trait space^76^. For functional composition, we calculated CWMs of each trait. Both FDis and CWM values were computed based on the relative basal area of tree species. We performed a principal component analysis (PCA) on all CWM traits, which simplified functional composition into two dimensions^71^ (Extended Data Fig. 4a and Supplementary Table 7): (1) an LES (45.2% of variation), which ranged from slow/conservative leaf attributes (N-poor and dry-matter-rich leaves with low leaf area per unit mass) to fast/acquisitive leaf attributes (N-rich and dry-matter-poor leaves with high leaf area per unit mass), and also aligned here with a fine-root gradient of belowground resource foraging strategies ranging from low foraging efficiency (thick fine roots with low length per unit mass) to high foraging efficiency (thin fine roots with high length per unit mass); (2) an RES (31.5% of variation), which ranged from slow/conservative fine-root attributes (N-poor fine roots with low tissue density) to fast/acquisitive fine-root attributes (N-rich fine roots with high tissue density), and also aligned here with a fine-root gradient of soil exploration strategies ranging from ‘do-it-yourself’ attributes (high root length density and low ectomycorrhizal colonisation intensity) to ‘outsourcing’ attributes (low root length density and high ectomycorrhizal colonisation intensity). The mean values of FDis and the scores of the two first PCA axes of CWM trait ordination for each location are shown in Extended Data Table 1.

### Tree and understorey vegetation

We characterized forest vegetation using a set of eight variables measured in each plot: tree aboveground biomass, tree aboveground litterfall, total root biomass, aboveground biomass of both woody and herbaceous understorey plants, and aboveground C:N ratio and species richness of understorey plant communities (Supplementary Methods).

### Environmental drivers

#### Abiotic conditions

We characterized abiotic conditions in each plot using a set of six variables related to soil texture, macroclimate and topography: the sand, silt and clay content of mineral soil (A horizon, 10-cm depth), mean annual temperature and precipitation, and altitude (Supplementary Methods). We performed a PCA to reduce the dimensionality of abiotic properties into two dimensions (Supplementary Fig. 2a): (1) a soil texture and macroclimate gradient ranging from coarse soil texture, and dry and cold macroclimate to fine soil texture, and wet and hot macroclimate (70.8% of variation); (2) a temperature gradient ranging from cold macroclimate with high elevation to warm macroclimate with low elevation (19.1% of variation).

#### Microclimate

We characterized microclimate using a set of six variables: soil temperature and moisture (measured at 8-cm soil depth) and air temperature (measured 50 cm above the ground) for both annual and growing season (daily mean temperatures > 5 °C) periods (Supplementary Methods). We performed a PCA to simplify microclimatic properties into a single dimension ranging from cold and wet to hot and dry (Extended Data Fig. 4b; 63.1% of variation).

#### Leaf litter quality

We characterized freshly fallen tree leaf litter quality using a set of 16 chemical and physical variables mainly related to elemental composition and C quality^77^: the concentrations of N, P, Ca, Mg and K, and the C:N and C:P ratios, the proportion of C fractions (lignin, cellulose, hemicellulose and water-soluble compounds), the concentrations of secondary metabolites (condensed tannins, total phenolics and soluble phenolics), as well as the litter pH and water holding capacity. Leaf litter quality data of each tree species were derived from location-specific measurement (Supplementary Methods).

We then quantified the functional diversity and composition of tree leaf litter by calculating the FDis index and CWMs of each litter property based on the relative leaf litter mass of the component tree species. We performed a PCA to simplify tree leaf litter properties into one dimension ranging from low to high nutritional quality (Extended Data Fig. 4c; 42.5% of variation).

#### Soil fertility

We characterized soil fertility using a set of six variables measured in each plot^72^: the organic C content of mineral soil (A horizon, 10-cm depth), the mass of the forest floor (including unfragmented aboveground litter and fragmented/humified organic matter, OL/OF/OH horizons), as well as the pH and C:N ratios of both the forest floor and mineral soil (Supplementary Methods). We performed a PCA to simplify soil properties into a single dimension ranging from low to high fertility (Supplementary Fig. 2b; 40.1% of variation).

### Measured ecosystem processes

To characterize in situ patterns of litter decomposition, we used field-based data of naturally occurring tree leaf litter decomposition measured in each plot^78^ (Supplementary Methods). To characterize SOM decomposition, we further used soil microbial respiration and biomass data^67^ (Supplementary Methods). To characterize soil and litter engineering, we used humus type and forest floor mass data^72^ as these two variables reflect the transformation and translocation of dead organic matter by faunal detritivory^73^.

### Statistical analyses

All analyses were performed using R v.4.3.0 (ref. ^79^). To test how tree community properties affect soil food web functioning, we used Bayesian multi-level models that accounted for the hierarchical design of the study, with plots nested within four geographic locations across Europe. We used random intercept models (with varying intercepts but a common slope across locations) to assess general patterns across European forests. Similar results were found when using random slope models (with varying intercepts but a common slope across locations), indicating that our findings are robust to model specification (see ‘Sensitivity analyses’ in the Supplementary Methods). To ensure that the effects of tree communities were not biased by confounding factors, we also explicitly included abiotic covariates into the models, that is, the first two PCA axes representing abiotic conditions (Supplementary Fig. 2a). This statistical adjustment allowed us to control for variation in abiotic conditions both among and within locations. Following the principle of the ‘structural causal model’ framework, controlling for confounding factors allows to satisfy the ‘backdoor criterion’ required for quantifying unbiased causal relationships from observational data^80^. We also checked whether omitted variable bias generated by potential unmeasured confounders at the location level could affect our inference, and found that it was not an issue in our study (see ‘Sensitivity analyses’ in the Supplementary Methods). We adopted both taxonomic and functional approaches. For the taxonomic approach, we used linear mixed-effects models (LMMs) including tree species richness (comparing three-species mixture stands to corresponding monospecific stands) and abiotic covariates (see above) as fixed factors, and tree species composition (a factor listing all tree species present; Extended Data Table 1) and location as random factors, as in the following equation:

1 $$\begin{array}{c}{y}_{ij}=\alpha +{\beta }_{1}{{\rm{r}}{\rm{i}}{\rm{c}}{\rm{h}}{\rm{n}}{\rm{e}}{\rm{s}}{\rm{s}}}_{ij}+{\beta }_{2}{{\rm{a}}{\rm{b}}{\rm{i}}{\rm{o}}{\rm{t}}{\rm{i}}{\rm{c\; PC}}1}_{ij}+{\beta }_{3}{{\rm{a}}{\rm{b}}{\rm{i}}{\rm{o}}{\rm{t}}{\rm{i}}{\rm{c\; PC}}2}_{ij}+{\delta }_{ij}^{{\rm{c}}{\rm{o}}{\rm{m}}{\rm{p}}{\rm{o}}{\rm{s}}{\rm{i}}{\rm{t}}{\rm{i}}{\rm{o}}{\rm{n}}}+{\delta }_{i}^{{\rm{l}}{\rm{o}}{\rm{c}}{\rm{a}}{\rm{t}}{\rm{i}}{\rm{o}}{\rm{n}}}+{{\epsilon }}_{ij}\\ {\rm{w}}{\rm{i}}{\rm{t}}{\rm{h}}\,{\delta }_{ij}^{{\rm{c}}{\rm{o}}{\rm{m}}{\rm{p}}{\rm{o}}{\rm{s}}{\rm{i}}{\rm{t}}{\rm{i}}{\rm{o}}{\rm{n}}} \sim N(0,{{\sigma }}_{{\rm{c}}{\rm{o}}{\rm{m}}{\rm{p}}{\rm{o}}{\rm{s}}{\rm{i}}{\rm{t}}{\rm{i}}{\rm{o}}{\rm{n}}}^{2}),{\delta }_{i}^{{\rm{l}}{\rm{o}}{\rm{c}}{\rm{a}}{\rm{t}}{\rm{i}}{\rm{o}}{\rm{n}}}\, \sim N(0,{{\sigma }}_{{\rm{l}}{\rm{o}}{\rm{c}}{\rm{a}}{\rm{t}}{\rm{i}}{\rm{o}}{\rm{n}}}^{2})\\ \,{\rm{a}}{\rm{n}}{\rm{d}}\,{{\epsilon }}_{ij} \sim N(0,{{\sigma }}^{2})\end{array}$$

where *i* refers to location and *j* refers to plot, *y* is the dependent variable, *α* is the general intercept, *β*_*x*_ terms are partial slopes, *δ* terms are random effects, that is, factor-specific deviation from the common intercept, *ϵ* is the model error, that is, residuals, and *σ*^2^ is the variance. For the functional approach, we used LMMs including tree functional diversity (FDis), tree functional composition (first two PCA axes of tree CWM traits corresponding, respectively, to the LES and RES; Extended Data Fig. 4a) and abiotic covariates (see above) as fixed factors, and location as a random factor, as in the following equation:

2 $$\begin{array}{c}{y}_{{ij}}=\alpha +{\beta }_{1}{\mathrm{FDis}}_{{ij}}+{\beta }_{2}{\mathrm{CWM\; trait\; PC}1}_{{ij}}+{\beta }_{3}{\mathrm{CWM\; trait\; PC}2}_{{ij}}\\ \,+{\beta }_{4}{\mathrm{abiotic\; PC}1}_{{ij}}+{\beta }_{5}{\mathrm{abiotic\; PC}2}_{{ij}}+{\delta }_{i}^{\mathrm{location}}+{\varepsilon }_{{ij}}\\ \,\mathrm{with}\,{\delta }_{i}^{\mathrm{location}} \sim N(0,{\sigma }_{\mathrm{location}}^{2})\,\mathrm{and}\,{{\epsilon }}_{{ij}} \sim N(0,{\sigma }^{2})\end{array}$$

To further investigate the effects of functional composition, we also used LMMs including individual tree CWM trait and abiotic covariates as fixed factors, and location as a random factor, as in the following equation:

3 $$\begin{array}{c}{y}_{{ij}}={\beta }_{0}+{\beta }_{1}{\mathrm{CWM\; trait}}_{{ij}}+{\beta }_{2}{\mathrm{abiotic\; PC}1}_{{ij}}\\ \,+{\beta }_{3}{\mathrm{abiotic\; PC}2}_{{ij}}+{\delta }_{i}^{\mathrm{location}}+{\varepsilon }_{{ij}}\\ \mathrm{with}\,{\delta }_{i}^{\mathrm{location}} \sim N(0,{\sigma }_{\mathrm{location}}^{2})\,\mathrm{and}\,{{\epsilon }}_{{ij}} \sim N(0,{\sigma }^{2})\,\end{array}$$

Bayesian LMMs were fitted with Markov chain Monte Carlo methods with non-informative priors for all parameters. Each model was fitted based on 10,000 iterations of both warm-up and sampling phases and was checked for their convergence and compliance with statistical assumptions, including normality and independence of residuals, normality of random effects, homogeneity of variance, linearity of relationships and absence of multicollinearity (Supplementary Methods).

For each model, we performed variance partitioning following a model-based approach based on variance components^81^ to quantify the proportion of variance in the dependent variable explained by three groups of predictors: diversity (tree species richness or FDis), composition (tree species composition or CWM traits PC1 and PC2) and biogeography (abiotic conditions PC1 and PC2, and location). To minimize the influence of multicollinearity within each group (especially between abiotic covariates and location in the biogeography group; Supplementary Fig. 2a), we performed variance partitioning at the level of variable groups. This was done by summing the variance terms of all predictors within each group^81^. This allowed us to incorporate the covariances between group predictors to the variance component of that group, thereby reducing the covariance between variance components at the variable group level. Variance components of each group were standardized by the sum of all group-level variance components, including the residuals. To assess how covariance between group-level variance components might affect total variance accounting^81^, we computed further variance partitioning metrics: (1) basic variance partition, which expresses the variance of each group standardized by the total variance of the dependent variable; (2) marginal variance partition, which quantifies the contribution of each group while accounting for covariances with all other groups; and (3) partial variance partition, which quantifies the contribution of each group that cannot be explained by a linear combination of the remaining groups. Further methodological details are provided in the Supplementary Methods.

We calculated the effect size of fixed factors as the slope coefficient standardized by the standard deviation (Supplementary Methods). We quantified the uncertainty of effect size based on credible intervals. Standardized slope (*β*_st_) values of |*β*_st_| < 0.12, 0.12 ≤ |*β*_st_| < 0.24, 0.24 ≤ |*β*_st_| < 0.41 and |*β*_st_| ≥ 0.41 were interpreted as neutral, weak, moderate and strong effects, respectively. We used *P* values of slope coefficients and Bayes factors as measures of evidence for fixed and random effects, respectively. The *P* values were derived from posterior distributions using a two-tailed test^82^. Bayes factors were used to quantify the support for models including the random factor tested, compared with null models without the random factor. Bayes factors were calculated as the ratio of marginal likelihoods of the two models (Supplementary Methods).

To test a priori multivariate causal hypotheses regarding how tree community effects on soil food web multifunctionality are mediated by changes in tree and understorey plant properties and microenvironment^83,84^, we performed piecewise multi-level structural equation modelling (SEM)^85^. We first constructed a causal diagram based on ecological theory and our previous knowledge of the system^85^ (Extended Data Fig. 1c and Supplementary Fig. 3) and built an initial SEM model (Supplementary Table 8). Following a local estimation approach^85^, we tested hypothesized causal relationships by fitting component LMMs with random intercepts across locations using Bayesian modelling, along with the use of variance partitioning and effect size computation methods in a manner similar to those described above. For each component LMM, abiotic covariates (see above) were included to ensure that mediation effects were not biased by confounding factors^84^, thereby satisfying the ‘backdoor criterion’. The initial SEM model was then simplified by removing unsupported linkages^85^ using information-theoretic methods to select the most parsimonious SEM model. For each component LMM within the initial SEM model, stepwise backward selection was performed by removing the predictor with the highest *P* value, verifying at each step that model simplification improved goodness-of-fit using Pareto smoothed importance-sampling leave-one-out cross-validation^86^ (Supplementary Methods). To test the goodness-of-fit of the selected SEM model, we used Shipley’s test of directional separation^87^, which assesses whether any important causal pathways may be missing (Supplementary Methods). To quantify and compare the magnitude of tree community effects on soil food web multifunctionality, indirect effects were calculated as the product of the standardized coefficients along each path. Total effects were calculated as the sum of indirect effects.

### Reporting summary

Further information on research design is available in the Nature Portfolio Reporting Summary linked to this article.

## Online content

Any methods, additional references, Nature Portfolio reporting summaries, source data, extended data, supplementary information, acknowledgements, peer review information; details of author contributions and competing interests; and statements of data and code availability are available at 10.1038/s41586-026-10455-1.

## Supplementary information

Supplementary Information Supplementary Methods, results, Figs. 1–6, Tables 1–19 references.

Reporting Summary

Peer Review file

## Figures and Tables

**Extended Data Table 1:**
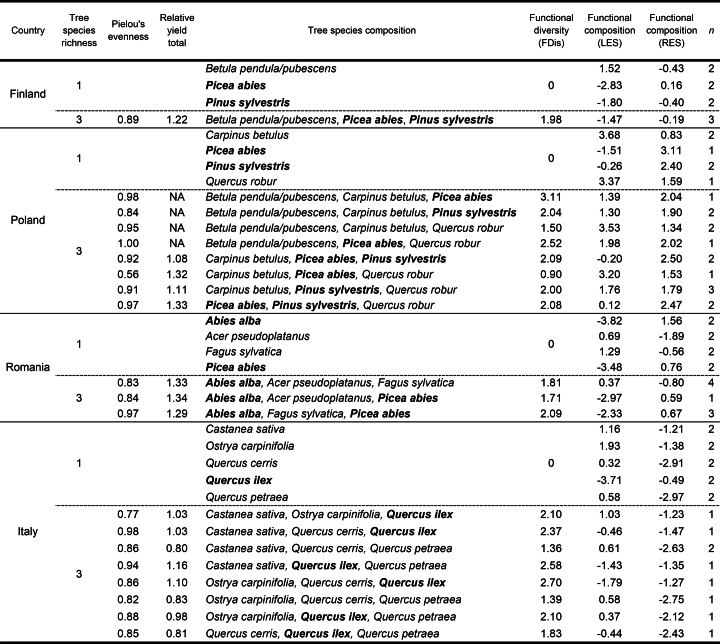
Sampling design of the study

The table summarizes the richness and composition of the sampled tree communities. Mean values are provided when applicable. Evergreen species are shown in bold. The relative yield of a species measures its aboveground biomass in a multi-species community as a proportion of its aboveground biomass in single-species community, and the relative yield total of a multi-species community is the sum of the relative yield of all component species^89^. The mean RYT was 1.17 when taking equal weight for all countries, indicating a 17% increase in aboveground biomass in mixed stands (overyielding). This is in line with previous studies showing that tree species mixing increases tree aboveground biomass at the stand-level by approximately 25%, mostly due to complementary effects^44^. *n* indicates the number of plots for each tree species composition per country.

**Extended Data Table 2:**
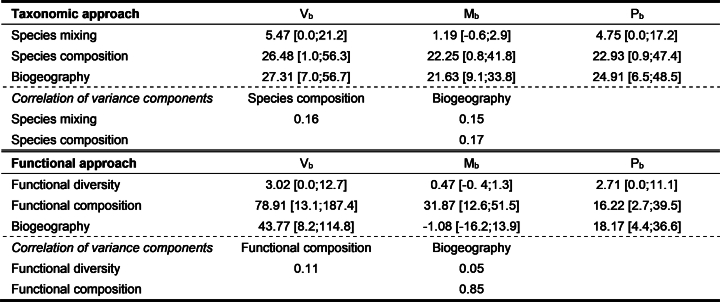
Alternative variance partitioning metrics from Bayesian multi-level models testing tree community effects on soil food web multifunctionality, using both taxonomic and functional approaches

Three complementary metrics are reported: V_b_ (*basic variance partition*), defined as the variance of a group of model components standardized by the total variance in soil food web multifunctionality; M_b_ (*marginal variance partition*), which quantifies the contribution of a group of model components while accounting for covariances with all other groups; and P_b_ (*partial variance partition*), which quantifies the unique contribution of a group of model components not explained by a linear combination of the others. Differences between V_b_ and P_b_ indicate covariance, and thus potential confounding, between groups of model components. Differences between V_b_ and M_b_ reflects whether covariance increase or suppress the variability of the linear predictor, *i.e*. the combined effect of all model components^81^. All values are expressed as percentages. Further details on the computation of these metrics are provided in the Supplementary Methods.

## Data Availability

All data generated or analysed during this study are available at Figshare (10.6084/m9.figshare.31700881)^88^. This study also used previously published data available from the following databases: FunDivEUROPE (https://data.botanik.uni-halle.de/fundiveurope), Nemaplex (http://nemaplex.ucdavis.edu), BETSI (https://portail.betsi.cnrs.fr), TRY Plant Trait Database (https://www.try-db.org), DriloBASE (https://drilobase.org/drilobase), UNITE (https://unite.ut.ee) and WorldClim (https://www.worldclim.org).

## Code availability

All R scripts for computation of food-web energy fluxes and statistical analyses are available at Figshare (10.6084/m9.figshare.31700881)^88^.

## References

[1] Jones, C. G., Lawton, J. H. & Shachak, M. Positive and negative effects of organisms as physical ecosystem engineers. *Ecology* **78**, 1946–1957 (1997).

[2] Cebrian, J. Patterns in the fate of production in plant communities. *Am. Nat.* **154**, 449–468 (1999). 10523491 10.1086/303244

[3] Cardinale, B. J. et al. The functional role of producer diversity in ecosystems. *Am. J. Bot.* **98**, 572–592 (2011). 21613148 10.3732/ajb.1000364

[4] Eisenhauer, N. et al. A multitrophic perspective on biodiversity–ecosystem functioning research. *Adv. Ecol. Res.* **61**, 1–54 (2019). 10.1016/bs.aecr.2019.06.001 PMC6944504 31908360

[5] Wardle, D. A. et al. Ecological linkages between aboveground and belowground biota. *Science* **304**, 1629–1633 (2004). 15192218 10.1126/science.1094875

[6] Barnes, A. D. et al. Energy flux: the link between multitrophic biodiversity and ecosystem functioning. *Trends Ecol. Evol.* **33**, 186–197 (2018). 29325921 10.1016/j.tree.2017.12.007 PMC6181201

[7] Potapov, A. M. Multifunctionality of belowground food webs: resource, size and spatial energy channels. *Biol. Rev.* **97**, 1691–1711 (2022). 35393748 10.1111/brv.12857

[8] Brown, J. H., Gillooly, J. F., Allen, A. P., Savage, V. M. & West, G. B. Toward a metabolic theory of ecology. *Ecology* **85**, 1771–1789 (2004).

[9] Reich, P. B. The world-wide ‘fast–slow’ plant economics spectrum: a traits manifesto. *J. Ecol.* **102**, 275–301 (2014).

[10] Dornelas, M. et al. Assemblage time series reveal biodiversity change but not systematic loss. *Science* **344**, 296–299 (2014). 24744374 10.1126/science.1248484

[11] Bardgett, D. G. & Wardle, D. A. *Aboveground-Belowground Linkages: Biotic Interactions, Ecosystems Processes, and Global Change* (Oxford Univ. Press, 2010).

[12] Chapin, F. S. et al. Consequences of changing biodiversity. *Nature* **405**, 234–242 (2000). 10821284 10.1038/35012241

[13] Hooper, D. U. et al. Effects of biodiversity on ecosystem functioning: a consensus of current knowledge. *Ecol. Monogr.* **75**, 3–35 (2005).

[14] Reiss, J., Bridle, J. R., Montoya, J. M. & Woodward, G. Emerging horizons in biodiversity and ecosystem functioning research. *Trends Ecol. Evol.* **24**, 505–514 (2009). 19595476 10.1016/j.tree.2009.03.018

[15] Vandermeer, J., Lawrence, D., Symstad, A. & Hobbie, S. In *Biodiversity and Ecosystem Functioning: Synthesis and Perspectives* (eds Loreau, M. et al.) 221–233 (Oxford Univ. Press, 2002).

[16] Scherber, C. et al. Bottom-up effects of plant diversity on multitrophic interactions in a biodiversity experiment. *Nature* **468**, 553–556 (2010). 20981010 10.1038/nature09492

[17] Schuldt, A. et al. Multiple plant diversity components drive consumer communities across ecosystems. *Nat. Commun.* **10**, 1460 (2019). 30926809 10.1038/s41467-019-09448-8 PMC6440984

[18] Wan, N.-F. et al. Global synthesis of effects of plant species diversity on trophic groups and interactions. *Nat. Plants* **6**, 503–510 (2020). 32366981 10.1038/s41477-020-0654-y

[19] Lindeman, R. L. The trophic-dynamic aspect of ecology. *Ecology* **23**, 399–417 (1942).

[20] Moore, J. C. & de Ruiter, P. C. *Energetic Food Webs: An Analysis of Real and Model Ecosystems* (Oxford Univ. Press, 2012).

[21] Barnes, A. D. et al. Biodiversity enhances the multitrophic control of arthropod herbivory. *Sci. Adv.* **6**, eabb6603 (2020). 33158860 10.1126/sciadv.abb6603 PMC7673711

[22] Buzhdygan, O. Y. et al. Biodiversity increases multitrophic energy use efficiency, flow and storage in grasslands. *Nat. Ecol. Evol.* **4**, 393–405 (2020). 32094542 10.1038/s41559-020-1123-8

[23] Yi, H. et al. Belowground energy fluxes determine tree diversity effects on above- and belowground food webs. *Curr. Biol.* **35**, 1870–1882 (2025). 40209707 10.1016/j.cub.2025.03.034

[24] de Vries, F. T. et al. Soil food web properties explain ecosystem services across European land use systems. *Proc. Natl. Acad. Sci. USA* **110**, 14296–14301 (2013). 23940339 10.1073/pnas.1305198110 PMC3761618

[25] Kulmatiski, A. et al. Most soil trophic guilds increase plant growth: a meta-analytical review. *Oikos* **123**, 1409–1419 (2014).

[26] Crowther, T. W. et al. The global soil community and its influence on biogeochemistry. *Science* **365**, eaav0550 (2019). 31439761 10.1126/science.aav0550

[27] Henneron, L., Cros, C., Picon-Cochard, C., Rahimian, V. & Fontaine, S. Plant economic strategies of grassland species control soil carbon dynamics through rhizodeposition. *J. Ecol.* **108**, 528–545 (2020).

[28] Wardle, D. A. & Van der Putten, W. H. In *Biodiversity and Ecosystem Functioning: Synthesis and Perspectives* (eds Loreau, M. et al.) 155–168 (Oxford Univ. Press, 2002).

[29] Díaz, S. et al. Incorporating plant functional diversity effects in ecosystem service assessments. *Proc. Natl. Acad. Sci. USA* **104**, 20684–20689 (2007). 18093933 10.1073/pnas.0704716104 PMC2410063

[30] Chacón-Labella, J. et al. How to improve scaling from traits to ecosystem processes. *Trends Ecol. Evol.* **38**, 228–237 (2023). 36435672 10.1016/j.tree.2022.10.007

[31] Grime, J. P. Benefits of plant diversity to ecosystems: immediate, filter and founder effects. *J. Ecol.* **86**, 902–910 (1998).

[32] Weigelt, A. et al. An integrated framework of plant form and function: the belowground perspective. *New Phytol.* **232**, 42–59 (2021). 34197626 10.1111/nph.17590

[33] Högberg, P. et al. High temporal resolution tracing of photosynthate carbon from the tree canopy to forest soil microorganisms. *New Phytol.* **177**, 220–228 (2008). 17944822 10.1111/j.1469-8137.2007.02238.x

[34] Pausch, J. & Kuzyakov, Y. Carbon input by roots into the soil: quantification of rhizodeposition from root to ecosystem scale. *Glob. Change Biol.* **24**, 1–12 (2018). 10.1111/gcb.13850 28752603

[35] Pollierer, M. M., Langel, R., Körner, C., Maraun, M. & Scheu, S. The underestimated importance of belowground carbon input for forest soil animal food webs. *Ecol. Lett.* **10**, 729–736 (2007). 17594428 10.1111/j.1461-0248.2007.01064.x

[36] Zhou, Z. et al. Plant roots fuel tropical soil animal communities. *Ecol. Lett.* **26**, 742–753 (2023). 36857203 10.1111/ele.14191

[37] Jucker, T., Bouriaud, O., Avacaritei, D. & Coomes, D. A. Stabilizing effects of diversity on aboveground wood production in forest ecosystems: linking patterns and processes. *Ecol. Lett.* **17**, 1560–1569 (2014). 25308256 10.1111/ele.12382

[38] Wambsganss, J., Beyer, F., Freschet, G. T., Scherer-Lorenzen, M. & Bauhus, J. Tree species mixing reduces biomass but increases length of absorptive fine roots in European forests. *J. Ecol.* **109**, 2678–2691 (2021).

[39] Jucker, T., Bouriaud, O. & Coomes, D. A. Crown plasticity enables trees to optimize canopy packing in mixed-species forests. *Funct. Ecol.* **29**, 1078–1086 (2015).

[40] Beugnon, R. et al. Microclimate modulation: an overlooked mechanism influencing the impact of plant diversity on ecosystem functioning. *Glob. Change Biol.* **30**, e17214 (2024). 10.1111/gcb.17214 38494864

[41] Geiger, R., Aron, R. H. & Todhunter, P. *The Climate Near the Ground* (Rowman & Littlefield, 2009).

[42] De Frenne, P. et al. Global buffering of temperatures under forest canopies. *Nat. Ecol. Evol.* **3**, 744–749 (2019). 30936433 10.1038/s41559-019-0842-1

[43] Schwarz, B. et al. Warming alters energetic structure and function but not resilience of soil food webs. *Nat. Clim. Change* **7**, 895–900 (2017). 10.1038/s41558-017-0002-z PMC5714267 29218059

[44] Feng, Y. et al. Multispecies forest plantations outyield monocultures across a broad range of conditions. *Science* **376**, 865–868 (2022). 35587983 10.1126/science.abm6363

[45] Schnabel, F. et al. Tree diversity increases forest temperature buffering via enhancing canopy density and structural diversity. *Ecol. Lett.* **28**, e70096 (2025). 40119529 10.1111/ele.70096 PMC11928777

[46] van der Plas, F. Biodiversity and ecosystem functioning in naturally assembled communities. *Biol. Rev.* **94**, 1220–1245 (2019). 30724447 10.1111/brv.12499

[47] Hordijk, I. et al. Dominance and rarity in tree communities across the globe: patterns, predictors and threats. *Glob. Ecol. Biogeogr.* **33**, e13889 (2024).

[48] Henneron, L., Kardol, P., Wardle, D. A., Cros, C. & Fontaine, S. Rhizosphere control of soil nitrogen cycling: a key component of plant economic strategies. *New Phytol.* **228**, 1269–1282 (2020). 32562506 10.1111/nph.16760

[49] Freschet, G. T., Aerts, R. & Cornelissen, J. H. C. A plant economics spectrum of litter decomposability. *Funct. Ecol.* **26**, 56–65 (2012).

[50] Hu, Z. et al. Traits drive global wood decomposition rates more than climate. *Glob. Change Biol.* **24**, 5259–5269 (2018). 10.1111/gcb.14357 29901246

[51] Yan, P., He, N., Yu, K., Xu, L. & Van Meerbeek, K. Integrating multiple plant functional traits to predict ecosystem productivity. *Commun. Biol.* **6**, 239 (2023). 36869238 10.1038/s42003-023-04626-3 PMC9984401

[52] Wardle, D. A. & Lavelle, P. in *Driven by Nature – Plant Litter Quality and Decomposition* (eds Cadisch, G. & Giller, K. E.) 107–127 (CAB International, 1997).

[53] Martin-Guay, M.-O. et al. Tree identity and diversity directly affect soil moisture and temperature but not soil carbon ten years after planting. *Ecol. Evol.* **12**, e8509 (2022). 35136558 10.1002/ece3.8509 PMC8809433

[54] Gillerot, L. et al. Forest structure and composition alleviate human thermal stress. *Glob. Change Biol.* **28**, 7340–7352 (2022). 10.1111/gcb.16419 36062391

[55] Han, M. et al. Linking rhizosphere soil microbial activity and plant resource acquisition strategy. *J. Ecol.* **111**, 875–888 (2023).

[56] Millar, C. I. & Stephenson, N. L. Temperate forest health in an era of emerging megadisturbance. *Science* **349**, 823–826 (2015). 26293954 10.1126/science.aaa9933

[57] Anderegg, W. R. L., Kane, J. M. & Anderegg, L. D. L. Consequences of widespread tree mortality triggered by drought and temperature stress. *Nat. Clim. Change* **3**, 30–36 (2013).

[58] Greenwood, S. et al. Tree mortality across biomes is promoted by drought intensity, lower wood density and higher specific leaf area. *Ecol. Lett.* **20**, 539–553 (2017). 28220612 10.1111/ele.12748

[59] Baeten, L. et al. A novel comparative research platform designed to determine the functional significance of tree species diversity in European forests. *Perspect. Plant Ecol. Evol. Syst.* **15**, 281–291 (2013).

[60] Petersen, H. & Luxton, M. A comparative analysis of soil fauna populations and their role in decomposition processes. *Oikos* **39**, 287–388 (1982).

[61] Prada-Salcedo, L. D., Wambsganss, J., Bauhus, J., Buscot, F. & Goldmann, K. Low root functional dispersion enhances functionality of plant growth by influencing bacterial activities in European forest soils. *Environ. Microbiol.* **23**, 1889–1906 (2021). 32959469 10.1111/1462-2920.15244

[62] Prada-Salcedo, L. D. et al. Fungal guilds and soil functionality respond to tree community traits rather than to tree diversity in European forests. *Mol. Ecol.* **30**, 572–591 (2021). 33226697 10.1111/mec.15749

[63] Jenkins, W. R. B. A rapid centrifugal-flotation technique for separating nematodes from soil. *Plant Dis. Report.* **48**, 692 (1964).

[64] Potapov, A. M. et al. Feeding habits and multifunctional classification of soil-associated consumers from protists to vertebrates. *Biol. Rev.* **97**, 1057–1117 (2022). 35060265 10.1111/brv.12832

[65] Coleman, D. C. & Crossley, D. A. J. *Fundamentals of Soil Ecology* (Academic Press, 2004).

[66] Ganault, P. et al. Relative importance of tree species richness, tree functional type, and microenvironment for soil macrofauna communities in European forests. *Oecologia* **196**, 455–468 (2021). 33959812 10.1007/s00442-021-04931-w

[67] Gillespie, L. M. et al. Tree species mixing affects soil microbial functioning indirectly via root and litter traits and soil parameters in European forests. *Funct. Ecol.* **35**, 2190–2204 (2021).

[68] Ehnes, R. B., Rall, B. C. & Brose, U. Phylogenetic grouping, curvature and metabolic scaling in terrestrial invertebrates. *Ecol. Lett.* **14**, 993–1000 (2011). 21794052 10.1111/j.1461-0248.2011.01660.x

[69] Jochum, M. et al. Decreasing stoichiometric resource quality drives compensatory feeding across trophic levels in tropical litter invertebrate communities. *Am. Nat.* **190**, 131–143 (2017). 28617641 10.1086/691790

[70] Lang, B., Ehnes, R. B., Brose, U. & Rall, B. C. Temperature and consumer type dependencies of energy flows in natural communities. *Oikos* **126**, 1717–1725 (2017).

[71] Wambsganss, J. et al. Tree species mixing causes a shift in fine-root soil exploitation strategies across European forests. *Funct. Ecol.* **35**, 1886–1902 (2021).

[72] Dawud, S. M. et al. Is tree species diversity or species identity the more important driver of soil carbon stocks, C/N ratio, and pH? *Ecosystems* **19**, 645–660 (2016).

[73] Angst, G. et al. Conceptualizing soil fauna effects on labile and stabilized soil organic matter. *Nat. Commun.* **15**, 5005 (2024). 38886372 10.1038/s41467-024-49240-x PMC11183196

[74] Byrnes, J. E. K. et al. Investigating the relationship between biodiversity and ecosystem multifunctionality: challenges and solutions. *Methods Ecol. Evol.* **5**, 111–124 (2014).

[75] Kattge, J. et al. TRY plant trait database – enhanced coverage and open access. *Glob. Change Biol.* **26**, 119–188 (2020). 10.1111/gcb.14904 31891233

[76] Laliberté, E. & Legendre, P. A distance-based framework for measuring functional diversity from multiple traits. *Ecology* **91**, 299–305 (2010). 20380219 10.1890/08-2244.1

[77] Joly, F.-X. et al. Tree species diversity affects decomposition through modified micro-environmental conditions across European forests. *New Phytol.* **214**, 1281–1293 (2017). 28181238 10.1111/nph.14452

[78] Joly, F.-X., Scherer-Lorenzen, M. & Hättenschwiler, S. Resolving the intricate role of climate in litter decomposition. *Nat. Ecol. Evol.* **7**, 214–223 (2023). 36624177 10.1038/s41559-022-01948-z

[79] R Core Team. *R: A Language and Environment for Statistical Computing* (R Foundation for Statistical Computing, 2022).

[80] Arif, S. & MacNeil, M. A. Applying the structural causal model framework for observational causal inference in ecology. *Ecol. Monogr.* **93**, e1554 (2023).

[81] Schulz, T., Saastamoinen, M. & Vanhatalo, J. Model-based variance partitioning for statistical ecology. *Ecol. Monogr.* **95**, e1646 (2025).

[82] Shi, H. & Yin, G. Reconnecting p-value and posterior probability under one- and two-sided tests. *Am. Stat.* **75**, 265–275 (2021).

[83] Grace, J. B. & Irvine, K. M. Scientist’s guide to developing explanatory statistical models using causal analysis principles. *Ecology* **101**, e02962 (2020). 31872426 10.1002/ecy.2962

[84] Correia, H. E., Dee, L. E. & Ferraro, P. J. Designing causal mediation analyses to quantify intermediary processes in ecology. *Biol. Rev.* **100**, 1512–1533 (2025). 40059404 10.1111/brv.70011

[85] Grace, J. B., Scheiner, S. M. & Schoolmaster Jr, D. R. in *Ecological Statistics: Contemporary Theory and Application* (eds Fox, G. A. et al.) 168–199 (Oxford Univ. Press, 2015).

[86] Vehtari, A., Gelman, A. & Gabry, J. Practical Bayesian model evaluation using leave-one-out cross-validation and WAIC. *Stat. Comput.* **27**, 1413–1432 (2017).

[87] Shipley, B. Confirmatory path analysis in a generalized multilevel context. *Ecology* **90**, 363–368 (2009). 19323220 10.1890/08-1034.1

[88] Henneron, L, et al. Dataset & R codes – Tree community resource economics control soil food web multifunctionality. *f**igshare*10.6084/m9.figshare.31700881 (2026). 10.1038/s41586-026-10455-1 PMC13322958 42092141

[89] Hector, A. The effect of diversity on productivity: detecting the role of species complementarity. *Oikos* **82**, 597–599 (1998).

