# Supplementary material for: Tree community resource economics control soil food web multifunctionality
Source: Nature. 2026 May 6;655(8121):133–40. doi: 10.1038/s41586-026-10455-1 (PMC13322958; doi:10.1038/s41586-026-10455-1)
Supplement: Supplementary file 1 — Supplementary Methods, results, Figs. 1–6, Tables 1–19 references. [file 41586_2026_10455_MOESM1_ESM.pdf]

nature portfolio

https://doi.org/10.1038/s41586-026-10455-1

Supplementary information

Tree community resource economics control soil food web multifunctionality

In the format provided by the
authors and unedited

Supplementary Information

Tree community resource economics control soil food web multifunctionality

Ludovic Henneron[1,2*], David A. Wardle[3], Matty P. Berg[4,5], Stephan Hättenschwiler[6], Jürgen Bauhus[7], François Buscot[8,9], Sylvain Coq[6], Thibaud Decaëns[6], Nathalie Fromin[6], Pierre Ganault[6,2], Lauren M. Gillespie[6], Kezia Goldmann[8], Radim Matula[10], Alexandru Milcu[6,11], Bart Muys[12], Johanne Nahmani[6], Luis Daniel Prada-Salcedo[8,9], Michael Scherer-Lorenzen[13], Kris Verheyen[14], Janna Wambsganss[7,15], Paul Kardol[1,16]

[1]Department of Forest Ecology and Management, Swedish University of Agricultural Sciences, Umeå, Sweden
[2]ECODIV USC 1499, Univ Rouen Normandie, INRAE, Rouen, France
[3]Department of Ecology, Environment and Geoscience, Umeå University, Umeå, Sweden
[4]Amsterdam Institute for Life and Environment, Section Ecology and Evolution, Vrije Universiteit, Amsterdam, the Netherlands
[5]GELIFES, Community Conservation Group, Groningen University, Groningen, The Netherlands
[6]CEFE, Univ Montpellier, CNRS, EPHE, IRD, Montpellier, France
[7]Chair of Silviculture, Faculty of Environment and Natural Resources, University of Freiburg, Freiburg, Germany
[8]Department of Soil Ecology, UFZ - Helmholtz Centre for Environmental Research, Halle (Saale), Germany
[9]German Centre for Integrative Biodiversity Research (iDiv) Halle-Jena-Leipzig, Leipzig, Germany
[10]Department of Forest Ecology, Faculty of Forestry and Wood Sciences, Czech University of Life Sciences, Prague, Czech Republic
[11]Montpellier European Ecotron, Univ Montpellier, CNRS, Campus Baillarguet, Montferrier-sur-Lez, France
[12]Department of Earth & Environmental Sciences, KU Leuven, Leuven, Belgium
[13]Geobotany, Faculty of Biology, University of Freiburg, Freiburg, Germany
[14]Forest & Nature Lab, Department of Environment, Ghent University, Melle-Gontrode, Belgium
[15]Research Institute for Forest Ecology and Forestry (FAWF), Landesforsten Rheinland-Pfalz, Trippstadt, Germany
[16]Department of Forest Mycology and Plant Pathology, Swedish University of Agricultural Sciences, Uppsala, Sweden

Table of content

Supplementary Methods 3
Biomass calculation and trophic classification of soil organisms 3
Microorganisms 3
Soil fauna 4
Calculation of metabolic rates and assimilation efficiencies 5
Plant-derived resources 6
Reconstruction of the soil food web topology and interaction strengths 7
Calculation of soil food web energy fluxes, trophic functions and multifunctionality 8
Tree functional (trait-based) diversity and composition 9
Tree and understorey vegetation 10
Environmental drivers 11
Abiotic conditions 11
Microclimate 11
Leaf litter quality 11
Soil fertility 12
Measured ecosystem processes 12
Statistical analyses 13
Sensitivity analyses 13

Supplementary Results 16
Linkages between calculated soil food web functions and measured soil functions 16
Exploration of the effects of correlation between variance components 17

Supplementary Figures and Tables 18
Supplementary Fig. 1. Graphical illustration of the food web topology and trophic interaction strengths among trophic guilds 18
Supplementary Fig. 2. Ordination of abiotic conditions, and soil properties. 19
Supplementary Fig. 3. Causal diagram of the *a priori* hypotheses about the mediation of tree community effects on soil food web multifunctionality 20
Supplementary Fig. 4. Effects of tree species mixing on energy fluxes and trophic group biomasses of the soil food web across European forests 21
Supplementary Fig. 5. Map of study site location across Europe, and illustration of the sampling design 22
Supplementary Fig. 6. Results of the sensitivity analysis about tree trait value uncertainty 23
Supplementary Table 1. Results of Bayesian multi-level models testing tree community effects on soil food web multifunctionality 24
Supplementary Table 2. Basis set testing all conditional independence claims of the SEM model 26
Supplementary Table 3. Results of the best-supported Bayesian multi-level SEM model testing the mediation of tree community effects on soil food web multifunctionality 28
Supplementary Table 4. Study site characteristics 29
Supplementary Table 5. List of plant-derived (basal) resources and trophic guilds with trait values used for food web reconstruction 30
Supplementary Table 6. Functional trait values of tree species for each location. 32
Supplementary Table 7. Ordination of tree trait community-weighted means. 33
Supplementary Table 8. Initial full SEM model 34
Supplementary Table 9. Nematode families, their trophic guild, and their mean body mass 35
Supplementary Table 10. Collembola species, their trophic guild and their mean body mass 36
Supplementary Table 11. Earthworm species identified, and their ecological groups 39
Supplementary Table 12. Regression parameters used to calculate metabolic rates for faunal consumers. 40
Supplementary Table 13. Diet-specific assimilation efficiency values for faunal consumers 41
Supplementary Table 14. Parameters to calculate temperature correction of assimilation efficiency 42
Supplementary Table 15. Multicollinearity checking 43
Supplementary Table 16. Results of tree community effects using the 'average' and 'threshold' approaches ... 44
Supplementary Table 17. Results of Bayesian multi-level random-slope models 45
Supplementary Table 18. Sensitivity analysis checking for omitted variable bias 46
Supplementary Table 19. Adjacency matrix of feeding preferences 47

Supplementary References 48

Supplementary Methods

Biomass calculation and trophic classification of soil organisms

Microorganisms

Bacterial and fungal dry biomass in each plot was measured using phospholipid fatty acid (PLFA) analysis[1] as described by Prada-Salcedo *et al.* (2021)[2]. Briefly, soil samples were stored at -20 °C within 72 h after sampling, and PLFAs were extracted from 2 g of freeze-dried soil pooled at the plot-level using the method of Bligh & Dyer (1959)[3]. Individual PLFAs were then identified and quantified by GC/MS (Agilent, HP DB5 column), and were converted to nmol PLFA per g of dry soil. The PLFAs i15:0, a15:0, 15:0, i16:0, 16:1ω7, 16:1ω9, i17:0, a17:0, 17:0, and 18:1ω7 were pooled to quantify bacterial biomass using a conversion factor of 363.6 nmol PLFA per 1 mg C[1]. We also separated between bacterial PLFAs specific to gram-negative (16:1ω7, and 18:1ω7), and gram-positive (i15:0, a15:0, 10Me15:0, i16:0, 10Me16:0, i17:0, a17, 10Me17:0, and 10Me18:0)[4]. The ratio of gram-positive to gram-negative bacterial PLFAs was then used to partition their respective biomass from bacterial biomass. The PLFA 16:1ω5 was used to quantify arbuscular mycorrhizal (AM) fungal biomass using a conversion factor of 38.0 nmol PLFA per 1 mg C[5]. The PLFAs 18:2ω6,9 and 18:1ω9 were pooled to quantify non-AM fungal biomass using a conversion factor of 20.06 nmol PLFA per 1 mg C[6]. Microbial C mass per unit surface area (g C m^{-2}) was then calculated by multiplying microbial C mass per unit soil mass (mg C g^{-1} dry soil) with soil bulk density (g cm^{-3}) and soil volume per unit surface area (cm^3 m^{-2}). We finally converted microbial C mass into microbial dry mass assuming a C content of 46 and 41% of dry matter for bacteria and fungi, respectively[7]. Across all plots, the ratio of fungal to bacterial biomass C was found to be 9.9 ± 4.8 (median ± standard deviation), which is close to the previously reported value of 8.5 based a meta-analysis using PLFA data[8]. The median of total microbial biomass was found to be 93 ± 66 g C microbial m^{-2}, which is within the same range than previously reported values for forest ecoystems[9] (51 ± 6 for boreal forests, 89 ± 26 for temperate coniferous forests, and 82 ± 9 for temperate deciduous forests).

Plot-specific fungal community data based on metagenomic sequencing and bioinformatics analyses[10] performed according to Prada-Salcedo *et al.* (2021)[11] were used to partition non-AM fungal biomass into five trophic guilds: ericoid mycorrhizal fungi, ectomycorrhizal fungi, general saprotrophic fungi, wood saprotrophic fungi, and plant pathogenic fungi. Briefly, soil DNA was extracted from samples pooled at the plot-level, and the internal transcribed spacer (ITS) region 2 was amplified by PCR using the primers P5-5N-ITS4, P5-6N-ITS4, P7-3N-fITS7, and P7-4N-fITS7. Amplicons were sequenced using an Illumina MiSeq System according to standard protocols, and the sequences were processed using the metabarcoding amplicon pipeline DeltaMP, and clustered into operational taxonomic units (OTUs) according to the UNITE database[12]. The OTUs were then assigned to trophic guilds using the FUNGuild tool[13], and the biomasses of each fungal trophic guild were calculated by multiplying their relative abundance (number of reads of the trophic guild divided by the total number of reads for all five trophic guilds) by the total non-AM fungal biomass[14–16]. The OTUs that potentially switch between trophic modes during their life history and OTUs that lacked sufficient taxonomic classification for functional description were not considered for the calculation of relative abundance. The biomass of mycorrhizal fungi was calculated as the sum of ericoid mycorrhizal, ectomycorrhizal and arbuscular mycorrhizal fungal biomass, while the biomass of decomposer fungi was calculated as the sum of general and wood saprotrophic fungal biomass. On average across all plots, we found mycorrhizal fungi, followed by saprotrophic fungi, to be the dominant trophic guilds in term of contribution to fungal biomass, in accordance with previous studies on forest stands dominated by ectomycorrhizal tree species[14,16,17].

Soil fauna

For each plot, we measured the dry mass of each faunal guild. Because the calculation of faunal metabolic rates was based on a model using faunal fresh mass[18], we also calculated fresh mass of each faunal guild.

The fresh mass of nematode families per unit soil mass (µg g^{-1} dry soil) was calculated by multiplying their density (number of individuals g^{-1} dry soil) with individual fresh body mass values (µg $individual^{-1}$) retrieved from the Nemaplex database (http://nemaplex.ucdavis.edu, Supplementary Table 9). These individual fresh body mass values are the mean of all species values available for each family, which were calculated using the following allometric equation[19]:

$$M = \frac{L \times D^2}{1.6 \times 10^6} \qquad (1)$$

where M is the fresh mass (in µg) per individual, L is the nematode length (in µm) and D is the greatest body diameter at the largest body part (in µm). Nematode biomass per unit surface area (µg m^{-2}) was computed based on soil bulk density as described above, and we converted nematode fresh mass to dry mass assuming a dry matter content of 25%[20]. Nematode families were assigned to five trophic guilds: herbivores, bacterivores, fungivores, omnivores, and carnivores[21] (Supplementary Table 9).

The dry mass per unit surface area of microarthropods was calculated by multiplying their density (individual number m^{-2}) with individual dry body mass values (µg $individual^{-1}$). For collembola species, the mean individual dry body mass (M, in µg) was calculated from the mean body length (L, in mm) retrieved from the BETSI database (https://portail.betsi.cnrs.fr/) using the following allometric equation (Supplementary Table 10):

$$M = aL^{\mathrm{b}} \qquad (2)$$

where a is the normalisation coefficient and b is the scaling exponent. Abdomen length of Symphypleona was used in the original equations and was assumed to be 0.83 of the total body length. Two sets of coefficients derived from two independent studies[22,23] were used for each species (a_1, b_1 and a_2, b_2) and the two estimates of dry body mass were averaged[24]. Collembola fresh body mass was calculated from the resulting average by dividing it by the proportion of the dry weight using values from the literature[23,24] (Supplementary Table 10). For mite taxa, mean individual dry body mass were estimated based on a global dataset of soil fauna[25]: 5.3, 7.7 and 1 µg for Oribatida, Mesostigmata, and Prostigmata/Astigmata, respectively. Mite fresh body mass was calculated as described above assuming a dry matter content of 43.1%[26]. Collembola species were classified in four ecological groups (Epedaphic, Hemiedaphic, Euedaphic and Predators) based on Potapov *et al.* (2022[7], Supplementary Table 10). Microarthropods taxa were then assigned to seven trophic guilds[7]: Acari: Oribatida (microbi-detritivores), Acari: Mesostigmata (carnivores), Acari: Prostigmata & Astigmata (omnivores), Collembola: Epedaphic (fungivores), Collembola: Hemiedaphic (microbi-detritivores), Collembola: Euedaphic (fungivores), and Collembola: Predators (carnivores, Supplementary Table 5).

The abundances and biomasses of soil macroinvertebrates were measured as described by Ganault *et al.* (2021)[27]. All macroinvertebrate individuals were gently wiped and weighed to the nearest 0.01 mg to measure their fresh body mass. Since heavy soil materials can represent a significant fraction of faunal fresh mass for soil feeders, we corrected the fresh mass of endogeic and anecic earthworms by multiplying it with a conversion factor of 0.67 g empty gut fresh mass per g full gut fresh mass[25]. We converted macroinvertebrate fresh body mass (*M*) to dry body mass (*DM*) using the following equation[28]:

$$DM = e^{\frac{log(M) - a}{b}} \qquad (3)$$

where the a and b coefficient values are 0.9282 and 1.0899 for earthworms, and 0.6111 and 1.0213 for all other taxa, respectively. Lumbricidae species were classified in three ecological groups (Epigeic, Endogeic, and Anecic) based on the DriloBASE database (http://taxo.drilobase.org, Supplementary Table 11). Macroinvertebrates taxa were then assigned

to 23 trophic guilds[7]: Small Araneae (< 1.14 mg, carnivores), Large Araneae (> 1.14 mg, carnivores), Opiliones (carnivores), Diplopoda (detritivores), Chilopoda: Geophilomorpha (carnivores), Chilopoda: Scolopendromorpha (carnivores), Chilopoda: Lithobiomorpha (carnivores), Isopoda (detritivores), Dermaptera (omnivores), Blattodea (detritivores), Formicidae (omnivores), Coleoptera: Carabidae (carnivores), Coleoptera: Staphylinidae (carnivores), Coleoptera: Elateridae (omnivores), Coleoptera: Scarabaeidae/Geotrupidae (herbi-detritivores), Coleoptera: Curculionidae (herbivores), Lepidoptera (herbivores), Diptera (omnivores), Lumbricina: epigeic (detritivores), Lumbricina: anecic (humi-detritivores), Lumbricina: endogeic (humi-detritivores), Gastropoda: snails (detritivores), and Gastropoda: slugs (detritivores, Supplementary Table 5).

Calculation of metabolic rates and assimilation efficiencies

Plot-specific soil microbial respiration and biomass data from Gillespie *et al.* (2021)[29] was used to compute the metabolic rates of microbial groups. For heterotrophs, the metabolic rate is equal to the rate of respiration because heterotrophs obtain energy by oxidizing carbon compounds[30]. To measure soil microbial basal respiration, 10 g of fresh soil was incubated at 25°C and 80% of water holding capacity during 6 h, and CO_2 concentration was quantified after 2 and 6 h using a MicroGC (S-Series, SRA Intruments, Marcy l'Etoile, France). A similar incubation but including the addition of glucose (mass equivalent of 15 mg C) was used to estimate soil microbial biomass based on the substrate-induced respiration method using the following equation[31]:

$$C_{microbial} = MIRR \times 40.04 + 0.37 \quad (4)$$

where '$C_{microbial}$' is the active microbial biomass (µg $C_{microbial}$ g^{-1} dry soil) and '$MIRR$' is the substrate-induced maximum initial respiration rate (µl C-CO_2 g^{-1} dry soil $hour^{-1}$). The metabolic quotient of soil microbes (qCO_2, mg C-CO_2 g^{-1} $C_{microbial}$ $hour^{-1}$) was calculated as basal respiration divided by microbial biomass, and was standardized to the plot-specific mean soil temperature using the following equation[32]:

$$qCO_{2\,field} = qCO_{2\,incubation} \times Q_{10}^{\frac{T_{field} - T_{incubation}}{10}} \quad (5)$$

where '$qCO_{2\,field}$' and '$qCO_{2\,incubation}$' are the qCO_2 for field and incubation conditions, respectively, 'T_{field}' and '$T_{incubation}$' are the plot-specific mean soil temperature during the growing season (°C, see 'microclimate' section below) and the incubation temperature (25 °C), respectively. 'Q_{10}' is the temperature sensitivity coefficient, and a Q_{10} value of 2 was used[32]. Across all plots, we found $qCO_{2\,field}$ to be 1.90 ± 0.26 mg C-CO_2 g_{-1} $C_{microbial}$ h^{-1} (median ± standard deviation), which is close to the previously reported value of 1.81 based a previous meta-analysis[32].

Assuming qCO_2 to be two times lower for fungi than bacteria[33], the qCO_2 of fungi and bacteria were calculated using the following equations:

$$qCO_{2\,fungi} = \frac{qCO_{2\,field}}{2 \times f_{bacteria} + f_{fungi}} \quad (6)$$

$$qCO_{2\,bacteria} = 2 \times qCO_{2\,fungi} \quad (7)$$

where '$f_{bacteria}$' and 'f_{fungi}' are the bacterial and fungal fraction of total microbial biomass based on PLFA analysis, respectively. Based on this method, we found that bacterial respiration represent 16.8 ± 6.4 % (median ± standard deviation across all plots) of total soil microbial respiration, which is within the same range than previously reported values (12.2 ± 4.2 %) for soils within the pH range (~4.0) from a previous study using the selective inhibition method[34]. The metabolic rates (g C-CO_2 m^{-2} day^{-1}) of microbial trophic guilds were then calculated as their biomass multiplied by their metabolic quotient, and were converted to kJ m^{-2} day^{-1} using a conversion factor of 1 g C = 39 kJ based on the enthalpy of glucose combustion[35].

The metabolic rates of faunal groups were computed from individual body mass, environmental temperature and phylogenetic grouping using the following equation[30]:

$$I = e^{\ln i_0 + a \times \ln M - \frac{E}{kT}} \quad (8)$$

where I is the metabolic rate (in J hour^{-1}), i_0 is a normalization factor, a is the allometric exponent, M is the fresh body mass (in mg), E is the activation energy (eV), k is Boltzmann's constant (8.62 * 10^5 eV Kelvin^{-1}), and T is the environmental temperature (in Kelvin). Taxon-specific or otherwise general parameter values fitted from a large soil invertebrate respiration dataset[18] were used for i_0, a, and E (Supplementary Table 12). Plot-specific values of growing season soil temperature were used for T (see below). For macroinvertebrates, metabolic rates were calculated for each individual, and the community metabolism (X, in kJ m^{-2} day^{-1}) of each trophic guild was calculated as the sum of all individual metabolic rates divided by the sampled area. For nematodes and microarthropods, metabolic rates were calculated for each taxon using the mean individual fresh body mass, and the community metabolism of each trophic guild was calculated as the sum of the products of taxon-specific metabolic rate and taxon density.

Assimilation efficiencies (e_a) specific to each food type (plant-derived resource or prey) for faunal consumers were calculated based on resource stoichiometry using the following equation[36] (Supplementary Table 13):

$$e_a = \frac{\mathrm{e}^{(0.471\times\text{Food N}-2.097)}}{1+\mathrm{e}^{(0.471\times\text{Food N}-2.097)}} \quad (9)$$

where 'Food N' is the N content (%) of the food resource. For plant-derived resources, we used plot-specific N content measured as described above. For microbial and faunal trophic guilds, we used generic N content data available from the literature[7].

A temperature correction of assimilation efficiency (ε_{cor}) was calculated using the following equation[37]:

$$\varepsilon_{cor} = \frac{e^{\acute{\varepsilon}_{0,j}} e^{E_{\varepsilon,j}\frac{T-T_0}{kTT_0}}}{1+e^{\acute{\varepsilon}_{0,j}} e^{E_{\varepsilon,j}\frac{T-T_0}{kTT_0}}} - \varepsilon_{0,j} \quad (10)$$

where $\acute{\varepsilon}_0$ is the normalization constant of the assimilation efficiency, E_ε is the activation energy for assimilation efficiency (eV), T is the environmental temperature (K), T_0 is the temperature normalized to 20°C (293.15 K), k is the Boltzmann's constant (8.62 * 10^5 eV K^{-1}), and ε_0 is the assimilation efficiency at 20°C. The subscript j refers to the different consumer types (herbivores, detritivores, or carnivores). Consumer type-specific parameter values from the literature[37] were used for $\acute{\varepsilon}_0$, E_ε, and ε_0 (Supplementary Table 14). Plot-specific values of soil temperature during the growing season converted to Kelvin were used for T (see below). Assimilation efficiencies were then standardized to the plot-specific mean soil temperature during the growing season by adding the correction coefficient ε_{cor} to e_a.

Plant-derived resources

The biomass and N content of living plant fine roots and dead roots in each plot was measured as described by Wambsganss *et al.* (2021a, c)[38,39]. Briefly, roots were sampled during the phenological spring 2017 by taking one core of the soil layer (10 cm depth, 5.3 cm diameter) in each of the same five subplots used for soil sampling (Supplementary Fig. 5b). Living fine roots (absorptive roots belonging the first three root orders)[40] of all plants (dominated by woody tree species, but also with some herbaceous plants) were washed and visually sorted into dead and live roots, before being dried for at least 72 h at 40 °C and weighed to estimate the biomass of living plant fine roots and dead roots per unit surface area. Living plant fine roots were then pooled at the plot level, ground and their N content was analyzed by dry combustion using an elemental analyzer (EA, Elementar Vario El Cube). We assumed equal N content of living plant fine roots and root litter based on a global synthesis of plant root data[41].

The biomass and N content of the litter layer in each plot was measured as described by Gillespie *et al.* (2021)[29]. Briefly, four 15 × 15 cm square samples of the litter layer predominantly composed of dead leaves were sampled during phenological spring 2017 in each of the same five subplots used for soil sampling (Supplementary Fig. 5b). Litter material was then dried at 60°C, weighed to estimate the biomass of dead leaves per unit surface area, before being ground, and analyzed by dry combustion to mesure its N content.

The biomass and N content of dead wood in each plot was retrieved from the FunDivEurope database (https://data.botanik.uni-halle.de/fundiveurope/). Briefly, the volume per unit surface area of woody debris, that are all standing dead trees and snags, and all stumps and other dead wood pieces lying on the forest floor, was measured during summer 2012 in two circular subplots (radius of 7 m) located in opposite corners of each plot. The fresh volume and dry mass of a woody debris subsample were also measured to estimate wood density, and the biomass of dead wood per unit surface area was calculated as woody debris volume per unit surface area divided by wood density. The woody debris subsample was then ground, and its N content was analyzed by dry combustion.

The biomass and N content of soil organic matter (SOM) in each plot was measured as described by Dawud *et al.* (2016)[42]. Briefly, mineral soil (A horizon) was sampled during summer 2012 by taking one soil core (10 cm deep, 3.6 cm diameter) in each of nine subplots, and was then sieved through 2 mm and pooled at the plot-level, dried at 60°C for at least 48 h, ground and its C and N contents were analyzed by dry combustion. The C stock of SOM was calculated based on soil C content and bulk density, and SOM dry mass was estimated assuming a C content of SOM of 50%.

Reconstruction of the soil food web topology and interaction strengths

To reconstruct the food web interaction matrix representing the topology and trophic interaction strengths of the food web, a set of five matrices with food resources in rows and consumers in columns were first generated, and then merged by multiplying them together (see Fig. 5 in Potapov 2022[43] for an illustration). Each matrix represents one trait dimension: phylogenetically defined feeding preferences, density(biomass)-dependence, predator–prey interactions related to body mass ratio and hunting strategies, prey protection mechanisms, and spatial niche overlap related to vertical stratification. To generate the matrices, we used multiple parameters mostly derived from Potapov *et al.* (2022)[7] corresponding to organism traits (Supplementary Table 5). Food web reconstruction was carried out separately for each plot to account for plot-specific density(biomass)-dependence.

First, we generated a resource matrix based on phylogenetically inherited differences in feeding preferences of consumers for various food resources[7], including living plant fine roots roots (LivR), photosynthates & rhizodeposits (PR), dead leaves (LLit), dead roots (RLit), dead wood (WLit), soil organic matter (SOM), bacteria (Ba), mycorrhizal fungi (FuM), general saprotroph fungi (FuS), wood saprotroph fungi (FuW), plant pathogenic fungi (FuP), and animal prey (Fauna, Supplementary Table 5). For each trophic guild, feeding preferences of the corresponding consumers for different food resources (plant-derived resources and/or other consumers) were determined assuming that additional food resources (coded as 0.5) are five times less important than the main resources (coded as 1)[43]. For omnivores, *i.e.* organisms that have developed predation capabilities while also feeding on other food resources (both coded as 1), animal diet was assumed to represent 50% of the node budget[44]. Feeding preferences were then scaled so that preferences of a given consumer for all food resources summed to 1. Feeding preferences were defined based on Potapov *et al.* (2022)[7] for faunal trophic guilds, while feeding preferences of microbial trophic guilds were defined based on previous isotopic labeling studies[45]. Specifically, photosynthates and rhizodeposits were defined as the primary food resources for mycorrhizal fungi[46,47] and gram-negative bacteria[48–50]. Decomposer fungi were defined to feed primarily on litter[51–53] and rhizodeposits[49,53], but also secondarily on soil organic matter[53,54]. Gram-positive bacteria were defined to feed primarily on soil organic matter[48,51] and litter[51,52].

Second, we generated a biomass matrix corresponding to density dependent preferences of consumers for various food resources by filling each row with the biomass of the corresponding trophic guild.

Third, we generated an allometric matrix to modulate the strength of carnivore trophic interactions based on predator–prey mass ratio[55] (PPMR) and predator traits. The optimum

PPMR was first calculated for each faunal predator based on their mean body mass (in g, log_{10}-transformed values given in Supplementary Table 5 as 'Mass mean') using the following equation[55]:

$$log_{10}PPMR_{optimum} = -0.159 \times log_{10}\text{body mass} + 0.330 \quad (11)$$

For each faunal predator, the mean body mass of its optimum prey was then calculated by subtracting $PPMR_{optimum}$ from the predator mean body mass, while the body mass standard deviation of its optimum prey was calculated by multiplying $PPMR_{width}$ ('Mass SD' in Supplementary Table 5) with predator mean body mass. For most predators, the body mass standard deviation of its optimum prey was set to be the same as the body mass standard deviation of the predator ($PPMR_{width}$ = 1). However, the values of $PPMR_{optimum}$ and $PPMR_{width}$ was first corrected according to hunting strategies by multiplying them with the correction coefficients based on predator traits (Supplementary Table 5). The distributions of optimum and real prey body mass were then modelled using the mean and standard deviation of optimum and real body mass, assuming these distributions to follow a log-normal pattern. Overlaps of the size distributions of optimum and real prey were then calculated for all potential predator–prey interactions, and overlap proportion [0;1] was used as a proxy of interaction strength.

Fourth, we generated a protection matrix to modulate the strength of carnivore trophic interactions that they can be considerably reduced by prey protective traits. An empty matrix was filled with ones, and it was then multiplied by each of the correction coefficients based on protective traits (Supplementary Table 5). The lower the coefficient, the higher the protection.

Fifth, a spatial matrix was generated to modulate the strength of trophic interactions based on consumer-resource overlap in habitat niches. Pairwise Bray–Curtis dissimilarities for all nodes were calculated based on the vertical distribution of each node in soil (euedaphic, 'eu'), litter (hemiedaphic, 'hemi'), or surface (epiedaphic, 'epi'). Vertical distribution was coded as 0 (nearly absent), 0.5 (occasional) and 1 (present, Supplementary Table 5). Note that a given node can occur in multiple vertical layers, owing to organism mobility for instance. Reverse dissimilarity [0;1], as a measure of spatial overlap, was used as a proxy for interaction strength.

Finally, the five matrices were combined to generate the food web interaction matrix. All the matrices, except the resource matrix, were first multiplied together. The resulting matrix was then multiplied by the resource matrix separately for basal, microbial and faunal types of food resources, and then scaled so that trophic guild preferences for all food resources of a given type summed to the proportion of this given resource type in the trophic guild diet. This approach allows to avoid the overestimation of the contribution of basal and/or microbial resources to the diet due to their much larger biomass[43]. The full R script used to generate the trophic interaction matrix is accessible in the figshare repository, available at https://doi.org/10.6084/m9.figshare.31700881.

Calculation of soil food web energy fluxes, trophic functions and multifunctionality

Energy fluxes to each feeding guild of the food web were calculated using the following equation[56]:

$$F = \frac{1}{e_a} \times (X + L) \quad (12)$$

where F (kJ m^{-2} day^{-1}) is the total flux of energy into the feeding guild, e_a is the diet-specific assimilation efficiency, X is the community metabolism of the feeding guild, and L is the energy loss to predation.

More specifically, energy fluxes of individual trophic interactions were calculated using the following equation[57]:

$$\sum_j F_i W_{ji} e_{a\,ji} = X_i + \sum_j F_j W_{ij} \quad (13)$$

where F_i and F_j are the sum of all ingoing fluxes to feeding guild i and j, respectively, '$e_{a\,ji}$' is the diet-specific assimilation efficiency of the food resource j consumed by feeding guild i, X_i is the energy lost through community metabolism of the feeding guild i, and W_{ji} and W_{ij} are the proportions of F_i and F_j that are obtained from feeding guilds j and i, respectively. This equation

is solved in two stages: first, the sum of ingoing fluxes for each trophic guild (F_i) is computed. Second, individual fluxes for each pairwise consumer-food resource interaction (F_{ji}) are calculated using consumer preferences (W_{ji}) based on the trophic interaction matrix. The calculation of energy fluxes starts with the top predators and proceeds downward to the trophic guilds with the lowest trophic position. The loss to predation in these lower feeding guilds was synonymous with energy fluxes directed toward their consumer feeding guilds. Energy fluxes were computed using the '*fluxing*' function of the '*fluxweb*' package[57].

Defining high multifunctionality by fast rates of multiple functions simultaneously[58], we quantified soil food web multifunctionality using the 'averaging' approach[59] by estimating the average standardized values of 10 trophic functions of the soil food web: mycorrhizal symbiotrophy, root pathogenicity, rhizophagy, litter decomposition, litter engineering, SOM decomposition, soil engineering, bacterivory, fungivory and carnivory. All of the ten trophic functions were first standardized using the following equation[59]:

$$F_i = \frac{rawF_i - \min(rawF)}{\max(rawF) - \min(rawF)} \quad (14)$$

where F_i and $rawF_i$ are the standardized and unstandardized function values of plot i, respectively, and min($rawF$), and max($rawF$) are the minimal and maximal values of the function across all plots, respectively. The averaged multifunctionality index was then calculated as the mean of the 10 standardized trophic functions. To check that the results were robust to multiple alternative approaches, we also quantified soil food web multifunctionality using the 'threshold' approach[59] by estimating the number of trophic functions whose value exceeded 30, 50, 70 and 90% of the maximal value. Calculations for the 'threshold' approach were performed using the '*getFuncMaxed*' function of the '*multifunc*' package[60]. For both approaches, the maximum value of the trophic function was calculated as the mean of the four highest values to reduce the influence of outliers[59].

Tree functional (trait-based) diversity and composition

Leaf trait data for each tree species in each plot was mostly derived from *in situ* measured values retrieved from the FunDivEurope database (https://data.botanik.uni-halle.de/fundiveurope/). Leaf nitrogen content (LNC) was measured for each tree species in all plots, while specific leaf area (SLA), and leaf dry matter content (LDMC) were measured for each tree species in Finland and Romania plots only. The three leaf traits were measured on sun-exposed leaves of each tree species collected in spring 2015 using standard protocols[61]. Briefly, ten individual trees of each target species present were selected in each plot, and five leaves were sampled for each of these individual trees. Each sampled leaf was gently wiped after overnight immersion in tap water, weighed to measure leaf fresh mass, and scanned with a flat-bed scanner (resolution 800 dpi). Scans were then analysed with the WinFOLIA software (Regents Instruments, Québec, Canada, 2009) to obtain leaf area. Subsequently, each leaf was dried for at least 72 h at 40 °C, and weighed to measure leaf mass. SLA and LDMC were then calculated as leaf area divided by dry mass, and leaf fresh mass divided by dry mass, respectively. Leaf samples of each tree were then pooled, ground and their N content was analysed by dry combustion. Trait values were averaged at the plot level (one value per tree species and plot). For Italy and Poland, SLA and LDMC values of each of the seven tree species of these two countries were retrieved using the TRY plant trait database[62] (https://www.try-db.org/, accession date October 2019, request n° 6303), and we then used the average of trait values for each individual trait and tree species.

Root trait data was derived from *in situ* values measured in each plot[39]. Briefly, the six root traits were measured on tree fine roots (absorptive roots belonging to the first three root orders[40]) of each species using standard protocols as described by Wambsganss *et al.* (2021)[39]. Root tips colonised by ectomycorrhizal fungi (EcM) were first visually identified and counted on representative subsamples based on the presence or absence of a fungal sheath for the 12 species known to associate with EcM (*Acer pseudoplatanus* occurring in Romania associate with arbuscular mycorrhizal fungi). Thereafter, roots were scanned in water with a flat-bed scanner

(resolution 800 dpi), and scans were analysed with the WinRhizo software (Regents Instruments, Québec, Canada, 2009) to obtain root length, volume and diameter. Subsequently, all root samples were dried for at least 72 h at 40 °C and weighed to obtain root dry mass. Samples were then ground, and their N content was analysed as described above to obtain root N content (RNC). Root tissue density (RTD) was calculated as root dry mass divided by root volume. Specific root length (SRL) was calculated as root length divided by root dry mass. Measured root diameter values were averaged to obtain mean root diameter (D_m). Root length density (RLD) was calculated as the length of all fine roots recovered in the sampled soil core divided by the soil core volume. Ectomycorrhizal colonisation intensity (%Myc) was calculated as the number of root tips colonised by ectomycorrhizal fungi (EcM) divided the length of roots examined.

For each plot, we quantified tree functional diversity based on these nine traits using the functional dispersion (FDis) index, which is the mean distance of each species to the centroid of all species in the multifunctional trait space[63]. For monospecific stands, the computation of the FDis index always yielded a value equal to zero because of the absence of functional dispersion when only one tree species is present. We also calculated community-weighted mean (CWM) of each trait to characterize tree functional composition[64]. FDis and CWM values were computed based on the relative basal area of tree species using the '*dbFD*' function of the '*FD*' package[63]. We also performed a Principal Component Analysis (PCA) on all CWM traits using the '*principal*' function of the '*psych*' package[65]. This allowed to simplify the tree functional composition into two dimensions[39,66] (Extended Data Fig. 4a): (1) a leaf economics spectrum (LES; 45.2 % of variation), which ranged from slow/conservative leaf attributes (N-poor and dry matter-rich leaves with low leaf area per unit mass) to fast/acquisitive leaf attributes (N-rich and dry matter-poor leaves with high leaf area per unit mass), and which was also aligned here with a fine-root gradient of belowground resource foraging strategies ranging from low foraging efficiency (thick fine roots with low length per unit mass) to high foraging efficiency (thin fine roots with high length per unit mass); (2) a fine-root economics spectrum (RES; 31.5 % of variation), which ranged from slow/conservative fine-root attributes (N-poor fine roots with low tissue density) to fast/acquisitive fine-root attributes (N-rich fine roots with high tissue density), and which was also aligned here with a fine-root gradient of soil exploration strategies ranging from 'do-it-yourself' attributes (high root length density and low ectomycorrhizal colonisation intensity) to 'outsourcing' attributes (low root length density and high ectomycorrhizal colonisation intensity). The LES axis was positively indeed related to LNC, SLA and SRL, and negatively to LDMC and D_m, while the RES axis was positively related to RNC and %Myc, and negatively to RTD and RLD (Supplementary Table 7).

Tree and understorey vegetation

For each plot, all tree stems ≥ 7.5 cm in diameter at breast height (DBH) were identified to species, and their height and DBH were measured in spring 2017 for Finland, Romania and Italy, and in spring 2018 for Poland. Height and DBH measurements were used to estimate tree aboveground biomass (AGB) based on published allometric equations[67], as well as the basal area of each tree. Aboveground litter production of tree communities was quantified by regularly collecting canopy tree litterfall (including leaf, wood and reproductive parts) over one entire year from October 2011 to November 2012 using five litter traps (0.5 m^2, 1 m above the ground) per plot[68]. Collected litter was pooled by plot, sorted by species and litter type (*i.e.* leaves, twigs, and reproductive parts), and weighed after drying at 65 °C. The percentage cover of understorey plant species (< 1.3 m tall individuals) was characterized in three 5 m × 5 m quadrats in each plot between May and August 2012[69]. At the same time, the aboveground biomass and C:N ratio of woody and herbaceous vegetation were determined from samples cut in a smaller area (0.5 m × 0.5 m) within each of these three quadrats. Understorey plant diversity was quantified using the species richness calculated at the plot-level (combined across the three sampling quadrats). Total root biomass was quantified as the sum of absorptive (first three orders) and transport (higher orders) roots of both herbaceous and woody plant species[38].

Environmental drivers

Abiotic conditions

In each plot, altitude was recorded during plot selection. Plot-specific mean annual temperature and precipitation were retrieved using the WorldClim database[70] (https://www.worldclim.org/). The texture of mineral soil texture was measured in each plot using the laser diffraction method[71].

We performed a PCA as described above to reduce the dimensionality of abiotic properties into two dimensions[39,72] (Supplementary Fig. 2a): (1) a soil texture gradient ranging from coarse to fine, coordinated with a macroclimate gradient ranging from dry and cold to wet and hot (PC1; 70.8% of variation), (2) a gradient of temperature and altitude ranging from cold and high elevation to warm and low elevation (PC2; 19.1% of variation).

Microclimate

Microclimate was measured in the center of each plot at all four countries during an entire year from spring 2018 to spring 2019. Soil temperature and moisture (0-14 cm soil depth), and air temperature (50 cm above the ground) were measured every 15 min using TMS-4 data loggers (TOMST, Prague, Czech Republic)[73], with a special version allowing temperature measurements 50 cm above the ground. Specifically, the bottom part of the TMS logger constitutes a probe measuring volumetric soil moisture from the soil surface until a depth of 14 cm, and also includes a soil temperature probe located in the middle at 8 cm depth. Mean annual values and mean growing season (daily mean air temperatures >5 °C) values were calculated for soil and air temperature, and soil moisture. Daily minimum values instead of means were used in the calculation for soil moisture. We did this because water tends to accumulate around the sensors after rain events, thus leading to an overestimation of soil moisture when using all values measured per day[73].

The microclimatic dataset was incomplete due to missing values following wild boar damage on TMS-4 data loggers in six plots (five in Poland, and one in Italy). These missing values were replaced based on a regularized iterative multiple correspondence analysis[74] using the '*imputePCA*' function of the '*missMDA*' package[75].

We performed a PCA as described above to reduce the dimensionality of microclimatic properties into a single dimension (PC1; 63.6% of variation; Extended Data Fig. 4b). This microclimate axis ranged from 'cold and wet' to 'hot and dry', and was positively related to soil and air temperature, and negatively to soil moisture, both for annual and growing season values.

Leaf litter quality

Litter properties of each tree species were measured for each location using standard protocols as described by Joly *et al.* (2017)[68] on freshly fallen leaf litter of each species. Litter material was collected at tree species-specific peak leaf litter fall between October 2011 and November 2012, in close vicinity of the experimental plots, and was processed as described above (see 'Tree and understorey vegetation' section). Briefly, total C and N concentrations were measured with a flash CHN Elemental Analyser (Flash EA1112 Series, ThermoFinnigan, Milan, Italy). After a mineralization step, phosphorus (P) concentration was measured colorimetrically with an autoanalyzer (SmartChem 200, Alliance Instruments, Rome, Italy). Water soluble compounds (WSC), cellulose, hemicellulose and lignin fractions were determined using a fiber analyzer (Fibersac 24, Ankom, Macedon, NJ, USA) according to the Van Soest extraction protocol[76]. Calcium (Ca) concentration was measured using an atom absorption spectrometer (AAS, iCE 3000 series, Thermo-Scientific, China). Concentrations of condensed tannins were measured spectrophotometrically using the butanol-HCl method[77]. For total phenolics, samples of 0.5 g were soaked in 30 mL of 50% methanol, shaken for 2 h, filtered (filter number 112, Durieux, Torcy, France), and the extracts were analyzed spectrophotometrically with the FolineCiocalteau reagent by using gallic acid as a standard. Leaf litter pH was measured according to Cornelissen *et al.* (2006)[78]. Water holding capacity (WHC) was measured by placing intact leaves in non-hermetically lidded large plastic containers and spraying them three times a day with deionized

water. The amount of water retained by the leaves was measured after 48 h. Except for pH and physical traits, which were measured on intact leaf litter, all other chemical analyses were performed on subsamples ground to obtain a uniform particle size of 1 mm (Cyclotec Sample Mill, Tecator, Höganäs, Sweden).

We then quantified the functional diversity and composition of tree leaf litter for each plot using the FDis index and CWMs of each litter property as described above for tree functional traits, except that the computation was based on the relative leaf litter mass of the different tree species collected in litter traps[68], rather than the relative basal area of trees. We also performed a PCA similar to that described above for tree functional traits to reduce the dimensionality of litter CWM properties into one dimension: a spectrum of leaf litter nutritional qualities[79] (PC1; 42.5% of variation%; Extended Data Fig. 4c). The leaf litter quality axis ranged from low to high nutritional quality, and was related positively to WHC and the concentrations of N, P, K, Mg, WSC, hemicellulose, total phenolics and soluble phenolics, and negatively to the C:N ratio and concentrations of lignin and cellulose.

Soil fertility

For each plot, soil properties were measured using standard protocols on forest floor (including both unfragmented aboveground litter and fragmented/humified organic matter, OL/OF/OH horizons) and mineral soil (A horizon, 10 cm depth) samples (n = 9 samples per plot) collected between spring and summer 2012 as described by Dawud *et al.* (2016)[42]. Briefly, the forest floors was sampled using a 25 cm by 25 cm wooden frame, while mineral soil was sampled with a soil corer (3.6 cm diameter). The nine samples were then homogenized and pooled at the plot level, dried at 55 °C to constant weight, and forest floor dry mass per unit surface area was measured. The pH was determined using a 827 pH lab sensor (Metrohm AG, Herisau, Switzerland) with 0.01 M $CaCl_2$ solution at a ratio of 1:10 and 1:2.5 for forest floor and mineral soil, respectively. Both forest floor and mineral soil were then ground, and their C and N contents were measured by dry combustion using a FLASH 2000 Soil CN Analyzer (Thermo Fisher Scientific, Milan, Italy). The soil fertility dataset had missing values for forest floor pH and C:N in one Finland plot. These missing values were replaced based on a regularized iterative multiple correspondence analysis[74] as described above.

We performed a PCA as described above to reduce the dimensionality of soil properties (including both forest floor and mineral soil) into a single dimension (PC1; 34.8% of variation; Supplementary Fig. 2b). This soil fertility axis ranged from low to high fertility, and was related positively to forest floor pH and lignin concentrations, as well as mineral soil pH, SOC, silt and clay contents, and negatively to forest floor C:N ratio, total phenolics and condensed tannins concentrations, as well as mineral soil C:N ratio.

Measured ecosystem processes

To characterize *in situ* patterns of litter decomposition in each plot, we used field-based data[80] of naturally occurring leaf litter decomposition. Briefly, litterbags were filled with 10 g of air-dried leaf litter consisting of a litter mixture with equal proportions of litter from all tree species present in the plot. Freshly senesced leaf litter from each tree species was collected at tree species-specific peak leaf litter fall between October 2011 and November 2012, in close vicinity of the experimental plots. Three replicate litterbags filled with the plot-specific leaf litter were placed on the bare soil, after the natural litter layer had been locally removed, within a 1 m^2 homogeneous area of each plot. Litterbags were retrieved when the most rapidly decomposing species within each region reached 40-50% mass loss. Collected decomposed materials were dried at 65 °C, cleaned of pieces of wood, stones or other foreign material that occasionally got into the litterbags and weighed. To correct for potential soil contamination during decomposition in the field, litter samples were ground with a Cyclotec Sample Mill (Tecator), their ash content determined and their mass loss rates expressed based on ash-free litter mass. To account for the differences in the duration of field exposure among the different locations, litter decomposition

rate (mg g^{-1} litter day^{-1}) was calculated as the ratio of mass lost per amount of initial mass per day of incubation. Litter decomposition per unit surface area (mg m^{-2} day^{-1}) was calculated as the litter decomposition rates multiplied by the biomass of the litter layer.

Soil organic carbon (SOC) mineralization per unit surface area was estimated based on plot-specific soil microbial respiration and biomass data[29] decribed above to characterize SOM decomposition. SOC mineralization per unit surface area (mg C-CO_2 m^{-2} day^{-1}) was calculated as the metabolic quotient of soil microbes standardized to the plot-specific soil temperature ($qCO_{2\,field}$; mg C-CO_2 g^{-1} $C_{microbial}$ day^{-1}) and multiplied by the soil microbial biomass per unit surface area ($C_{microbial}$; g $C_{microbial}$ m^{-2}).

Statistical analyses

Data analysis was performed using R Statistical Software (v4.3.0)[81] with the Rstudio interface (2023.03.1+446). The following packages were used: readxl (v1.4.3), xlsx (v0.6.5), erer (v3.1), purrr (v1.0.1), Matrix (v1.5-4.1), dplyr (v1.1.2), FactoMineR (1.34), fluxweb (v0.2.0)[57], vegan (v2.6-8)[82], FD (v1.0-12.1)[83], missMDA (1.11)[75], rstanarm (v2.21.4)[84], loo (v2.6.0)[85], bridgesampling (v1.1-2)[86], performance (v0.12.3)[87], bayesplot (v1.10.0)[88], piecewiseSEM (v2.3.0)[89], lme4 (1.1-33)[90], and multifunc (v0.9.4)[60]. All R scripts for statistical analyses are accessible in the figshare repository, available at https://doi.org/10.6084/m9.figshare.31700881.

Bayesian linear mixed-effects models (LMMs) were fitted based on Markov Chain Monte Carlo (Hamiltonian Monte Carlo algorithm) methods using the '*rstanarm*' package[84]. We relied on the default settings of the '*rstanarm*' package for the priors, which are intended to be weakly informative, providing moderate regularization and helping stabilize computation (https://cran.r-project.org/web/packages/rstanarm/vignettes/priors.html#default-weakly-informative-prior-distributions). Five chains with different starting values were each ran over 2,000 iterations of both warm-up and sampling phases, resulting in a posterior sample size of 10,000. The target average acceptance probability ('adapt_delta') was set to 0.9999 to prevent divergent transitions and ensure reliable inference. We tested for model convergence by examining trace plots and evaluating the R-hat (*i.e.* the ratio of between-chain variance to within-chain variance) and N_{eff} (*i.e.* the ratio of the effective sample size to the overall number of iterations) statistics. All models were graphically checked for assumptions of normality of residuals, homogeneity of variances, linearity of relationships, and normality of random effects. We also performed graphical posterior prediction checks simulating replicated data under the fitted model and then comparing these to the observed data to ensure the lack of discrepancies between real and simulated data[91]. For multiple regressions, multicollinearity issues were also checked based on variance inflation factors with a threshold value of 3[92] using the '*check_collinearity*' function of the '*performance*' package[87], and collinear variables were omitted from the model when necessary. We observed low levels of multicollinearity for both the first analytical step testing the effects of tree diversity and composition on soil food web functioning (Supplementary Table 15), and the second analytical step testing pathways of tree community effects on soil food web functioning through structural equation modelling (Supplementary Table 3). Graphical checks of the four model assumptions, posterior prediction, an multicollinearity issues were performed using the '*check_model*' function of the '*performance*' package[87]. We also checked for independence of residuals (*i.e.* autocorrelation of error terms) based on the Durbin-Watson-Test using the '*check_autocorrelation*' function of the '*performance*' package[87]. To account for outliers and improve normality of the residuals, all response variables were log-transformed, except for 'soil engineering', 'detritivore biomass', and 'humi-detritivore biomass' which were log(x+1)-transformed[93].

To perform variance partitioning, we adopted a model-based approach based on variance components following Schulz *et al.* (2025)[94]. Our Bayesian modelling approach allowed to avoid singular fit issues, which are common when dealing with complex models with few random-factor levels, and can bias variance partitioning[94,95]. We quantified the proportion of variance in the dependent variable explained by three groups of predictors: diversity (tree species richness

or FDis), composition (tree species composition or CWM trait PCs 1 and 2), and biogeography (abiotic condition PCs 1 and 2, as well as location), along with the model residuals (ε). In this framework, a variance component refers to the variance associated with a given model component (a_j), which can either be fixed ($x_j\beta_j$) or random (δ_j or ε). Each model component is assigned to a single predictor groups (m), so the group-level effect can be formally expressed as $b_l = \sum_{j\in B_l} a_j$ where $l = 1, \ldots, m$ and B_l is a set of model components associated with the lth group. The variance of a group-level model component is then $Var[b_l] = Var\left[\sum_{j\in B_l} a_j\right] = \sum_{j\in B_l}\sum_{j'\in B_l} Cov\left[a_j, a'_j\right]$. Using this, we computed the diagonal variance partitioning for each group as:

$$V_{b_l}^{diag} = \frac{Var[b_l]}{\sum_{j'=1}^{m} Var[b_{l'}]} \tag{15}$$

This so-called *diagonal variance partition* ensures that the different group-level variance components sum up to one. However, it also ignores the covariances in the denominator, and hence also does not indicate the presence of confounding related to covariance between group-level model components. To explore the effects of covariance between group-level variance components[94] (so that the variance of the dependent variable may not equate the sum of group-level variance components), we also computed *basic variance partition* (V_b), *i.e.* the variance of a group of model components standardized by the variance of the dependent variable (*y*), using the following equation:

$$V_{b_l} = \frac{Var[b_l]}{Var[y]} \tag{16}$$

We also computed the *marginal variance partition* (M_b), which measure the contribution of a group of model components while accounting for covariances with all other groups of model components, using the following equation:

$$M_{b_l} = \frac{\sum_{j'=1}^{m} Cov[b_l, b_{l'}]}{Var[y]} \tag{17}$$

Finally, we computed the *partial variance partition* (P_b), which measure the contribution of a group of model components that cannot be accounted for by a linear combination of the other groups of model components, using the following equation:

$$V_{b_l} = \frac{Var\left[\hat{\eta}\left(b_l - \hat{b}_l(a_{\setminus l})\right)\right]}{Var[y]} \tag{18}$$

where $b_{\setminus l}$ collects all other groups of model components except b_l, and the hat-notation denotes linear least squares predictor, so that for example, $\hat{\eta}_j\left(b_{\setminus l}\right)$is the linear least squares prediction for η using $b_{\setminus l}$; as $\eta = \sum_{l=1}^{m} b_l$, it holds that $\hat{\eta}(b_1, \ldots, b_l) = \eta$.

To calculate regression slopes standardized by the standard deviation (β_{st}), raw slopes (β_{unst}) were scaled by the ratio of the standard deviation (sd) of the predictor variable (x) over the standard deviation of the response variable (y) using the following equation[96]:

$$\beta_{st} = \beta_{unst} \times \frac{sd_x}{sd_y} \tag{19}$$

Standardized slope (β_{st}) values of $|\beta_{st}| < 0.12$, $0.12 \leq |\beta_{st}| < 0.24$, $0.24 \leq |\beta_{st}| < 0.41$, and $|\beta_{st}| \geq 0.41$ were interpreted as neutral, weak, moderate, and strong effects, respectively[97].

Bayes factors (BFs) were calculated as the ratio of marginal likelihoods of two models: an alternative model (m_1) including the random factor tested over a null model (m_0) not including the random factor. A value of BF > 1 indicates that m_1 is more strongly supported by the data than m_0. The marginal likelihood of each model was calculated using the '*bridge_sampler*' function of the '*bridgesampling*' package[86]. We computed predicted values of the dependent variables for the average values of predictors and for different levels of the tree species richness predictor using the '*posterior_linpred*' function of the '*rstanarm*' package[84].

For each component LMM of the initial SEM model (Supplementary Table 8), we performed stepwise backward model selection based on Pareto smoothed importance-sampling leave-one-out (PSIS-LOO) cross-validation[98] using the '*loo*' function of the '*loo*' package[85]. We fitted the

full component LMM, computed the p-values associated with each fixed factor[99], and then removed the term with the highest p-value. To check that it improved model goodness-of-fit, the nested component LMMs were compared based on the LOO information criterion, *i.e.* the expected log predictive density (ELPD) multiplied by -2, with lower values indicating better model goodness-of-fit. We started by performing model selection on the component LMM predicting soil food web multifunctionality, and then performed selection on component LMMs predicting each of the variables retained as predictors of soil food web multifunctionality. To account for outliers in the SEM model, the variables 'tree aboveground biomass', 'tree aboveground litterfall', 'total root biomass', 'understory plant aboveground C:N' and 'soil food web multifunctionality' were log-transformed, while 'understory plant aboveground biomass (herbaceous)' was log(x+1)-transformed.

The SEM model goodness-of-fit was assessed by the Shipley's directional separation test[100] combining the p-values of conditional independence claims in a test statistic, *i.e.* the Fisher's C, using the following equation:

$$C = -2\sum_{i=1}^{k} \ln(p_i) \quad (20)$$

where p_i is the ith conditional independence claim in a basis set consisting of k conditional independence claims associated with the hypothesized causal diagram. Fisher's C can then be compared to a χ^2 distribution with $2k$ degrees of freedom. The hypothesized relationships of the SEM model are considered to be consistent with the data when there is weak support for the sum of the conditional independence claims, *i.e.* where the collection of such missing relationships represented by the Fisher's C could have easily occurred by chance, in which case the p-value for the χ^2 test is greater than the chosen significance threshold (here α = 0.05)[89]. For some conditional independence claims that were not verified (missing relationships unlikely to have occurred by chance), we fitted these relationships as correlated errors as these variables were assumed not to be causaly linked (*i.e.* to be caused by a shared underlying, unmeasured driver).

Sensitivity analyses

To ensure that our inferences were robust to various sources of uncertainty and bias, we performed several sensitivity analyses. First, we explored whether the effects of tree communities on soil food web multifunctionality were consistent for multiple alternative approaches to quantify multifunctionality. The 'averaging' approach adopted here provides a straightforward and easily interpretable measure of multifunctionality but it also has some limitations, such as its inability to distinguish between multiple functions performing at intermediate values from some performing at high values while others perform at low values due to trade-offs among functions[59]. To check that our inferences were robust to multiple alternative approaches, we also calculated soil food web multifunctionality using the 'threshold' approach[59] by estimating the number of trophic functions whose value exceeded 10, 20, 30, 40, 50, 60, 70, 80, and 90% of the maximal value (Supplementary Methods). We found that the two methods provided fairly consistent results for the effects of tree community properties with both the taxonomic and functional approaches (Supplementary Table 16).

Second, we addressed the potential uncertainty arising from the use of SLA and LDMC traits data retrieved from the TRY database for tree species in Poland and Italy, which may have limitations relative to on-site measurements because of intraspecific trait variation[101]. To assess how this might have affected our inferences, we performed 1,000 simulations. In each simulation, we fitted a Bayesian LMM (as described in main text equation 2) using functional metrics (FDis and the first two principal components of CWM traits) computed from trait data in which SLA and LDMC values for the plots in Poland and Italy were randomly varied by around 30% around their original values retrieved from the TRY database. This 30% value was chosen because the coefficients of variation of the trait values retrieved from the TRY database for each tree species present in Poland and Italy were on average 25.3 % for SLA and 28.4% for LDMC. For each simulation, we extracted the standardized partial slopes and p-values of each variable, and

visually checked their distributions. We found that this uncertainty in trait values had minimal effect on our inferences regarding soil food web multifunctionality (Supplementary Fig. 6).

Third, we checked that our inferences were also valid when including a random slope in the models. The use of random-slope models (with varying intercepts and slopes across locations for each predictor) instead of random-intercept models has been recommended to control for inflated Type I (false positive) error[102,103], but such a confidence improvement comes at the cost of a significant loss of statistical power[104] (1 - Type II [false negative] error), *i.e.* a reduced ability to detect a particular effect that is actually present. We therefore performed the same analyses using random-slope models (following equations 1 and 2 in the main text). Both approaches provided very similar results for effect size though their uncertainty was higher when using random-slope models, and the inclusion of random slopes never substantially improved model goodness-of-fit (Supplementary Tables 1 and 17). To avoid model overfitting, we only present and discuss results from the simplest approach based on random-intercept models in the main text.

Fourth, we checked whether omitted variable bias due to potential unmeasured confounding variables at the location-level could affect our inference[105]. In ecological studies, linear mixed-effects model (LMMs) are commonly used to account for unmeasured cluster-level variables by incorporating them into random effects. However, this approach can lead to biased causal inference when random effects are correlated with the causal variable of interest. This issue is particularly relevant for variables not directly controlled by the study design, as is the case in our functional approach. To address this, we applied a correlated random effects approach ('Mundlak' approach), which incorporates group-level means of the causal variables to control for unmeasured confounders at the cluster (location) level. Including these group means serves as a proxy for potential confounding variables. This allowed us to estimate contextual effects, *i.e.* the between-location effects, of the causal variable. If the contextual effect is close to zero, it suggests that a standard LMM is sufficient and that omitted variable bias is likely minimal. For each causal variable used in the functional approach, we therefore fitted a Bayesian LMM (similar to the one described in main text equation 2), that additionally included the location-level mean of the causal variable ($\bar{x}_i$). For all three causal variables, we found that the effect estimates remained consistent, and that the contextual effects ($\beta_{contextual}$) were close to zero (Supplementary Table 18). This indicates that omitted variable bias was not a concern in our study, given that we also accounted for measured abiotic confounding variables.

Supplementary Results

Relationships between calculated soil food web functions and measured soil functions

To test the theoretical assumption that the computed trophic functions based on aggregated energy fluxes through the soil food web provides a good description of independently measured ecosystem functions, we performed simple regressions or analysis of variance where a given measured ecosystem function was predicted based on Bayesian linear models including the corresponding calculated trophic function as fixed factor. Overall, we found that this theoretical assumption was well supported (Extended Data Fig. 3). Indeed, the calculated decomposition of litter and soil organic matter (consumption of litter or soil organic matter by microbes) per unit surface area were positively related to measured litter mass loss and soil organic matter mineralization per unit surface area, respectively (Extended Data Fig. 3a,e). Similarly, the calculated litter engineering rate (consumption of litter by fauna per unit litter mass) was positively related to measured litter decomposition rate (litter mass loss per unit litter mass, Extended Data Fig. 3b), reflecting the stimulation of litter decomposition rate by soil faunal detritivory[106]. We also found that the calculated soil engineering (consumption of soil organic matter by fauna) per unit surface area was much higher for Mull/Amphimull humus types, characterized by the homogenization of humified organic matter with mineral particles within macro-aggregates, than for Moder/Mor humus types, characterized by the presence of humified organic matter (OH) separated from the mineral soil[107,108] (Extended Data Fig. 3c). This shows

that the calculated soil engineering is well representing the bioturbation of mineral soil by burrowing fauna such as endogeic and anecic earthworms. Accordingly, the calculated soil engineering per unit surface area was negatively related to the measured mass of forest floor (both unfragmented aboveground litter and fragmented/humified organic matter, OL/OF/OH, Extended Data Fig. 3d), reflecting the rapid disappearance of litter and humified organic matter from the surface due to the bioturbation and translocation of dead organic matter from the surface to mineral soil by anecic earthworms. Altogether, these results provide empirical evidence for good agreement between calculated trophic functions and measured soil processes, confirming that our quantification of aggregated energy fluxes in soil food webs are reflective of soil functioning[109,110].

Exploration of the effects of correlation between variance components
We found that correlations between variance components were low for the taxonomic approach. As a result, the variance components standardized by the variance in soil food web multifunctionality, *i.e.* basic variance partition (V_b), were very similar to alternative variance partition metrics (Extended Data Table 2). These included the marginal variance partition (M_b), which quantifies the contribution of a group of model components while accounting for covariances with other groups, and the partial variance component (P_b), which quantifies the unique contribution of a group of model components not explained by a linear combination of the others. The low covariance observed between biogeography and species composition, despite notable species turnover across locations (Extended Data Table 1), can be attributed to the linear mixed-effects model's ability to account for nesting, *i.e.* species composition was largely nested within locations in our study design[111].

In contrast, in the functional approach, we observed a strong correlation between the variance components of functional composition and biogeography (Extended Data Table 2). This is because functional composition metrics were modeled as fixed effects, while location was included as a random effect. Consequently, the basic variance partitions (V_b) for functional composition and biogeography were both large (78.91 and 43.77 %, respectively), while their partial variance partitions (P_b) were much lower (16.22 and 18.17%, respectively), indicating partial confounding between these two components[94]. Moreover, the marginal variance partition (M_b) for functional composition remained substantial (31.87 %), but was nearly zero for biogeography. This suggests that biogeography had a minimal direct effect on soil food web multifunctionality, but a substantial indirect effect mediated through its influence on community-level economic trait variation across geographic locations, which was driven by both species turnover and intraspecific trait variability.

Supplementary Figures and Tables

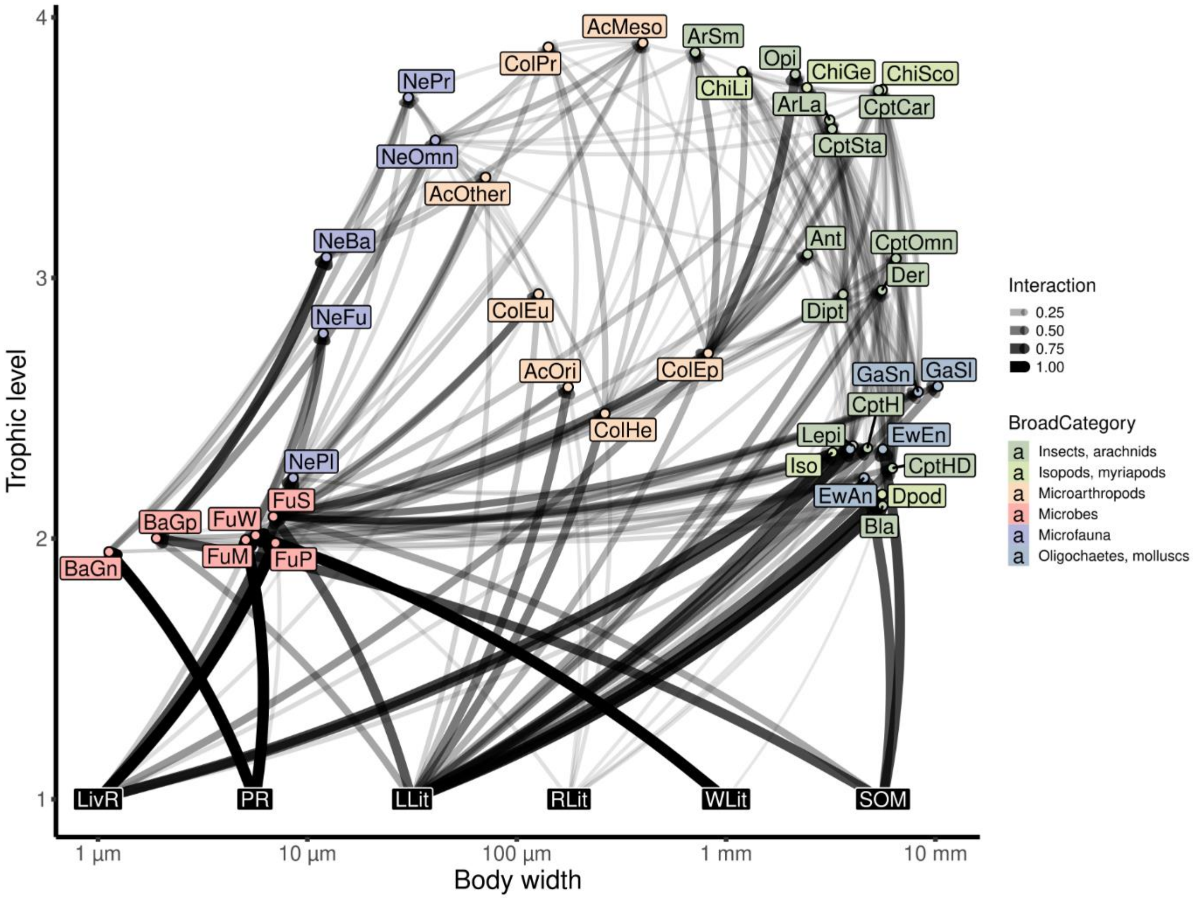

Supplementary Fig. 1. Graphical illustration of the food web topology and trophic interaction strengths among trophic guilds for the studied European forest plots. Arrows indicate energy flows among trophic groups, with arrow widths corresponding to trophic interaction strengths. The position of the trophic guilds are based on their body width and trophic level within the food web. The horizontal position of basal ressources at the food web bottom is arbitrary. Biomass data averaged across all plots was used to calculate trophic interaction strengths shown in this illustration. LivR, Living plant fine roots; PR, Photosynthates; LLit, Dead leaves; RLit, Dead roots; WLit, Dead wood; SOM, Soil organic matter; BaGn, Bacteria: Gram-; BaGp: Gram+; FuM, Mycorrhizal fungi; FuS, General saprotroph fungi; FuW, Wood saprotroph fungi; FuP, Plant pathogenic fungi; NePl, Nematoda: Plant feeders; NeBa, Nematoda: Bacterivores; NeFu, Nematoda: Fungivores; NeOmn, Nematoda: Omnivores; NePr, Nematoda: Predators; AcOri, Acari: Oribatida; AcMeso, Acari: Mesostigmata; AcOther, Acari: Prostigmata & Astigmata; ColEp, Collembola: Epedaphic; ColHe, Collembola: Hemiedaphic; ColEu, Collembola: Euedaphic; ColPr, Collembola: Predators; ArSm, Araneae: Small; ArLa, Araneae: Large; Opi, Opiliones; Dpod, Diplopoda; ChiGe, Chilopoda: Geophilomorpha; ChiSco, Chilopoda: Scolopendromorpha; ChiLi, Chilopoda: Lithobiomorpha; Iso, Isopoda; Der, Dermaptera; Bla, Blattodea; Ant, Formicidae; CptCar, Coleoptera: Carabidae; CptSta, Coleoptera: Staphylinidae; CptOmn, Coleoptera: Omnivores; CptHD, Coleoptera: Herbidetritivores; CptH, Coleoptera: Root feeders; Lepi, Lepidoptera; Dipt, Diptera; EwEp, Lumbricina: Epigeic; EwAn, Lumbricina: Anecic; EwEn, Lumbricina: Endogeic; GaSn, Gastropoda: Snails; GaSl, Gastropoda: Slugs. The interaction strength values shown here are provided in Supplementary Table 19. The illustration is adapted from Potapov (2022)[43].

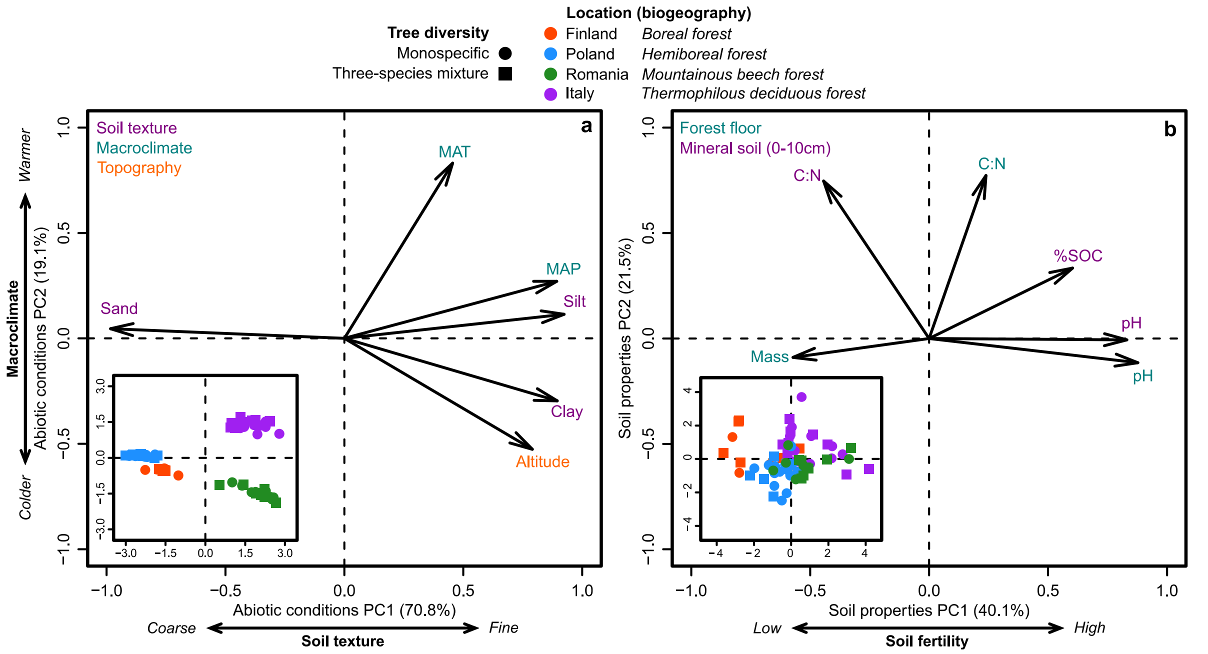

Supplementary Fig. 2. Ordination by Principal Component Analysis (PCA) of abiotic conditions (a), and soil properties (b). Variable loadings indicate the correlation of the variables with the corresponding principal components. Insets show plot coordinates (*n* = 64 plots). MAT, mean annual temperature; MAP, mean annual precipitation; %SOC, soil organic carbon content.

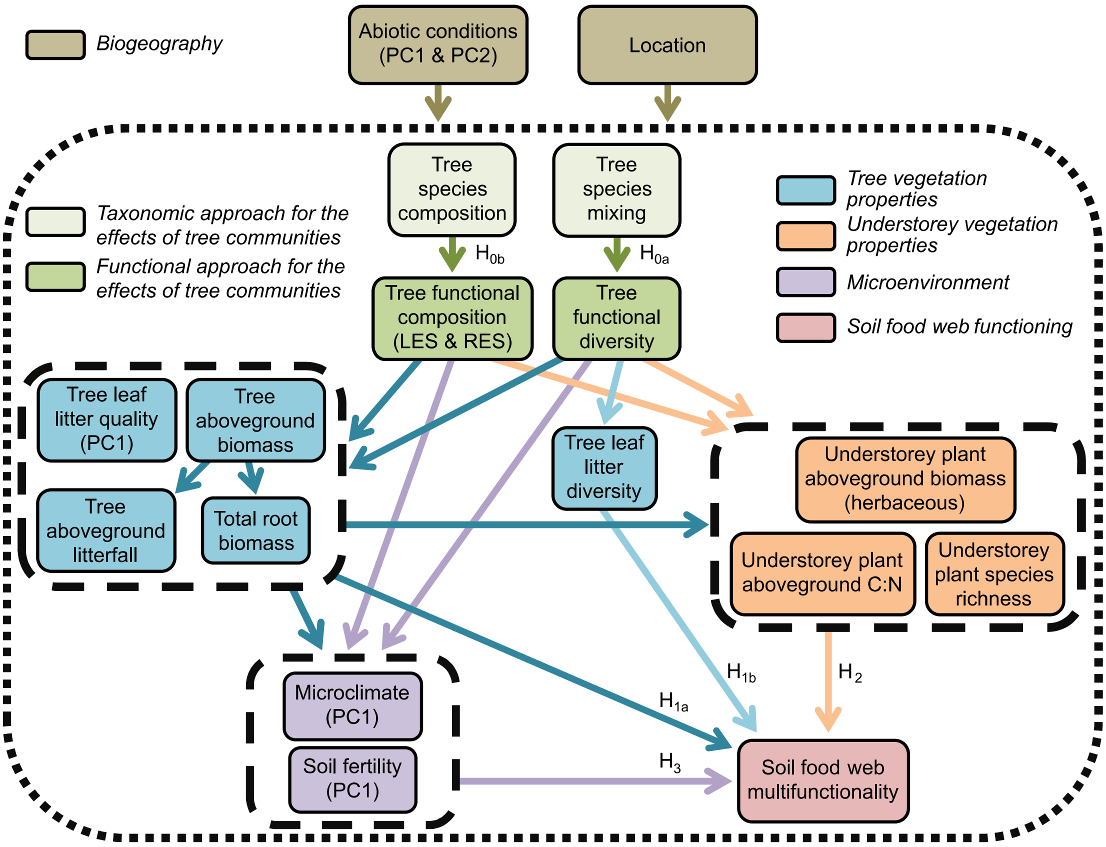

Supplementary Fig. 3. Causal diagram illustrating *a priori* hypotheses about how tree communities influence soil food web multifunctionality. Arrows indicate hypothesized causal relationships. LES, leaf economics spectrum; RES, fine-root economics spectrum. Rounded rectangles with a dotted outlines denote sets of variables that are jointly influenced by other variables (arrows in) or jointly influence others (arrows out). The diagram was constructed based on ecological theory and prior knowledge of the system. We hypothesized that tree species mixing influences soil food web multifunctionality through complementarity mechanisms dependent on trait variation among coexisting species[112–115], *i.e.* the 'functional diversity' hypothesis[116] (**H$_{0a}$**), and that tree species composition acts through community-weighted mean trait values, *i.e.* the 'mass-ratio' hypothesis[115,117] (**H$_{0b}$**). We further hypothesized that both tree diversity and composition influence soil food web multifunctionality through changes in tree vegetation properties (**H$_{1a}$**). For example, plant functional composition is known to determine litter quality[79], an important driver of soil food web functioning[118,119]. Tree diversity has also been shown to increase aboveground biomass[67,120], which may in turn affect soil food webs[121,122]. Tree diversity and composition, along with aboveground biomass, can in turn affect aboveground litterfall[68] and root biomass[38], both of which provide important trophic resource for soil organisms[123,124]. We also hypothesized that tree diversity affects soil food web multifunctionality by altering tree leaf litter diversity[125–127] (**H$_{1b}$**). Furthermore, we hypothesized that effects of tree diversity and composition on soil food web multifunctionality are mediated by changes in understorey vegetation properties (**H$_2$**), which may themselves be influenced by tree vegetation properties. Relevant understorey vegetation properties potentially controlling soil food web functioning include understory plant biomass (particularly for herbaceous species)[128], litter quality[129] (here approximated by the aboveground C:N ratio), and species richness[122] (used here as a proxy for plant diversity). Finally, we hypothesized that tree diversity and composition influence soil food web multifunctionality through modifications of the micro-environment (**H$_3$**), potentially mediated by tree community properties, for example through effect on soil properties [130,131], or forest microclimates[132–134], which can in turn affect soil food web functioning[30,109,135].

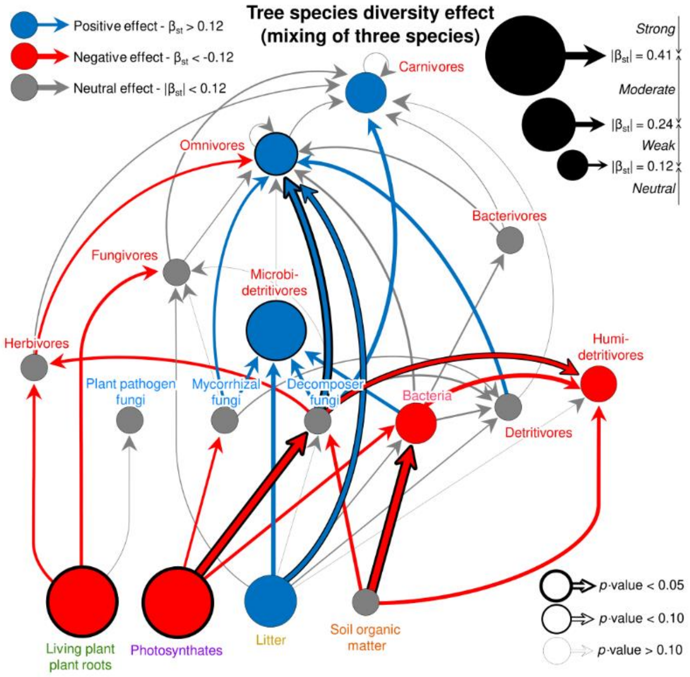

Supplementary Fig. 4. Effects of tree species mixing on energy fluxes and trophic group biomasses of the soil food web across European forests. Circle areas and arrow widths are scaled by the posterior mean effect size of tree species richness (posterior *n* = 10,000). Effect size was quantified using partial slope regression coefficients standardized by the standard deviation (β_{st}), derived from Bayesian linear mixed-effects models used for the taxonomic approach (equation 1, *n* = 64 plots, see Methods in the main text). The *p*-values were derived from posterior distributions using a two-tailed test.

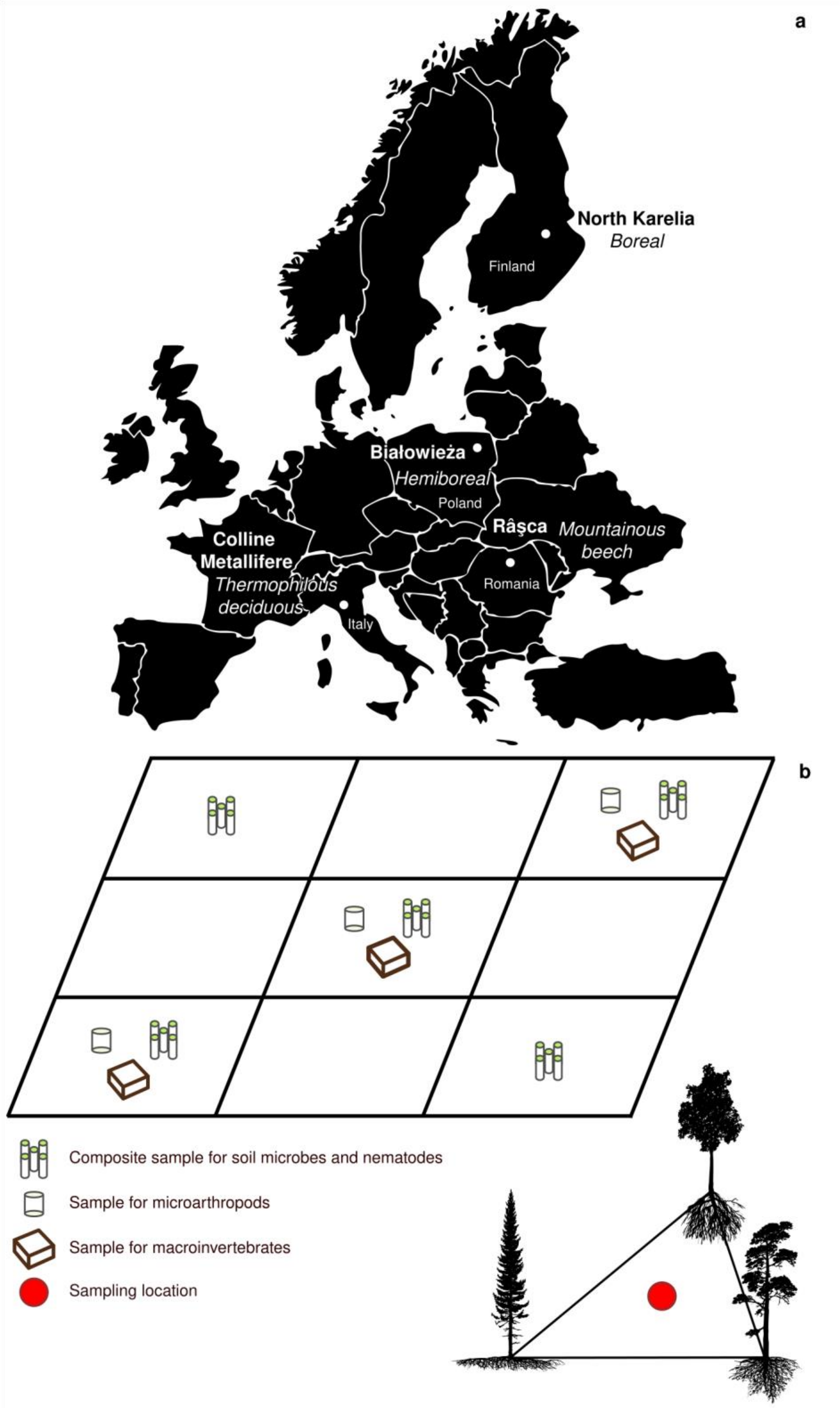

Supplementary Fig. 5. Map of study site location across Europe (a), and illustration of the sampling design for each plot (b).

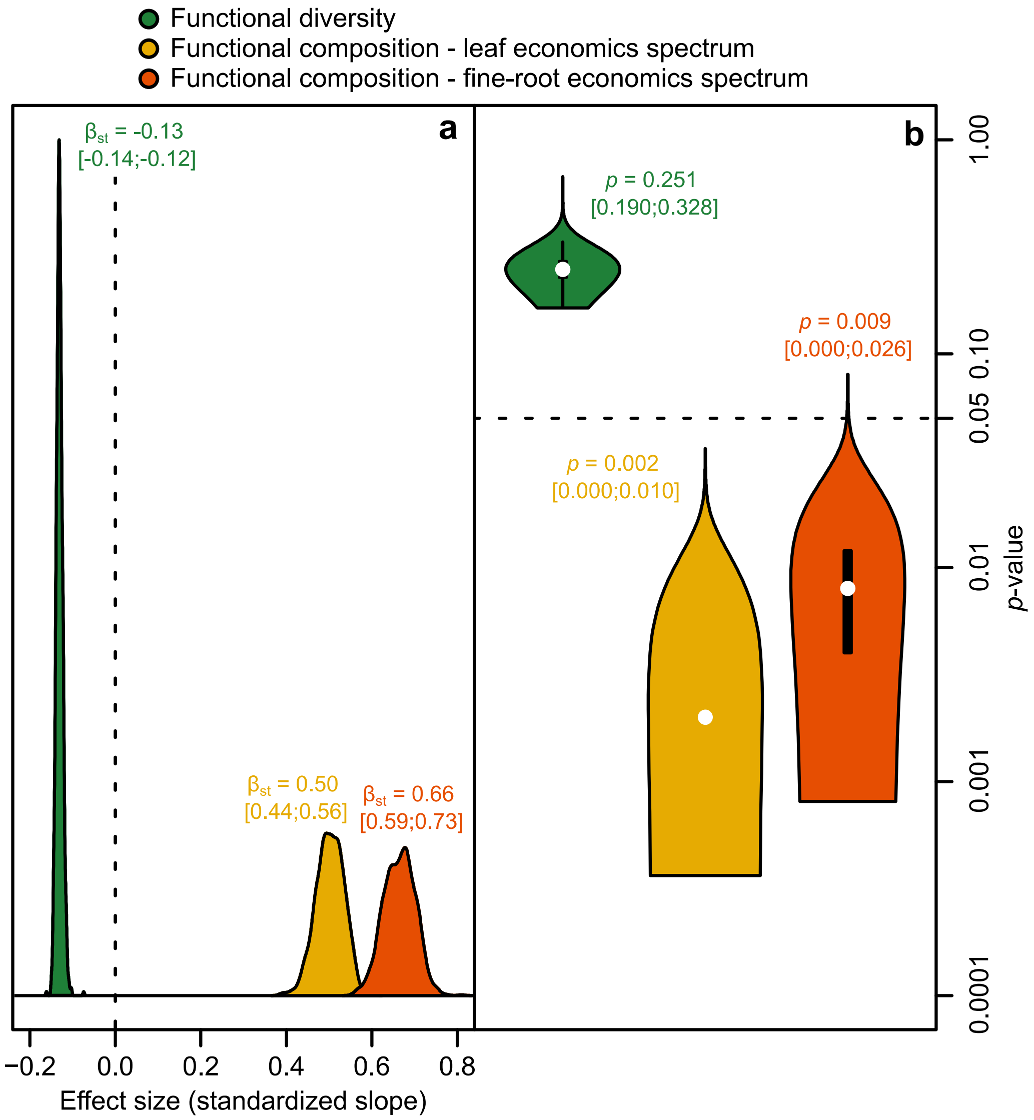

Supplementary Fig. 6. Sensitivity analysis of uncertainty arising from the use of SLA and LDMC trait data retrieved from the TRY database for tree species in Poland and Italy. **a**, density plot of effect sizes (partial slopes standardized by the standard deviation); **b**, violin plot of *p*-values. These results were based on 1,000 simulations in which Bayesian linear mixed-effects models (as described in equation 2 in the main text) were fitted using functional metrics (FDis and the first two principal components of CWM traits) computed from trait data where SLA and LDMC values for plots in Poland and Italy were randomly varied by ±30% around their original TRY database values in each simulation. Text values indicate the mean and 95% credible interval (in brackets) of posterior mean values across the simulation sample. White dots indicate median values. Thick black lines indicate the interquartile ranges (IQR), *i.e.* the difference between the third and first quartiles. Thin black lines indicate 1.5 × IQR.

Supplementary Table 1. Results of Bayesian multi-level models (equations 2 and 3; *n* = 64 plots; see Methods in the main text) testing tree community effects on trophic functions and trophic group biomasses of the soil food web, alongside aboveground tree biomass. Both the leaf and fine-root economics spectra (LES & RES) variables representing tree functional composition ranged from slow/conservative to fast/acquisitive attributes (Extended Data Fig. 4a). % var, posterior mean proportion of summed variance components explained (posterior n = 10,000). β_{st}, posterior mean of slope regression coefficients standardised by standard deviation. CI 95%, 95% posterior credible interval. BF, Bayes factor (see Methods). The p-values were derived from posterior distributions using a two-tailed test. Significant effects ($p < 0.05$ or BF > 1) are reported in bold. †, $p < 0.10$; *, $p < 0.050$; **, $p < 0.010$; ***, $p < 0.001$. SOM, soil organic matter.

	Taxonomic approach for tree community effects						Functional approach for tree community effects								
	Three-species mixing			Species composition		r^2	Functional diversity			Functional composition					r^2
											Leaf economics spectrum		Fine-root economics spectrum		
	% var	β_{st} [CI 95%]	*p*-value	% var	BF		% var	β_{st} [CI 95%]	*p*-value	% var	β_{st} [CI 95%]	*p*-value	β_{st} [CI 95%]	*p*-value	
**Soil food web functioning**															
Carnivory	1.23	-0.00 [-0.24;0.22]	0.973	7.43	0.62	0.49	0.83	0.02 [-0.18;0.21]	0.857	39.23	0.38 [0.11;0.66]	**0.007****	0.58 [0.14;1.07]	**0.008****	0.52
Microbivory	1.47	-0.04 [-0.29;0.20]	0.717	12.76	**1.39**	0.54	0.63	-0.03 [-0.23;0.17]	0.754	38.05	0.30 [0.02;0.58]	**0.039***	0.78 [0.30;1.23]	**0.004****	0.50
Fungivory	1.26	-0.01 [-0.25;0.22]	0.916	7.46	0.54	0.47	0.59	0.01 [-0.20;0.22]	0.911	24.69	0.24 [-0.08;0.53]	0.137	0.67 [0.12;1.15]	**0.020***	0.46
Bacterivory	1.63	-0.09 [-0.28;0.09]	0.305	4.57	0.57	0.67	1.76	-0.11 [-0.26;0.06]	0.194	28.33	0.21 [-0.01;0.43]	0.066†	0.46 [0.10;0.82]	**0.015***	0.68
Detritivory	3.34	-0.15 [-0.41;0.11]	0.252	8.72	0.54	0.33	2.75	-0.15 [-0.39;0.08]	0.196	17.57	0.23 [-0.08;0.54]	0.147	0.37 [-0.12;0.90]	0.132	0.31
Soil engineering	4.10	-0.18 [-0.43;0.07]	0.135	4.74	0.36	0.35	3.09	-0.17 [-0.41;0.05]	0.130	14.18	0.33 [0.02;0.62]	**0.032***	0.17 [-0.34;0.63]	0.470	0.39
SOM decomposition	5.14	-0.27 [-0.55;0.00]	0.054†	21.28	**3.03**	0.46	5.14	-0.24 [-0.47;-0.00]	**0.048***	10.79	0.24 [-0.08;0.59]	0.140	0.13 [-0.38;0.76]	0.676	0.31
Litter engineering	2.99	0.13 [-0.13;0.40]	0.328	14.00	**1.37**	0.47	2.32	0.14 [-0.07;0.36]	0.197	23.85	0.30 [-0.03;0.62]	0.069†	0.46 [-0.07;1.00]	0.085†	0.41
Litter decomposition	1.52	-0.04 [-0.30;0.21]	0.745	4.50	0.35	0.28	1.33	-0.06 [-0.29;0.17]	0.624	21.89	0.15 [-0.18;0.47]	0.344	0.46 [-0.10;0.98]	0.094†	0.31
Herbivory	4.01	-0.18 [-0.42;0.06]	0.140	12.38	**1.43**	0.55	1.88	-0.14 [-0.34;0.06]	0.178	17.95	0.40 [0.07;0.71]	**0.018***	0.28 [-0.31;0.78]	0.327	0.51
Rhizophagy	3.03	-0.15 [-0.37;0.08]	0.185	7.37	0.71	0.54	1.95	-0.15 [-0.35;0.05]	0.132	16.43	0.19 [-0.14;0.49]	0.237	0.46 [-0.13;0.98]	0.120	0.50
Root pathogenicity	1.76	-0.03 [-0.31;0.25]	0.836	23.00	**5.43**	0.51	0.77	0.02 [-0.20;0.23]	0.884	21.10	0.49 [0.20;0.78]	**0.001****	0.25 [-0.19;0.74]	0.269	0.44
Plant C allocation to soil by roots	2.95	-0.13 [-0.37;0.10]	0.259	9.35	0.75	0.50	1.42	-0.10 [-0.31;0.11]	0.357	10.30	0.18 [-0.17;0.53]	0.320	0.28 [-0.33;0.91]	0.362	0.46
Soil food web multifunctionality	4.43	-0.19 [-0.46;0.10]	0.192	22.26	**4.24**	0.47	1.57	-0.13 [-0.35;0.10]	0.254	36.67	0.49 [0.20;0.80]	**0.002****	0.68 [0.20;1.17]	**0.009****	0.38

Supplementary Table 1. (Continued).

	Taxonomic approach for tree community effects						Functional approach for tree community effects								
	Three-species mixing			Species composition		r^2	Functional diversity			Functional composition					r^2
											Leaf economics spectrum		Fine-root economics spectrum		
	% var	β_{st} [CI 95%]	*p*-value	% var	BF		% var	β_{st} [CI 95%]	*p*-value	% var	β_{st} [CI 95%]	*p*-value	β_{st} [CI 95%]	*p*-value	
**Trophic group biomasses**															
Carnivores	4.73	0.18 [-0.08;0.45]	0.186	9.88	0.60	0.29	6.08	0.26 [0.02;0.49]	**0.029***	17.67	0.36 [0.01;0.69]	**0.046***	0.06 [-0.61;0.58]	0.790	0.33
Omnivores	4.21	0.18 [-0.02;0.40]	0.073†	5.52	0.57	0.56	3.71	0.17 [-0.02;0.35]	0.080†	12.71	-0.13 [-0.40;0.12]	0.304	0.22 [-0.23;0.61]	0.292	0.57
Fungivores	1.74	0.07 [-0.17;0.31]	0.568	4.28	0.34	0.37	1.25	0.09 [-0.12;0.30]	0.410	18.17	0.05 [-0.25;0.34]	0.747	0.52 [0.03;0.98]	**0.040***	0.41
Bacterivores	0.93	0.02 [-0.17;0.21]	0.832	3.62	0.43	0.61	0.80	-0.01 [-0.19;0.17]	0.919	10.99	0.01 [-0.24;0.25]	0.903	0.24 [-0.17;0.62]	0.222	0.61
Microbi-detritivores	7.57	0.26 [-0.00;0.52]	0.051†	26.40	**16.98**	0.61	5.19	0.23 [0.02;0.44]	**0.028***	11.21	-0.25 [-0.53;0.05]	0.103	-0.05 [-0.52;0.51]	0.792	0.46
Humi-detritivores	3.54	-0.16 [-0.40;0.07]	0.169	5.49	0.41	0.41	2.55	-0.16 [-0.37;0.05]	0.141	15.26	0.36 [0.06;0.64]	**0.017***	0.19 [-0.28;0.64]	0.387	0.45
Detritivores	2.07	0.08 [-0.17;0.33]	0.493	12.58	**1.14**	0.51	1.54	0.10 [-0.11;0.30]	0.366	17.04	0.37 [0.06;0.68]	**0.023***	0.11 [-0.44;0.62]	0.667	0.48
Herbivores	2.19	0.08 [-0.20;0.36]	0.576	10.14	0.59	0.24	1.25	0.02 [-0.23;0.28]	0.858	12.62	0.01 [-0.41;0.39]	0.943	-0.27 [-1.01;0.35]	0.445	0.19
Plant pathogenic fungi	1.60	0.00 [-0.28;0.27]	0.968	19.23	**3.16**	0.52	0.84	0.04 [-0.17;0.24]	0.715	18.90	0.44 [0.16;0.74]	**0.002****	0.23 [-0.21;0.73]	0.310	0.45
Decomposer fungi	1.65	-0.05 [-0.31;0.20]	0.681	6.60	0.38	0.32	1.36	-0.05 [-0.29;0.18]	0.650	10.12	0.17 [-0.14;0.49]	0.274	0.14 [-0.36;0.68]	0.567	0.29
Mycorrhizal fungi	1.96	-0.09 [-0.31;0.13]	0.429	7.52	0.62	0.53	0.93	-0.06 [-0.26;0.14]	0.576	9.36	0.13 [-0.20;0.48]	0.439	0.27 [-0.33;0.88]	0.376	0.50
Bacteria	4.16	-0.18 [-0.42;0.05]	0.115	8.70	0.75	0.32	2.89	-0.16 [-0.38;0.05]	0.131	12.69	-0.01 [-0.33;0.33]	0.911	0.33 [-0.19;0.95]	0.230	0.45
Soil organic matter	1.49	-0.05 [-0.28;0.19]	0.691	5.24	0.37	0.39	1.14	-0.01 [-0.24;0.21]	0.901	11.82	-0.14 [-0.44;0.21]	0.563	-0.22 [-0.69;0.34]	0.365	0.38
Plant litter	6.50	0.23 [-0.08;0.54]	0.131	38.09	**36.09**	0.54	3.48	0.19 [-0.04;0.42]	0.097†	24.15	-0.28 [-0.63; 0.05]	0.091†	0.44 [-0.21;0.97]	0.160	0.36
Living plant fine roots/Photosynthates	10.28	-0.30 [-0.54;-0.05]	**0.019***	19.04	**2.89**	0.61	7.55	-0.28 [-0.49;-0.09]	**0.008****	18.57	-0.14 [-0.46;0.18]	0.401	-0.38 [-0.92;0.20]	0.180	0.48
Aboveground tree biomass	2.99	0.14 [-0.07; 0.33]	0.174	12.10	**1.87**	0.70	2.85	0.16 [-0.01;0.33]	0.070†	10.61	-0.04 [-0.32;0.24]	0.780	0.23 [-0.26;0.70]	0.341	0.62

Supplementary Table 2. Basis set of models testing all conditional independence claims implied by the final SEM model (*n* = 64 plots; see Methods in the main text). abiotic.PC1/abiotic.PC2, abiotic conditions (PC1/PC2), SR, tree species richness; compo, tree species composition ;fundiv, tree functional diversity; LES, tree functional composition (leaf economics spectrum); RES, tree functional composition (fine-root economics spectrum); AGB, tree aboveground biomass; litterfall, tree aboveground litterfall; litter.quality, tree leaf litter quality (PC1); root, total root biomass; microclim, microclimate (PC1); herbaceous, understorey plant aboveground biomass (herbaceous); multifun, soil food web multifunctionality. The *p*-values were derived from posterior distributions using a two-tailed test (posterior *n* = 10,000).

Conditional independance claim	Model formula	H_0	*p*-value
(SR, AGB) | {fundiv, RES}	AGB~SR+fundiv+RES+abiotic.PC1+(1|location)	SR = 0	0.749
(SR, litter.quality) | {LES}	litter.quality~SR+LES+(1|location)	SR = 0	0.619
(SR, root) | {fundiv, AGB}	root~SR+fundiv+AGB+(1|location)	SR = 0	0.584
(SR, microclim) | {fundiv, LES, RES, abiotic.PC1, abiotic.PC2}	microclim~SR+fundiv+LES+RES+abiotic.PC1+abiotic.PC2+(1|location)	SR = 0	0.572
(SR, herbaceous) | {AGB, root}	herbaceous~SR+AGB+root+(1|location)	SR = 0	0.736
(SR, multifun) | {AGB, litterfall, litter.quality, root, herbaceous, microclim}	multifun~SR+AGB+litterfall+litter.quality+root +herbaceous+microclim+(1|location)	SR = 0	0.989
(LES, fundiv) | {SR}	fundiv~LES+SR+(1|location)	LES = 0	0.123
(LES, AGB) | {fundiv, RES}	AGB~LES+fundiv+RES+abiotic.PC1+(1|location)	LES = 0	0.821
(LES, litterfall) | {fundiv, AGB}	litterfall~LES+fundiv +AGB+(1|location)	LES = 0	0.091
(LES, root) | {fundiv, AGB}	root~LES +fundiv+AGB+(1|location)	LES = 0	0.338
(LES, herbaceous) | {AGB, root}	herbaceous~LES+AGB+root+(1|location)	LES = 0	0.634
(LES, multifun) | {AGB, litterfall, litter.quality, root, herbaceous, microclim}	multifun~LES+AGB+litterfall+litter.quality+root +herbaceous+microclim+(1|location)	LES = 0	0.745
(RES, fundiv) | {SR}	fundiv ~RES+SR+(1|location)	RES = 0	0.133
(RES, litterfall) | {fundiv, AGB}	litterfall~RES+fundiv +AGB+(1|location)	RES = 0	0.103
(RES, litter.quality) | {LES}	litter.quality~RES+LES+(1|location)	RES = 0	0.540
(RES, root) | {fundiv, AGB}	root~RES+fundiv+AGB+(1|location)	RES = 0	0.267
(RES, herbaceous) | {AGB, root}	herbaceous~RES+AGB+root+(1|location)	RES = 0	0.829
(RES, multifun) | {AGB, litterfall, litter.quality, root, herbaceous, microclim}	multifun~RES+AGB+litterfall+litter.quality+root +herbaceous+microclim+(1|location)	RES = 0	0.323
(abiotic.PC1, fundiv) | {SR}	fundiv~abiotic.PC1+SR+(1|location)	abiotic.PC1 = 0	0.751
(abiotic.PC1, LES) | {compo}	LES~abiotic.PC1+(1|compo)+(1|location)	abiotic.PC1 = 0	0.764
(abiotic.PC1, RES) | {compo}	RES~abiotic.PC1+(1|compo)+(1|location)	abiotic.PC1 = 0	0.917
(abiotic.PC1, litterfall) | {fundiv, AGB}	litterfall~abiotic.PC1+fundiv+AGB+(1|location)	abiotic.PC1 = 0	0.633
(abiotic.PC1, litter.quality) | {fundiv, LES}	litter.quality~abiotic.PC1+LES+(1|location)	abiotic.PC1 = 0	0.651
(abiotic.PC1, root) | {fundiv, AGB}	root~abiotic.PC1+fundiv+AGB+(1|location)	abiotic.PC1 = 0	0.339
(abiotic.PC1, herbaceous) | {AGB, root}	herbaceous ~abiotic.PC1+AGB+root+(1|location)	abiotic.PC1 = 0	0.253
(abiotic.PC1, multifun) | {AGB, litterfall, litter.quality, root, herbaceous, microclim}	multifun~ abiotic.PC1+AGB+litterfall+litter.quality+root +herbaceous+microclim+(1|location)	abiotic.PC1 = 0	0.761
(abiotic.PC2, fundiv) | {SR}	fundiv~abiotic.PC2+SR+(1|location)	abiotic.PC2 = 0	0.993
(abiotic.PC2, LES) | {compo}	LES~abiotic.PC2+(1|compo)+(1|location)	abiotic.PC2 = 0	0.230
(abiotic.PC2, RES) | {compo}	RES~abiotic.PC2+(1|compo)+(1|location)	abiotic.PC2 = 0	0.073
(abiotic.PC2, AGB) | {fundiv, RES}	AGB~abiotic.PC2+fundiv+RES+abiotic.PC1+(1|location)	abiotic.PC2 = 0	0.810
(abiotic.PC2, litterfall) | {fundiv, AGB}	litterfall~abiotic.PC2+fundiv+AGB+(1|location)	abiotic.PC2 = 0	0.075
(abiotic.PC2, litter.quality) | {fundiv, LES}	litter.quality~abiotic.PC2+LES+(1|location)	abiotic.PC2 = 0	0.711

Supplementary Table 2. (Continued).

Conditional independance claim	Model formula	H_0	*p*-value
(abiotic.PC2, root) | {fundiv, AGB}	root~abiotic.PC2+fundiv+AGB+(1|location)	abiotic.PC2 = 0	0.739
(abiotic.PC2, herbaceous) | {AGB, root}	herbaceous ~abiotic.PC2+AGB+root+(1|location)	abiotic.PC2 = 0	0.767
(abiotic.PC2, multifun) | {AGB, litterfall, litter.quality, root, herbaceous, microclim}	multifun~ abiotic.PC2+AGB+litterfall+litter.quality+root +herbaceous+microclim+(1|location)	abiotic.PC2 = 0	0.920
(fundiv, litter.quality) | {SR, LES}	litter.quality~fundiv+SR+LES+(1|location)	fundiv = 0	0.455
(fundiv, herbaceous) | {SR, AGB, root}	herbaceous~fundiv+SR+AGB+root+(1|location)	fundiv = 0	0.343
(fundiv, multifun) | {SR, AGB, litterfall, litter.quality, root, herbaceous, microclim}	multifun~fundiv+SR+AGB+litterfall+litter.quality+root +herbaceous+microclim+(1|location)	fundiv = 0	0.638
(AGB, microclim) | {fundiv, LES, RES, abiotic.PC1, abiotic.PC2 }	microclim~AGB+fundiv+LES +RES+abiotic.PC1 +abiotic.PC2 +(1|location)	AGB = 0	0.132
(litterfall, root) | {fundiv, AGB}	root~litterfall+fundiv+AGB+(1|location)	litterfall = 0	0.902
(litterfall, microclim) | {fundiv, AGB, abiotic.PC1, abiotic.PC2, LES, RES}	microclim~litterfall+fundiv+AGB+abiotic.PC1 +abiotic.PC2+LES+RES+(1|location)	litterfall = 0	0.491
(litterfall, herbaceous) | {fundiv, AGB, root}	herbaceous~litterfall+fundiv+AGB+root+(1|location)	litterfall = 0	0.737
(litter.quality, root) | {fundiv, LES, AGB}	root~litter.quality+fundiv+LES+AGB+(1|location)	litter.quality = 0	0.607
(litter.quality, microclim) | {fundiv, LES, RES, abiotic.PC1, abiotic.PC2}	microclim~litter.quality+fundiv+LES+RES +abiotic.PC1 +abiotic.PC2 +(1|location)	litter.quality = 0	0.830
(litter.quality, herbaceous) | {LES, AGB, root}	herbaceous~litter.quality+LES+AGB+root+(1|location)	litter.quality = 0	0.504
(root, microclim) | {fundiv, AGB, LES, RES, abiotic.PC1, abiotic.PC2 }	microclim~root+fundiv+AGB+LES+RES+abiotic.PC1 +abiotic.PC2+(1|location)	root = 0	0.254
(microclim, herbaceous) | {abiotic.PC1, abiotic.PC2, fundiv, LES, RES, AGB, root}	herbaceous~microclim+abiotic.PC1+abiotic.PC2 +fundiv+LES+RES +AGB+root+(1|location)	microclim = 0	0.462

Supplementary Table 3. Results of the best-supported Bayesian multi-level SEM model (*n* = 64 plots; see Methods in the main text) testing the mediation of tree community effects on soil food web multifunctionality. β_{st}, posterior mean of partial regression slope coefficients, standardized by the standard deviation (posterior n = 10,000). CI 95%, 95% posterior credible interval. % of r^2_m, posterior mean proportion of marginal r^2 explained by the predictor variable. r^2_m and r^2_c, posterior means of marginal and conditional r^2, that are the proportion of variance explained by fixed factors only, and by both fixed factors and random factors, respectively. For LES and RES response variables however, r^2_m and r^2_c represent the posterior mean proportion of variance explained by tree species composition only, and both tree species composition and location, respectively. VIF, variance inflation factor. 'Tree species composition' predictor variable was fitted as a random factor. LES, leaf economics spectrum. RES, fine-root economics spectrum. The *p*-values were derived from posterior distributions using a two-tailed test. Significant effects ($p < 0.05$) are reported in bold. †, $p < 0.10$; *, $p < 0.050$; **, $p < 0.010$; ***, $p < 0.001$; ‡, log(x)-transformed; ⁋, log(x+1)-transformed.

Response variable	Predictor variable	β_{st} [CI 95%]	*p*-value	% of r^2_m	r^2_m	r^2_c	VIF
Tree functional diversity	Tree species richness	0.95 [0.87;1.03]	**<0.001*** **		0.90	0.90	
Tree functional composition	Tree species composition				0.79	0.97	
Tree functional composition	Tree species composition				0.22	0.99	
Tree aboveground biomass‡	Tree functional diversity	0.15 [0.00;0.32]	0.062†	7.95	0.21	0.60	1.00
	Tree functional composition (RES)	0.27 [-0.06;0.60]	0.105	26.5			1.01
	Abiotic conditions (PC1)	0.34 [-0.11;0.77]	**0.022***	65.5			1.00
Tree aboveground litterfall‡	Tree functional diversity	0.11 [-0.04;0.27]	0.128	19.4	0.10	0.72	1.07
	Tree aboveground biomass‡	0.27 [-0.04;0.50]	**0.022***	80.6			1.07
Tree leaf litter quality (PC1)	Tree functional composition (LES)	0.55 [0.37;0.72]	**<0.001*** **		0.36	0.60	
Total root biomass‡	Tree functional diversity	-0.21 [-0.42;-0.00]	**0.050***	29.2	0.23	0.32	1.03
	Tree aboveground biomass‡	-0.34 [-0.63;-0.02]	**0.033***	70.8			1.03
Microclimate (PC1)	Tree functional diversity	-0.22 [-0.40;-0.04]	**0.019***	3.6	0.54	0.61	1.02
	Tree functional composition (LES)	0.25 [0.00;0.49]	0.051†	4.7			1.56
	Tree functional composition (RES)	0.60 [0.17;0.97]	**0.011***	23.7			1.74
	Abiotic conditions (PC1)	0.67 [0.23;1.14]	**0.007*** **	28.3			1.27
	Abiotic conditions (PC2)	0.79 [0.31;1.29]	**0.008*** **	39.7			1.16
Understorey plant aboveground biomass (herbaceous)⁋	Tree aboveground biomass‡	-0.44 [-0.70;-0.18]	**<0.001*** **	76.1	0.14	0.62	1.07
	Total root biomass‡	-0.23 [-0.44;-0.02]	**0.029***	24.2			1.07
Soil food web multifunctionality‡	Tree aboveground biomass‡	0.40 [0.15;0.66]	**0.003*** **	19.5	0.50	0.52	1.34
	Tree aboveground litterfall‡	-0.31 [-0.56;-0.06]	**0.019***	12.3			1.12
	Tree leaf litter quality (PC1)	0.44 [0.22;0.66]	**<0.001*** **	22.8			1.04
	Total root biomass‡	0.28 [0.05;0.51]	**0.017***	10.0			1.36
	Understorey plant aboveground biomass (herbaceous)⁋	0.37 [0.14;0.60]	**0.002*** **	16.7			1.21
	Microclimate (PC1)	0.39 [0.16;0.63]	**0.001*** **	18.6			1.13
~~ Tree species richness	~~Tree aboveground litterfall‡	0.08	0.520				
~~Tree functional composition (LES)	~~Tree functional composition (RES)	-0.80	**<0.001*** **				
~~Tree aboveground litterfall‡	~~Tree leaf litter quality (PC1)	-0.35	**0.005*** **				
~~Tree aboveground biomass‡	~~Tree leaf litter quality (PC1)	-0.29	**0.021***				

Supplementary Table 4. **Study site characteristics**. Where applicable, mean ± standard error values are given for each location. Mineral soil data are given for the 0-10 cm depth. ⁋, Ectomycorrhizal tree species; †, Arbuscular mycorrhizal tree species.

	**North Karelia**	**Białowieża**	**Râşca**	**Colline Metallifere**
Country	Finland	Poland	Romania	Italy
Latitude/Longitude (°)	62.9, 29.9	52.8, 23.9	47.3, 26.0	43.2, 11.2
Elevation range (m)	80–200	135–185	600–1,000	260–525
Mean annual air temperature (°C)	4.1 ± 0.1	9.8 ± 0.5	8.2 ± 0.5	12.5 ± 0.8
Mean annual precipitation (mm)	632	597	692	738
Soil type	Podzsol	Cambisol/Luvisol	Eutric Cambisol	Cambisol
Soil texture class	Sandy loam	Sandy loam	Silty clay loam	Silt loam
Sand, silt, clay (%)	48, 47, 5	65, 29, 6	13, 60, 27	17, 65, 18
Soil bulk density (g cm^{-3})	1.03 ± 0.03	1.02 ± 0.02	0.93 ± 0.02	0.88 ± 0.02
Mean annual soil temperature (°C)	5.5 ± 0.2	9.4 ± 0.4	8.2 ± 0.5	12.2 ± 0.5
Mean annual soil moisture (%)	32.8 ± 1.7	24.6 ± 0.8	31.0 ± 2.1	21.3 ± 2.1
Dominant humus type	Mull/Mor	Mull/Moder	Mull	Amphimull/Mull
Forest floor mass (kg m^{-2})	4.27 ± 0.81	2.28 ± 0.41	2.23 ± 0.39	1.81 ± 0.15
Forest floor pH	3.7 ± 0.2	4.8 ± 0.1	4.8 ± 0.1	5.4 ± 0.1
Forest floor C:N	27.5 ± 1.6	27.1 ± 0.9	30.5 ± 0.8	32.4 ± 0.9
Mineral soil C (g kg^{-1})	37.8 ± 3.8	28.4 ± 1.2	49.2 ± 4.0	50.4 ± 3.1
Mineral soil pH	3.9 ± 0.1	3.8 ± 0.1	4.6 ± 0.2	4.6 ± 0.2
Mineral soil C:N	23.8 ± 1.3	16.7 ± 0.5	13.9 ± 0.3	19.9 ± 0.8
Forest type	Boreal	Hemiboreal, nemoral coniferous, mixed broadleaved-coniferous	Mountainous mixed beech	Thermophilous deciduous
Stand age (year)	45 ± 2	101 ± 7	86 ± 5	65 ± 2
Basal area (m^2 ha^{-1})	28.4 ± 2.1	42.4 ± 2.4	51.0 ± 3.5	28.9 ± 0.8
Tree aboveground biomass (kg m^{-2})	14.4 ± 1.0	28.7 ± 1.8	38.0 ± 2.4	21.4 ± 0.9
Leaf area index (m^2 m^{-2})	2.91 ± 0.27	5.10 ± 0.19	5.66 ± 0.18	3.73 ± 0.23
Target tree species	*Betula pendula/pubescens*⁋, *Picea abies*⁋, *Pinus sylvestris*⁋	*Betula pendula*⁋, *Carpinus betulus*⁋, *Picea abies*⁋, *Pinus sylvestris*⁋, *Quercus robur*⁋	*Abies alba*⁋, *Acer pseudoplatanus*†, *Fagus sylvatica*⁋, *Picea abies*⁋	*Castanea sativa, Ostrya carpinifolia, Quercus cerris, Quercus ilex, Quercus petraea*
Number of monospecific/mixed stands	6/3	6/14	8/8	10/9
Soil sampling date (yyyy/mm/dd)	2017/06/12-16	2017/05/05-10	2017/05/22-28	2017/04/10-16

Supplementary Table 5. **List of plant-derived (basal) resources and trophic guilds with trait values used for soil food web reconstruction**. Herbivores, fauna mostly feeding on living plant fine roots; detritivores, fauna mostly feeding on litter; humi-detritivores, fauna feeding on both litter and soil organic matter; microbi-detritivores, fauna feeding on both microbes and litter; bacterivores, fauna mostly feeding on bacteria; fungivores, fauna mostly feeding on fungi; omnivores; fauna feeding on both fauna and other resources; carnivores, fauna mostly feeding on fauna.

			Feeding preferences											Body size		Predator traits			Protection traits				Vertical stratification		
Trophic guild	Trophic group	Abbreviation	LivR	PR	LLit & RLit	WLit	SOM	Ba	FuM	FuS	FuW	FuP	Fauna	Mass mean	Mass SD	Trait	PPMR optimum	PPMR width	Carnivore	Agility	Physical protection	Metabolites	epi	hemi	eu
Living plant fine roots	Living plant fine roots	LivR															1	1	1	1	1	1		1	1
Photosynthates & rhizodeposits	Photosynthates & rhizodeposits	PR															1	1	1	1	1	1		1	1
Dead leaves	Plant litter	LLit															1	1	1	1	1	1	1	1	
Dead roots	Plant litter	RLit															1	1	1	1	1	1		1	1
Dead wood	Plant litter	WLit															1	1	1	1	1	1	1	1	
Soil organic matter	Soil organic matter	SOM															1	1	1	1	1	1			1
Bacteria: Gram-	Bacteria	BaGn		1													1	1	1	1	1	1	1	1	1
Bacteria: Gram+ & Actinomycetes	Bacteria	BaGp			1		1										1	1	1	1	1	1		1	1
Mycorrhizal fungi	Mycorrhizal fungi	FuM		1													1	1	1	1	1	1		1	1
General saprotroph fungi	Decomposer fungi	FuS		1	1		0.5										1	1	1	1	1	1	1	1	1
Wood saprotroph fungi	Decomposer fungi	FuW				1											1	1	1	1	1	1	1	1	1
Plant pathogenic fungi	Plant pathogenic fungi	FuP	1														1	1	1	1	1	1	1	1	1
Nematoda: Plant feeders	Herbivores	NePl	1							0.5		0.5		-6.62	0.5		1	1	1	1	1	1		1	1
Nematoda: Bacterivores	Bacterivores	NeBa						1						-6.14	0.5		1	1	1	1	1	1		1	1
Nematoda: Fungivores	Fungivores	NeFu	0.5						0.5	1				-6.44	0.5		1	1	1	1	1	1		1	1
Nematoda: Omnivores	Omnivores	NeOmn						1					1	-5.05	1		1	1	1	1	1	1		1	1
Nematoda: Predators	Carnivores	NePr						0.5					1	-5.47	1		1	1	0.7	1	1	1		1	1
Acari: Oribatida	Microbi-detritivores	AcOri			1			0.5	0.5	1			0.5	-4.91	0.6		1	1	1	1	0.4	0.7		1	0.5
Acari: Mesostigmata	Carnivores	AcMeso								0.5			1	-4.75	1		1	1	0.7	1	0.7	1		1	0.5
Acari: Prostigmata & Astigmata	Omnivores	AcOther	0.5		0.5			0.5	0.5	1			1	-5.63	0.6		1	1	0.7	1	0.7	1		1	0.5
Collembola: Epedaphic	Fungivores	ColEp			0.5				0.5	1				-3.16	0.5		1	1	1	0.7	1	1	1	0.5	
Collembola: Hemiedaphic	Microbi-detritivores	ColHe			1			0.5	0.5	1			0.5	-4.29	0.6		1	1	1	0.7	1	1		1	0.5

			Feeding preferences											Body size		Predator traits			Protection traits				Vertical stratification		
Trophic guild	Trophic group	Abbreviation	LivR	PR	LLit & RLit	WLit	S	Ba	FuM	FuS	FuW	FuP	Fauna	Mass mean	Mass SD	Trait	PPMR optimum	PPMR width	Carnivore	Agility	Physical protection	Metabolites	epi	hemi	eu
Collembola: Euedaphic	Fungivores	ColEu	0.5		0.5			0.5	0.5	1			0.5	-4.54	0.6		1	1	1	1	1	0.4		1	1
Collembola: Predators	Carnivores	ColPr								0.5			1	-3.74	0.6		1	1	1	1	1	0.4	1	1	
Araneae: Small	Carnivores	ArSm											1	-3.26	0.5	venom and web	0.64	1.44	0.7	1	1	1	0.5	1	
Araneae: Large	Carnivores	ArLa											1	-2.06	1		0.64	1.44	0.7	1	1	1	1	0.5	
Opiliones	Carnivores	Opi											1	-2.31	0.5		1	1	0.7	1	1	1	1		
Diplopoda	Detritivores	Dpod	0.5		1	0.5				0.5				-1.33	0.6		1	1	1	1	0.4	0.7	1	1	0.5
Chilopoda: Geophilomorpha	Carnivores	ChiGe											1	-1.96	0.60	venom	0.8	1.2	0.7	1	1	1		1	1
Chilopoda: Scolopendromorpha	Carnivores	ChiSco											1	-2.23	0.60		0.8	1.2	0.7	1	1	1	0.5	1	0.5
Chilopoda: Lithobiomorpha	Carnivores	ChiLi											1	-2.16	0.60		0.8	1.2	0.7	1	1	1	0.5	1	
Isopoda	Detritivores	Iso	0.5		1	0.5		0.5	0.5	1				-1.70	1		1	1	1	1	0.4	1	1	0.5	
Dermaptera	Omnivores	Der	0.5		1			0.5		0.5			1	-1.42	1		1	1	0.7	1	1	0.4	1	1	
Blattodea	Detritivores	Bla	0.5		1	0.5				0.5			0.5	-1.99	1		1	1	1	1	0.7	0.7	1	0.5	
Formicidae	Omnivores	Ant	1							0.5		0.5	1	-3.30	0.6	pack hunting	0.6	1.6	0.7	1	1	0.7	1	0.5	0.5
Coleoptera: Carabidae	Carnivores	CptCar											1	-1.60	1		1	1	0.7	1	0.4	0.4	1		
Coleoptera: Staphylinidae	Carnivores	CptSta								0.5			1	-2.53	1		1	1	0.7	1	0.4	0.4	1	1	1
Coleoptera: Elateridae (Omnivores)	Omnivores	CptOmn	1		1	1		0.5	0.5	1	0.5	0.5	1	-1.93	1		1	1	1	1	0.4	1		1	1
Coleoptera: Scarabaeidae / Geotrupidae (Herbi-detritivores)	Detritivores	CptHD	1		1	1		0.5	0.5	1	0.5	0.5		-0.51	1		1	1	1	1	0.4	1	1	1	1
Coleoptera: Curculionidae (Root feeders)	Herbivores	CptH	1			0.5				0.5	0.5	0.5		-1.72	1		1	1	1	1	0.4	1		1	1
Lepidoptera	Herbivores	Lepi	1							0.5		0.5		-1.55	1		1	1	1	1	1	1	1	1	
Diptera	Omnivores	Dipt	0.5		1	0.5			0.5	1			1	-1.44	1		1	1	1	1	1	1	1	1	
Lumbricina: Epigeic	Detritivores	EwEp	0.5		1	0.5		0.5		0.5			0.5	-0.78	0.6		4.5	0.75	1	1	1	0.4	0.5	1	
Lumbricina: Anecic	Humi-detritivores	EwAn	0.5		1		1	0.5		0.5			0.5	-0.56	0.7	filtering the environment	4.5	0.75	1	1	1	0.4		1	1
Lumbricina: Endogeic	Humi-detritivores	EwEn	0.5				1	0.5		0.5			0.5	-0.65	0.7		4.5	0.75	1	1	1	0.4			1
Gastropoda: Snails	Detritivores	GaSn	0.5		1	0.5		1	0.5	1			0.5	-1.51	1		1	1	1	1	0.4	0.4	1	1	
Gastropoda: Slugs	Detritivores	GaSl	0.5		1	0.5		1	0.5	1			0.5	-1.01	0.6		1	1	1	1	0.7	0.4	1	1	0.5

Supplementary Table 6. Functional trait values of tree species for each location. LNC, leaf nitrogen content; SLA, specific leaf area; LDMC, leaf dry matter content; RNC, root nitrogen content; SRL, specific root length; RTD, root tissue density; D_m, mean root diameter; RLD, root length density;%Myc, ectomycorrhizal colonisation intensity; ECM, Ectomycorrhizal tree species; AM, Arbuscular mycorrhizal tree species.

Country	Species	Mycorrhizal type	Leaf phenology	LNC (% dry mass)	SLA (mm^2 mg^{-1})	LDMC (mg g^{-1})	RNC (% dry mass)	SRL (m g^{-1})	RTD (g cm^{-3})	D_m (mm)	RLD (cm cm^{-3})	%Myc (number cm^{-1})
Finland	*Betula pendula/pubescens*	ECM	Deciduous	2.37	14.6	385	1.21	42.7	0.333	0.321	0.88	2.3
	Picea abies	ECM	Evergreen	1.10	3.2	431	1.09	16.7	0.362	0.469	1.23	2.3
	Pinus sylvestris	ECM	Evergreen	1.49	3.6	422	1.07	22.8	0.387	0.394	1.16	1.9
Poland	*Betula pendula*	ECM	Deciduous	2.56	14.6	385	1.96	65.4	0.282	0.303	0.97	3.2
	Carpinus betulus	ECM	Deciduous	2.74	11.6	259	1.93	52.6	0.333	0.291	1.08	3.5
	Picea abies	ECM	Evergreen	1.30	3.7	447	1.62	25.7	0.311	0.433	0.39	4.6
	Pinus sylvestris	ECM	Evergreen	2.03	3.6	422	1.81	30.2	0.300	0.409	0.54	3.3
	Quercus robur	ECM	Deciduous	2.50	14.3	277	1.96	50.7	0.297	0.314	0.72	4.0
Romania	*Abies alba*	ECM	Evergreen	1.28	4.6	494	1.36	11.4	0.356	0.583	0.37	2.4
	Acer pseudoplatanus	AM	Deciduous	2.16	12.5	366	1.53	35.2	0.391	0.355	1.41	0.0
	Fagus sylvatica	ECM	Deciduous	2.11	19.3	449	1.46	34.1	0.373	0.324	1.45	3.4
	Picea abies	ECM	Evergreen	1.31	4.2	463	1.29	12.3	0.438	0.542	0.65	2.8
Italy	*Castanea sativa*	ECM	Deciduous	2.62	13.6	265	1.01	27.7	0.454	0.337	0.37	2.9
	Ostrya carpinifolia	ECM	Deciduous	2.32	13.7	242	1.26	40.2	0.407	0.301	0.76	1.8
	Quercus cerris	ECM	Deciduous	2.26	17.9	306	0.92	20.8	0.509	0.354	0.94	1.9
	Quercus ilex	ECM	Evergreen	1.32	6.4	530	0.89	12.8	0.553	0.446	0.35	2.7
	Quercus petraea	ECM	Deciduous	2.19	12.5	281	1.06	22.5	0.475	0.359	2.09	2.2

Supplementary Table 7. Principal component analysis of tree community-weighted mean (CWM) traits. For each tree CWM trait, Pearson's r with the highest absolute value among the two PCA axes is shown in bold.

Tree CWM trait	Correlation with principal components (Pearson's r)		Contribution to principal components (%)	
	PC1 (45.25% of variance)	PC2 (31.48% of variance)	PC1 (45.25% of variance)	PC2 (31.48% of variance)
**Leaf traits**				
Leaf nitrogen content (LNC)	**0.94**	-0.18	21.7	1.1
Specific leaf area (SLA)	**0.63**	-0.59	9.8	12.5
Leaf dry matter content (LDMC)	**-0.78**	0.35	15.0	4.4
**Fine-root traits**				
Root nitrogen content (RNC)	0.53	**0.77**	6.9	20.8
Root tissue density (RTD)	-0.37	**-0.81**	3.5	23.0
Specific root length (SRL)	**0.91**	0.32	20.4	3.7
Root diameter (Dm)	**-0.91**	0.26	20.2	2.3
Root length density (RLD)	0.22	**-0.59**	1.2	12.2
Root ectomycorrhizal colonization intensity (%Myc)	0.24	**0.75**	1.4	20.0

Supplementary Table 8. Initial SEM model, with component linear mixed-effects models in each row. abiotic.PC1, abiotic conditions (PC1); abiotic.PC2, abiotic conditions (PC2), SR, tree species richness; compo, tree species composition; fundiv, tree functional diversity (FDis); composition, tree species composition; LES, tree functional composition corresponding to the leaf economics spectrum (CWM trait PC1); RES, tree functional composition corresponding to the root economics spectrum (CWM trait PC2); AGB, tree aboveground biomass; litterfall, tree aboveground litterfall; litter.quality, tree leaf litter quality (PC1); litter.quality.diversity, functional diversity of tree leaf litter; root, total root biomass;, herbaceous understorey aboveground plant biomass; CN.under, C:N ratio of understorey aboveground plant biomass; SR.under, species richness of understorey aboveground plants; microclim, microclimate (PC1); fertility, soil fertility (PC1); multifun, soil food web multifunctionality.

Model formula
fundiv ~ SR + abiotic.PC1 + abiotic.PC2 + (1|location)
LES ~ abiotic.PC1 + abiotic.PC2 + (1|compo) + (1|location)
RES ~ abiotic.PC1 + abiotic.PC2 + (1|compo) + (1|location)
AGB ~ fundiv + LES + RES + abiotic.PC1 + abiotic.PC2 + (1|location)
litter.quality ~ fundiv + LES + RES + abiotic.PC1 + abiotic.PC2 + (1|location)
litterfall ~ fundiv + LES + RES + AGB + abiotic.PC1 + abiotic.PC2 + (1|location)
root ~ fundiv + LES + RES + AGB + abiotic.PC1 + abiotic.PC2 + (1|location)
litter.quality.diversity ~ fundiv + abiotic.PC1 + abiotic.PC2 + (1|location)
herbaceous.under ~ fundiv + LES + RES + AGB + litter.quality + litterfall + root + abiotic.PC1 + abiotic.PC2 + (1|location)
CN.under ~ fundiv + LES + RES + AGB + litter.quality + litterfall + root + abiotic.PC1 + abiotic.PC2 + (1|location)
SR.under ~ fundiv + LES + RES + AGB + litter.quality + litterfall + root + abiotic.PC1 + abiotic.PC2 + (1|location)
microclim ~ fundiv + LES + RES + AGB + litter.quality + litterfall + root + abiotic.PC1 + abiotic.PC2 + (1|location)
fertility ~ fundiv + LES + RES + AGB + litterfall + litter.quality + root + abiotic.PC1 + abiotic.PC2 + (1|location)
multifun ~ AGB + litterfall + litter.quality (PC1) + litter.quality.diversity + root + herbaceous.under + CN.under + SR.under + microclim + fertility + abiotic.PC1 + abiotic.PC2 + (1|location)

Supplementary Table 9. Nematode families identified, their trophic guild, and their mean individual fresh body mass. The body mass values were retrieved from the Nemaplex database (http://nemaplex.ucdavis.edu).

Nematode family	Trophic guild	Individual fresh body mass (in µg)
Tylenchidae	Herbivore	0.152
Criconematidae	Herbivore	0.638
Dolichodoridae	Herbivore	0.458
Pratylenchidae	Herbivore	2.732
Hoplolaimidae	Herbivore	0.738
Paratylenchidae	Herbivore	0.063
Psilenchidae	Herbivore	0.489
Cephalobidae	Bacterivore	0.435
Plectidae	Bacterivore	0.889
Prismatolaimidae	Bacterivore	0.487
Metateratocephalidae	Bacterivore	0.889
Teratocephalidae	Bacterivore	0.128
Rhabditidae	Bacterivore	5.393
Panagrolaimidae	Bacterivore	4.077
Alaimidae	Bacterivore	0.756
Bunonematidae	Bacterivore	0.122
Monhysteridae	Bacterivore	0.423
Bastianiidae	Bacterivore	0.174
Diplogasteridae	Bacterivore	1.375
Aphelenchoididae	Fungivore	0.205
Diphtherophoridae	Fungivore	0.627
Anguinidae	Fungivore	11.054
Aphelenchidae	Fungivore	0.313
Leptonchidae	Fungivore	0.856
Dorylaimidae	Omnivore	8.834
Tripylidae	Carnivore	2.476
Mononchidae	Carnivore	5.329

Supplementary Table 10. Collembola species identified, their trophic guild and their mean individual fresh and dry body mass. For each species, the mean dry body mass (*M*, in µg) was calculated from the mean body length (*L*, in mm) retrieved from the BETSI database (https://portail.betsi.cnrs.fr/) using the allometric equation: $M = aL^b$, where *a* is the normalisation coefficient and *b* is the exponent. Abdomen length of Symphypleona was used in the original equations and was assumed to be 0.83 of the total body length. Two sets of coefficients coming from two independent studies[22,23] were used for each species (a_1, b_1 and a_2, b_2) and the two estimates of dry body mass were averaged[24]. Fresh body mass was calculated from the resulting average by dividing it by the proportion of the dry weight[23,24].

Family	Species	Trophic guild	Fresh body mass (M, in µg)	Dry body mass (M, in µg)	Fresh body length (in mm)	a_1	b_1	a_2	b_2	Dry weight proportion
Entomobryidae	*Entomobrya corticalis*	Epedaphic	125.64	37.69	1.50	11.75	2.52	14.26	2.71	0.30
Entomobryidae	*Entomobrya nicoleti*	Epedaphic	148.85	44.65	1.60	11.75	2.52	14.26	2.71	0.30
Entomobryidae	*Entomobrya sp.*	Epedaphic	136.94	41.08	1.55	11.75	2.52	14.26	2.71	0.30
Entomobryidae	*Heteromurus nitidus*	Epedaphic	481.19	144.36	2.50	11.75	2.52	14.26	2.71	0.30
Entomobryidae	*Heteromurus sp.*	Epedaphic	481.19	144.36	2.50	11.75	2.52	14.26	2.71	0.30
Entomobryidae	*Lepidocyrtus lignorum*	Epedaphic	202.84	60.85	1.80	11.75	2.52	14.26	2.71	0.30
Entomobryidae	*Lepidocyrtus violaceus*	Epedaphic	136.94	41.08	1.55	11.75	2.52	14.26	2.71	0.30
Entomobryidae	*Orchesella bifasciata*	Epedaphic	267.57	80.27	2.00	11.75	2.52	14.26	2.71	0.30
Entomobryidae	*Orchesella cincta*	Epedaphic	1,166.77	350.03	3.50	11.75	2.52	14.26	2.71	0.30
Entomobryidae	*Orchesella flavescens*	Epedaphic	2,986.80	896.04	5.00	11.75	2.52	14.26	2.71	0.30
Entomobryidae	*Orchesella quinquefasciata*	Epedaphic	2,986.80	896.04	5.00	11.75	2.52	14.26	2.71	0.30
Entomobryidae	*Orchesella sp.*	Epedaphic	1,166.77	350.03	3.50	11.75	2.52	14.26	2.71	0.30
Entomobryidae	*Orchesella villosa*	Epedaphic	3,396.90	1,019.07	5.25	11.75	2.52	14.26	2.71	0.30
Entomobryidae	*Pseudosinella sp.*	Hemiedaphic	43.34	13.00	1.00	11.75	2.52	14.26	2.71	0.30
Isotomidae	*Cryptopygus bipunctatus*	Hemiedaphic	6.13	2.21	0.70	5.62	3.28	8.43	3.22	0.36
Isotomidae	*Desoria grijs*	Hemiedaphic	131.51	47.34	1.80	5.62	3.28	8.43	3.22	0.36
Isotomidae	*Desoria neglecta*	Hemiedaphic	131.51	47.34	1.80	5.62	3.28	8.43	3.22	0.36
Isotomidae	*Desoria paars*	Hemiedaphic	131.51	47.34	1.80	5.62	3.28	8.43	3.22	0.36
Isotomidae	*Desoria sp.*	Hemiedaphic	131.51	47.34	1.80	5.62	3.28	8.43	3.22	0.36
Isotomidae	*Folsomia quadrioculata*	Hemiedaphic	58.17	20.94	1.40	5.62	3.28	8.43	3.22	0.36
Isotomidae	*Folsomia sp.*	Hemiedaphic	58.17	20.94	1.40	5.62	3.28	8.43	3.22	0.36
Isotomidae	*Folsomides angularis*	Hemiedaphic	11.39	4.10	0.88	6.46	2.99	5.62	2.80	0.36
Isotomidae	*Folsomides parvulus*	Hemiedaphic	12.36	4.45	0.90	6.46	2.99	5.62	2.80	0.36
Isotomidae	*Isotoma anglicana*	Epedaphic	1,757.29	632.63	4.00	5.62	3.28	8.43	3.22	0.36
Isotomidae	*Isotoma caerulea*	Epedaphic	1,757.29	632.63	4.00	5.62	3.28	8.43	3.22	0.36
Isotomidae	*Isotoma sp.*	Epedaphic	1,757.29	632.63	4.00	5.62	3.28	8.43	3.22	0.36
Isotomidae	*Isotomiella minor*	Euedaphic	26.59	9.57	1.10	5.62	3.28	8.43	3.22	0.36
Isotomidae	*Isotomurus italicus*	Epedaphic	382.05	137.54	2.50	5.62	3.28	8.43	3.22	0.36
Isotomidae	*Isotomurus sp.*	Epedaphic	382.05	137.54	2.50	5.62	3.28	8.43	3.22	0.36
Isotomidae	*Parisotoma notabilis*	Hemiedaphic	19.51	7.03	1.00	5.62	3.28	8.43	3.22	0.36
Isotomidae	*Proisotoma sp.*	Epedaphic	22.86	8.23	1.05	5.62	3.28	8.43	3.22	0.36
Isotomidae	*Tetracanthella sp.*	Hemiedaphic	35.27	12.70	1.20	5.62	3.28	8.43	3.22	0.36
Isotomidae	*Vertagopus sp.*	Epedaphic	120.02	43.21	1.75	5.62	3.28	8.43	3.22	0.36
Onychiuridae	*Onychiuridae sp.*	Euedaphic	96.71	29.01	1.90	4.27	2.75	5.60	2.77	0.30

Family	Species	Trophic guild	Fresh body mass (M, in µg)	Dry body mass (M, in µg)	Fresh body length (in mm)	a1	b1	a2	b2	Dry weight proportion
Onychiuridae	*Onychiurus sp.*	Euedaphic	96.71	29.01	1.90	4.27	2.75	5.60	2.77	0.30
Onychiuridae	*Protaphorura sp.*	Epedaphic	111.43	33.43	2.00	4.27	2.75	5.60	2.77	0.30
Tomoceridae	*Pogonognathellus flavescens*	Epedaphic	6,163.09	1,540.77	6.00	9.20	2.74	14.26	2.71	0.25
Tomoceridae	*Pogonognathellus longicornis*	Epedaphic	3,751.59	937.90	5.00	9.20	2.74	14.26	2.71	0.25
Tomoceridae	*Tomocerina minuta*	Hemiedaphic	331.15	82.79	2.05	9.20	2.74	14.26	2.71	0.25
Tomoceridae	*Tomocerus vulgaris*	Epedaphic	2,043.47	510.87	4.00	9.20	2.74	14.26	2.71	0.25
Neelidae	*Megalothorax minimus*	Euedaphic	19.23	5.77	0.40	39.99	2.11	39.99	2.11	0.30
Hypogastruridae	*Ceratophysella denticulata*	Hemiedaphic	120.41	36.12	1.80	9.77	2.55	5.60	2.77	0.30
Hypogastruridae	*Ceratophysella sp.*	Hemiedaphic	120.41	36.12	1.80	9.77	2.55	5.60	2.77	0.30
Hypogastruridae	*Choreutinula inermis*	Hemiedaphic	25.62	7.69	1.00	9.77	2.55	5.60	2.77	0.30
Hypogastruridae	*Choreutinula sp.*	Hemiedaphic	25.62	7.69	1.00	9.77	2.55	5.60	2.77	0.30
Hypogastruridae	*Schoetella sp.*	Hemiedaphic	25.62	7.69	1.00	9.77	2.55	5.60	2.77	0.30
Hypogastruridae	*Schoetella ununguiculata*	Hemiedaphic	25.62	7.69	1.00	9.77	2.55	5.60	2.77	0.30
Hypogastruridae	*Willemia denisi*	Euedaphic	11.00	3.30	0.73	9.77	2.55	5.60	2.77	0.30
Hypogastruridae	*Willemia sp.*	Euedaphic	11.00	3.30	0.73	9.77	2.55	5.60	2.77	0.30
Isotomidae	*Anurophorus laricis*	Hemiedaphic	62.10	18.63	1.40	9.77	2.55	5.60	2.77	0.30
Isotomidae	*Anurophorus septentrionalis*	Hemiedaphic	32.92	9.87	1.10	9.77	2.55	5.60	2.77	0.30
Neanuridae	*Anurida grey*	Predators	25.62	7.69	1.00	9.77	2.55	5.60	2.77	0.30
Neanuridae	*Anurida sp.*	Predators	25.62	7.69	1.00	9.77	2.55	5.60	2.77	0.30
Neanuridae	*Friesea mirabilis*	Predators	68.11	20.43	1.45	9.77	2.55	5.60	2.77	0.30
Neanuridae	*Friesea subterranea*	Predators	12.03	3.61	0.75	9.77	2.55	5.60	2.77	0.30
Neanuridae	*Frisea sp.*	Predators	16.71	5.01	0.85	9.77	2.55	5.60	2.77	0.30
Neanuridae	*Lathriopyga longiseta*	Predators	286.46	85.94	2.50	9.77	2.55	5.60	2.77	0.30
Neanuridae	*Micranurida forslundi*	Predators	19.42	5.83	0.90	9.77	2.55	5.60	2.77	0.30
Neanuridae	*Micranurida granulata*	Predators	4.15	1.24	0.50	9.77	2.55	5.60	2.77	0.30
Neanuridae	*Micranurida pygmaea*	Predators	4.15	1.24	0.50	9.77	2.55	5.60	2.77	0.30
Neanuridae	*Micranurida sp.*	Predators	4.15	1.24	0.50	9.77	2.55	5.60	2.77	0.30
Neanuridae	*Monobella sp.*	Predators	74.47	22.34	1.50	9.77	2.55	5.60	2.77	0.30
Neanuridae	*Neanura alba*	Predators	120.41	36.12	1.80	9.77	2.55	5.60	2.77	0.30
Neanuridae	*Neanura muscorum*	Predators	696.89	209.07	3.50	9.77	2.55	5.60	2.77	0.30
Neanuridae	*Pseudachorutes dubius*	Predators	463.67	139.10	3.00	9.77	2.55	5.60	2.77	0.30
Odontellidae	*Odontella sp.*	Hemiedaphic	25.62	7.69	1.00	9.77	2.55	5.60	2.77	0.30
Odontellidae	*Superodontella lamellifera*	Hemiedaphic	68.11	20.43	1.45	9.77	2.55	5.60	2.77	0.30
Onychiuridae	*Kalaphorura burmeisteri*	Euedaphic	14.25	4.27	0.80	9.77	2.55	5.60	2.77	0.30
Onychiuridae	*Micraphorura absoloni*	Euedaphic	14.25	4.27	0.80	9.77	2.55	5.60	2.77	0.30
Onychiuridae	*Micraphorura sp.*	Euedaphic	14.25	4.27	0.80	9.77	2.55	5.60	2.77	0.30
Tullbergiidae	*Tullbergiidae*	Euedaphic	6.69	2.01	0.60	9.77	2.55	5.60	2.77	0.30
Arrhopalitidae	*Arrhopalites principalis*	Hemiedaphic	277.32	58.24	1.00	190.55	3.63	39.63	3.80	0.21
Arrhopalitidae	*Arrhopalites sp.*	Hemiedaphic	548.03	115.09	1.00	190.55	3.63	39.63	3.80	0.21

Family	Species	Trophic guild	Fresh body mass (M, in µg)	Dry body mass (M, in µg)	Fresh body length (in mm)	a1	b1	a2	b2	Dry weight proportion
Bourletiellidae	*Deuterosminthurus bicinctus*	Epedaphic	242.40	50.90	0.80	190.55	3.63	39.63	3.80	0.21
Dicyrtomidae	*Dicyrtoma fusca*	Epedaphic	6,915.72	1,452.30	2.00	190.55	3.63	39.63	3.80	0.21
Dicyrtomidae	*Dicyrtomidae sp.*	Epedaphic	6,915.72	1,452.30	2.00	190.55	3.63	39.63	3.80	0.21
Dicyrtomidae	*Dicyrtomina ornata*	Epedaphic	15,648.81	3,286.25	2.50	190.55	3.63	39.63	3.80	0.21
Dicyrtomidae	*Dicyrtomina sp.*	Epedaphic	15,648.81	3,286.25	2.50	190.55	3.63	39.63	3.80	0.21
Katiannidae	*Sminthurinus aureus*	Epedaphic	372.84	78.30	0.90	190.55	3.63	39.63	3.80	0.21
Katiannidae	*Sminthurinus sp.*	Epedaphic	271.26	56.96	0.83	190.55	3.63	39.63	3.80	0.21
Katiannidae	*Sminturinus bimaculatus*	Epedaphic	148.79	31.25	0.70	190.55	3.63	39.63	3.80	0.21
Katiannidae	*Sminturinus elegans*	Epedaphic	148.79	31.25	0.70	190.55	3.63	39.63	3.80	0.21
Katiannidae	*Sminturinus signatus*	Epedaphic	271.26	56.96	0.83	190.55	3.63	39.63	3.80	0.21
Sminthuridae	*Allacma fusca*	Epedaphic	56,491.17	11,863.14	3.55	190.55	3.63	39.63	3.80	0.21
Sminthuridae	*Caprainea marginata*	Epedaphic	1875.71	393.90	1.40	190.55	3.63	39.63	3.80	0.21
Sminthuridae	*Caprainea sp.*	Epedaphic	1875.71	393.90	1.40	190.55	3.63	39.63	3.80	0.21
Sminthuridae	*Lipothrix lubbocki*	Epedaphic	6915.72	1452.30	2.00	190.55	3.63	39.63	3.80	0.21
Sminthuridae	*Lipothrix sp.*	Epedaphic	6915.72	1452.30	2.00	190.55	3.63	39.63	3.80	0.21
Sminthuridae	*Spatulosminthurus flaviceps*	Epedaphic	3,056.95	641.96	1.60	190.55	3.63	39.63	3.80	0.21
Sminthurididae	*Sminthuridae sp.*	Epedaphic	548.03	115.09	1.00	190.55	3.63	39.63	3.80	0.21
Sminthurididae	*Sminthurides sp.*	Epedaphic	43.51	9.14	0.50	190.55	3.63	39.63	3.80	0.21
Sminthurididae	*Sphaeridia pumilis*	Epedaphic	19.26	4.04	0.40	190.55	3.63	39.63	3.80	0.21
Sminthurididae	*Sphaeridia sp.*	Epedaphic	19.26	4.04	0.40	190.55	3.63	39.63	3.80	0.21
Symphypleona	Symphypleona	Epedaphic	1,703.59	357.75	1.36	190.55	3.63	39.63	3.80	0.21

Supplementary Table 11. Earthworm species identified, and their ecological groups. Earthworm species were classified based on the DriloBASE database (http://taxo.drilobase.org).

Species	Ecological group
Allolobophora carpathica	Endogeic
Allolobophora sturanyi dacidoides	Endogeic
Aporrectodea caliginosa	Endogeic
Aporrectodea rosea	Endogeic
Dendrobaena attemsi	Epigeic
Dendrobaena octaedra	Epigeic
Dendrobaena platyura	Anecic
Dendrobaena sp.	Epigeic
Lumbricus rubellus	Epigeic
Lumbricus sp.	Epigeic
Lumbricus terrestris	Anecic
Octodrillus complatus	Endogeic
Octolasium lacteum	Endogeic

Supplementary Table 12. Regression parameters used to calculate individual metabolic rates for faunal consumers. This was based on this equation: $\ln I = \ln i_0 + a \ln M - E(1/kT)$, where I is the metabolic rate, i_0 is a normalisation factor, a is the allometric exponent, E is the activation energy, k is the Boltzmann constant, and T is the temperature in Kelvin. The taxa-specific parameters were used if available, and general parameters were used otherwise. These parameter values are from Ehnes *et al.* (2011)[136] and unpublished data (Roswitha Ehnes).

Regression model	Phylogenetic contrast	Taxa	ln i_0	a	E
Linear	General	Nematoda, Gasteropoda	23.055	0.695	0.686
Phylogenetic	Arachnida	Araneae, Opiliones	24.581	0.565	0.709
Phylogenetic	Chilopoda	Chilopoda	28.253	0.558	0.803
Phylogenetic	Clitellata	Clitellata	12.442	0.801	0.443
Phylogenetic	Coleoptera	Coleoptera	21.418	0.738	0.639
Phylogenetic	Insecta	Blattodea, Collembola, Dermaptera, Diptera, Formicidae, Lepidoptera	21.972	0.759	0.657
Phylogenetic	Isopoda	Isopoda	23.169	0.554	0.687
Phylogenetic	Mesostigmata	Mesostigmata	9.674	0.690	0.379
Phylogenetic	Oribatida	Oribatida	22.023	0.679	0.706
Phylogenetic	Progoneata	Diplopoda	22.347	0.571	0.670
Phylogenetic	Prostigmata	Astigmata, Prostigmata	10.281	0.660	0.413

Supplementary Table 13. Diet-specific assimilation efficiency values averaged across all plots for faunal consumers. These values were calculated from Jochum *et al.* (2017)[36] based on food N content, and corrected for temperature based on Lang *et al.* (2017)[37]. The growing season soil temperature averaged across all plots was used for temperature correction. Note that the assimilation efficiency of living plant fine roots by herbivores is much lower than the assimilation efficiency of plant leaves (0.545) based on Lang *et al.* (2017)[37] but this is consistent with the assimilation efficiency of plant roots (~0.180) based on Gan & Wickings (2020)[137].

Food	Food N content	Assimilation efficiency uncorrected for temperature	Assimilation efficiency corrected for temperature
Living plant fine roots	0.014	0.191	0.168
Dead leaves	0.011	0.170	0.148
Dead roots	0.014	0.191	0.168
Dead wood	0.003	0.124	0.102
Soil organic matter	0.002	0.121	0.099
Bacteria: Gram-	0.077	0.820	0.803
Bacteria: Gram+	0.077	0.820	0.803
Mycorrhizal fungi	0.034	0.379	0.363
General saprotroph fungi	0.040	0.447	0.430
Wood saprotroph fungi	0.034	0.379	0.363
Plant pathogenic fungi	0.040	0.447	0.430
Nematoda: Plant feeders	0.053	0.599	0.582
Nematoda: Bacterivores	0.053	0.599	0.582
Nematoda: Fungivores	0.053	0.599	0.582
Nematoda: Omnivores	0.053	0.599	0.582
Nematoda: Predators	0.053	0.599	0.582
Oribatida	0.089	0.890	0.874
Mesostigmata	0.105	0.945	0.929
Prostigmata & Astigmata	0.101	0.935	0.918
Collembola: Epedaphic	0.109	0.954	0.938
Collembola: Hemiedaphic	0.109	0.954	0.938
Collembola: Euedaphic	0.109	0.954	0.938
Collembola: Predators	0.109	0.954	0.938
Araneae: Small	0.111	0.958	0.941
Araneae: Large	0.111	0.958	0.941
Opiliones	0.120	0.972	0.956
Diplopoda	0.054	0.610	0.593
Chilopoda: Geophilomorpha	0.117	0.968	0.951
Chilopoda: Scolopendromorpha	0.117	0.968	0.951
Chilopoda: Lithobiomorpha	0.117	0.968	0.951
Isopoda	0.066	0.733	0.717
Dermaptera	0.092	0.903	0.887
Blattodea	0.111	0.958	0.941
Formicidae	0.113	0.962	0.945
Coleoptera: Carabidae	0.100	0.932	0.915
Coleoptera: Staphylinidae	0.100	0.932	0.915
Coleoptera: Elateridae	0.090	0.895	0.878
Coleoptera: Scarabaeidae/Geotrupidae	0.090	0.895	0.878
Coleoptera: Curculionidae	0.090	0.895	0.878
Lepidoptera	0.082	0.854	0.837
Diptera	0.096	0.919	0.902
Lumbricina: Epigeic	0.122	0.975	0.958
Lumbricina: Anecic	0.122	0.975	0.958
Lumbricina: Endogeic	0.122	0.975	0.958
Gastropoda: Snails	0.042	0.470	0.454
Gastropoda: Slugs	0.094	0.911	0.894

Supplementary Table 14. Regression parameters used to calculate the temperature correction for assimilation efficiency. $\acute{\varepsilon}_0$ is the normalization constant of the assimilation efficiency, E_ε is the activation energy for assimilation efficiency (eV), and ε_0 is the assimilation efficiency at 20°C. The parameter values are from Lang *et al.* (2017)[37].

Consumer type	Consumed food type	$\acute{\varepsilon}_0$	E_ε	ε_0
Herbivores	Living plant fine roots	-1.670		0.545
Detritivores	Plant litter and soil organic matter	0.179	0.164	0.158
Carnivores	Fauna and microbes	2.266		0.906

Supplementary Table 15. Multicollinearity checking. Variance inflation factor (VIF) values are provided for each fixed factor of both the taxonomic and functional models (equations 1 and 2 in the main text) predicting soil food web multifunctionality.

Model type	Fixed factor	VIF
Taxonomic	Tree species richness	1.01
	Abiotic conditions (PC1)	1.10
	Abiotic conditions (PC2)	1.11
Functional	Tree functional diversity	1.01
	Tree functional composition (LES)	2.26
	Tree functional composition (RES)	2.29
	Abiotic conditions (PC1)	1.16
	Abiotic conditions (PC2)	1.13

Supplementary Table 16. Results of Bayesian multi-level models (equations 2 and 3; *n* = 64 plots; see Methods in the main text) testing tree community effects on soil food web multifunctionality using both the 'average' and 'threshold' approaches. Both the leaf and fine-root economics spectra variables representing tree functional composition ranged from slow/conservative to fast/acquisitive economic strategies (two first PCA axes of CWM tree traits; Extended Data Fig. 4a). % var, posterior mean proportion of summed variance components explained. β_{st}, posterior mean of slope regression coefficients standardised by standard deviation. CI 95%, 95% posterior credible intervals. BF, Bayes factor calculated as the ratio of marginal likelihoods of two models: an alternative model (m_1) including the random factor tested over a null model (m_0) not including the random factor. Values of BF > 1 and > 10 respectively indicates moderate and strong support in favour of the random factor effect. Significant effects ($p < 0.05$ or BF > 1) are reported in bold. The *p*-values were derived from posterior distributions using a two-tailed test. †, $p < 0.10$; *, $p < 0.050$; **, $p < 0.010$; ***, $p < 0.001$.

	Taxonomic approach for tree community effects						Functional approach for tree community effects								
	Three species mixing			Species composition		Model r^2	Functional diversity			Functional composition: Leaf economics spectrum			Functional composition: Fine-root economics spectrum		Model r^2
	% var	β_{st} [CI 95%]	*p*-value	% var	BF		% var	β_{st} [CI 95%]	*p*-value	% var	β_{st} [CI 95%]	*p*-value	β_{st} [CI 95%]	*p*-value	
Average	4.43	-0.19 [-0.46;0.10]	0.192	22.26	**4.24**	0.47	1.57	-0.13 [-0.35;0.10]	0.254	36.67	0.49 [0.20;0.80]	**0.002****	0.68 [0.20;1.17]	**0.009****	0.38
10% threshold	4.97	-0.20 [-0.47;0.07]	0.148	10.48	0.64	0.32	3.88	-0.20 [-0.44;0.03]	0.087†	15.84	0.31 [0.00;0.64]	0.053†	0.26 [-0.27;0.79]	0.306	0.30
20% threshold	4.81	-0.19 [-0.50;0.11]	0.211	29.23	**8.64**	0.47	1.81	-0.13 [-0.37;0.11]	0.281	27.57	0.35 [0.02;0.72]	**0.036***	0.62 [0.10;1.25]	**0.022***	0.28
30% threshold	1.87	-0.07 [-0.34;0.19]	0.558	9.27	0.71	0.39	0.66	-0.00 [-0.22;0.22]	0.989	37.30	0.47 [0.15;0.75]	**0.008****	0.72 [0.21;1.21]	**0.008****	0.41
40% threshold	4.10	-0.18 [-0.45;0.08]	0.169	6.62	0.48	0.29	1.93	-0.14 [-0.37;0.09]	0.231	29.75	0.45 [0.11;0.76]	**0.010***	0.54 [0.00;1.03]	0.050†	0.33
50% threshold	8.59	-0.29 [-0.56;-0.02]	**0.037***	10.72	0.70	0.32	4.78	-0.26 [-0.50;-0.03]	**0.025***	28.98	0.48 [0.18;0.80]	**0.003****	0.52 [0.01;1.01]	**0.044***	0.33
60% threshold	2.08	-0.07 [-0.35;0.22]	0.647	9.00	0.48	0.18	1.65	-0.08 [-0.34;0.18]	0.527	14.16	0.21 [-0.13;0.58]	0.220	0.32 [-0.24;0.90]	0.243	0.15
70% threshold	1.70	0.02 [-0.26;0.29]	0.906	9.22	0.56	0.24	1.37	0.05 [-0.21;0.30]	0.709	10.38	0.17 [-0.18;0.50]	0.310	0.16 [-0.39;0.68]	0.512	0.20
80% threshold	2.60	0.10 [-0.19;0.38]	0.505	12.88	0.69	0.27	1.46	0.06 [-0.20;0.31]	0.650	16.67	0.27 [-0.10;0.60]	0.139	0.28 [-0.33;0.82]	0.313	0.22
90% threshold	3.68	0.14 [-0.15;0.43]	0.317	20.8	**1.44**	0.39	1.65	0.08 [-0.16;0.32]	0.497	22.10	0.34 [0.02;0.66]	**0.040***	0.35 [-0.17;0.84]	0.166	0.28

Supplementary Table 17. Results of Bayesian multi-level random slope models (following equations 2 and 3; *n* = 64 plots; see Methods in the main text) for soil food web functioning. Variables from the leaf and fine-root economics spectra (LES and RES) representing tree functional composition ranged from slow/conservative to fast/acquisitive attributes (Extended Data Fig. 4a). % var, posterior mean of proportion explained of summed variance components. β_{st}, posterior mean of slope regression coefficients standardised by standard deviation. CI 95%, 95% posterior credible intervals. BF, Bayes factor (see Methods). Significant effects ($p < 0.05$ or BF > 1) are reported in bold. †, $p < 0.10$; *, $p < 0.050$; **, $p < 0.010$; ***, $p < 0.001$. SOM, soil organic matter. Δ_i, difference in LOO information criterion values between the random-slope model and the random-intercept model. Negative Δ_i indicate better goodness-of-fit for the random-slope model than the random-intercept model. When $|\Delta_i| < 6$, difference in goodness-of-fit is considered small, *i.e.* both models have comparable support in the data[138].

	Taxonomic approach for tree community effects						Functional approach for tree community effects								
	Three species mixing			Species composition		Δ_i	Functional diversity			Functional composition					Δ_i
										Leaf economics spectrum			Fine-root economics spectrum		
	% var	β_{st} [CI 95%]	*p*-value	% var	BF		% var	β_{st} [CI 95%]	*p*-value	% var	β_{st} [CI 95%]	*p*-value	β_{st} [CI 95%]	*p*-value	
Carnivory	3.75	0.00 [-0.38;0.40]	0.997	7.19	0.601	-1.70	1.20	0.03 [-0.21;0.28]	0.837	32.15	0.35 [0.00;0.71]	**0.049***	0.48 [-0.01;0.96]	0.054†	+1.09
Microbivory	3.94	-0.04 [-0.43;0.34]	0.781	13.62	0.87	+3.19	0.68	-0.03 [-0.25;0.19]	0.775	37.54	0.32 [-0.02;0.61]	0.061†	0.78 [0.26;1.20]	**0.014***	+1.11
Fungivory	3.34	-0.01 [-0.38;0.35]	0.942	8.46	0.52	+2.81	0.63	0.01 [-0.23;0.25]	0.916	26.52	0.27 [-0.12;0.60]	0.133	0.73 [0.19;1.19]	**0.013***	+1.82
Bacterivory	3.38	-0.10 [-0.41;0.22]	0.478	4.50	0.62	-1.28	1.92	-0.11 [-0.26;0.06]	0.194	24.01	0.20 [-0.12;0.51]	0.164	0.40 [-0.12;0.78]	0.066†	+0.67
Detritivory	5.25	-0.14 [-0.50;0.24]	0.422	8.28	0.46	-1.35	2.76	-0.15 [-0.42;0.14]	0.277	16.24	0.20 [-0.21;0.59]	0.263	0.33 [-0.27;0.91]	0.214	+0.59
Soil engineering	6.06	-0.18 [-0.51;0.13]	0.223	5.95	0.43	0.07	2.61	-0.16 [-0.44;0.11]	0.220	14.62	0.34 [-0.19;0.86]	0.161	0.07 [-0.64;0.74]	0.796	-3.74
SOM decomposition	9.50	-0.25 [-0.58;0.09]	0.130	24.24	**3.52**	-0.89	4.75	-0.22 [-0.50;-0.08]	0.121	9.94	0.19 [-0.28;0.66]	0.343	0.02 [-0.61;0.69]	0.947	+0.33
Litter engineering	4.94	0.11 [-0.28;0.49]	0.543	13.70	**1.06**	+1.95	2.57	0.15 [-0.12;0.41]	0.263	21.15	0.29 [-0.22;0.70]	0.176	0.36 [-0.32;0.89]	0.238	+2.27
Litter decomposition	5.72	-0.05 [-0.52;0.41]	0.819	3.54	0.39	-4.85	1.53	-0.06 [-0.37;0.23]	0.667	21.28	0.14 [-0.33;0.57]	0.452	0.48 [-0.12;1.08]	0.102	+0.49
Herbivory	6.77	-0.15 [-0.56;0.28]	0.427	11.31	0.94	+2.69	1.54	-0.12 [-0.41;0.21]	0.389	18.07	0.43 [-0.12;0.95]	0.105	0.38 [-0.25;0.98]	0.203	-4.02
Rhizophagy	5.27	-0.13 [-0.51;0.24]	0.422	8.28	0.46	+5.02	1.58	-0.15 [-0.43;0.15]	0.266	21.49	0.25 [-0.37;0.86]	0.354	0.66 [-0.03;1.47]	0.066†	-3.27
Root pathogenicity	3.32	-0.02 [-0.39;0.34]	0.880	26.71	**4.98**	+0.61	1.04	0.03 [-0.24;0.33]	0.827	19.31	0.45 [0.05;0.93]	0.067†	0.20 [-0.55;0.87]	0.457	-0.97
Plant C allocation to soil by roots	4.32	-0.08 [-0.43;0.31]	0.601	8.95	0.75	+0.27	1.65	-0.08 [-0.37;0.23]	0.549	10.83	0.07 [-0.42;0.61]	0.799	0.16 [-0.59;0.98]	0.707	+3.89
Soil food web multifunctionality	8.27	-0.16 [-0.62;0.32]	0.435	20.51	**2.07**	-0.21	1.67	-0.12 [-0.39;0.17]	0.368	31.97	0.47 [0.03;0.88]	**0.037***	0.60 [0.01;1.13]	**0.046***	-0.80

Supplementary Table 18. Results of Bayesian multi-level models testing for omitted variable bias from unmeasured location-level confounders in the analyses of tree community effects on soil food web multifunctionality following the functional approach (*n* = 64 plots). β_{st}, posterior mean of slope regression coefficients standardised by standard deviation. CI 95%, 95% posterior credible intervals. The *p*-values were derived from posterior distributions using a two-tailed test.

	**Original linear mixed-effects model (equation 2 in the main text)**		**Correlated random effects design model (Mundlak approach)**			
	Original causal variable (x_{ij})		Original causal variable (x_{ij})		Location-mean causal variable ($\bar{x}_i$, contextual effect)	
	β_{st} [CI 95%]	*p*-value	β_{st} [CI 95%]	*p*-value	β_{st} [CI 95%]	*p*-value
Functional diversity	-0.13 [-0.35;0.10]	0.254	-0.13 [-0.36;0.10]	0.276	-0.05 [-1.01;0.91]	0.874
Functional composition (leaf economics spectrum)	0.49 [0.20;0.80]	**0.002****	0.54 [0.16;0.92]	**0.008****	-0.26 [-1.57;0.80]	0.630
Functional composition (fine-root economics spectrum)	0.68 [0.20;1.17]	**0.009****	0.67 [-0.01;1.34]	0.054†	0.06 [-1.19;1.40]	0.937

Supplementary Table 19. Adjacency matrix of feeding preferences averaged across all plots. The values indicate the trophic interaction strengths [0-1] between a consumer and its resources. The sum of all resource values of a given consumer is always equal to one. Consumers and food resources are respectively in columns and rows. See Supplementary Table 5 for abbreviation meanings.

	BaGn	BaGp	FuM	FuS	FuW	FuP	NePl	NeBa	NeFu	NeOmn	NePr	AcOri	AcMeso	AcOther	ColEp	ColHe	ColEu	ColPr	ArSm	ArLa	Opi	Dpod	ChiGe	ChiSco	ChiLi	Iso	Der	Bla	Ant	CptCar	CptSta	CptOmn	CptHD	CptH	Lepi	Dipt	EwEp	EwAn	EwEn	GaSn	GaSl
LivRoot						1.00	0.71		0.14					0.02			0.05					0.02				0.01	0.01	0.01	0.36			0.07	0.08	0.65	0.71		0.01				
PR	1.00		1.00	0.04																																					
LeafLit		0.30		0.63								0.48		0.10	0.24	0.48	0.18					0.86				0.58	0.38	0.83				0.23	0.55			0.32	0.70	0.13		0.39	0.38
RootLit		0.02		0.04								0.05		0.01	0.01	0.05	0.02					0.04				0.01	0.01	0.02				0.03	0.03			0.01	0.03	0.02	0.01	0.01	0.02
WoodLit					1.00																	0.01						0.01				0.01	0.02	0.02			0.01				
SOM		0.69		0.29																																		0.65	0.72		
BaGn								0.25		0.13	0.07						0.01									0.01	0.01										0.01	0.01	0.02	0.03	0.03
BaGp								0.75		0.37	0.21	0.01		0.01		0.01	0.02									0.02	0.02						0.01				0.04	0.03	0.05	0.09	0.09
FuM									0.46			0.22		0.18	0.22	0.22	0.35									0.11						0.09	0.14			0.06				0.16	0.18
FuS							0.27		0.39			0.18	0.17	0.15	0.53	0.18	0.29	0.17				0.08				0.27	0.07	0.07	0.14		0.17	0.07	0.18	0.31	0.27	0.11	0.13	0.10	0.14	0.27	0.26
FuW																																		0.01							
FuP							0.01																						0.01					0.01	0.01						
NePl										0.07	0.14	0.01	0.08	0.15			0.01	0.01																			0.03	0.02	0.03		
NeBa										0.13	0.20	0.02	0.16	0.16		0.01	0.02	0.03	0.01										0.01		0.01							0.02	0.02		
NeFu										0.01	0.02		0.01	0.02																											
NeOmn										0.14	0.18	0.01	0.22	0.10		0.02	0.03	0.15	0.08	0.01			0.03	0.03	0.02				0.03		0.05	0.02					0.02	0.01	0.02		
NePr										0.01	0.01		0.02	0.01				0.01																							
AcOri										0.04	0.04		0.09	0.02		0.01	0.01	0.09	0.04				0.01	0.01	0.01				0.02		0.01										
AcMeso										0.03	0.04		0.06	0.02			0.01	0.05	0.04	0.01			0.01	0.01	0.01				0.01		0.01	0.01									
AcOther											0.01		0.01	0.01																											
ColEp										0.01	0.01		0.02					0.16	0.31	0.19	0.58		0.08	0.21	0.23		0.05	0.02	0.16	0.20	0.18	0.04				0.05					
ColHe										0.04	0.05		0.11	0.01		0.01	0.01	0.18	0.14	0.02			0.03	0.04	0.03				0.05		0.05	0.02									
ColEu										0.01	0.01		0.01					0.02	0.01																						
ColPr																		0.01	0.02		0.01			0.01	0.01				0.01		0.01										
ArSm																			0.01																						
ArLa																		0.01	0.02	0.03	0.03		0.01	0.02	0.03		0.01		0.01	0.04	0.02	0.01				0.01					
Opi																					0.01									0.01											
Dpod																			0.01	0.04	0.02		0.04	0.04	0.03		0.03		0.01	0.05	0.02	0.02				0.03					
ChiGe																			0.01	0.01			0.06	0.03	0.03		0.01		0.01		0.02	0.02				0.01					
ChiSco																																									
ChiLi																			0.02	0.02	0.02		0.03	0.04	0.04		0.01		0.01	0.02	0.02	0.01				0.01					
Iso																		0.01	0.01	0.03	0.02		0.01	0.02	0.02		0.01		0.01	0.03	0.01					0.01					
Der																			0.01	0.01	0.01		0.01	0.01	0.01		0.01			0.01	0.01					0.01					
Bla																																									
Ant																			0.01		0.01																				
CptCar																				0.01	0.01				0.01					0.02											
CptSta																																									
CptOmn																		0.01	0.01	0.01			0.04	0.03	0.02		0.01		0.01		0.02	0.02				0.01					
CptHD													0.01					0.01	0.05	0.17	0.06		0.17	0.12	0.11		0.09	0.01	0.04	0.17	0.11	0.09				0.09				0.01	0.01
CptH																			0.01	0.01			0.03	0.02	0.01		0.01				0.01	0.01				0.01					
Lepi																		0.01	0.01	0.02	0.02		0.02	0.02	0.02		0.01		0.01	0.03	0.01	0.01				0.01					
Dipt													0.01					0.03	0.08	0.15	0.11		0.10	0.12	0.14		0.09	0.01	0.04	0.18	0.08	0.05				0.09				0.01	0.01
EwEp																			0.02	0.12	0.02		0.08	0.08	0.10		0.08	0.01	0.01	0.10	0.04	0.04				0.08				0.01	0.01
EwAn																				0.01			0.03	0.01	0.01		0.01				0.01	0.02				0.01					
EwEn																							0.15	0.05					0.01		0.05	0.08									
GaSn													0.01					0.02	0.05	0.08	0.06		0.06	0.07	0.08		0.05	0.01	0.02	0.10	0.05	0.02				0.05					
GaSl																				0.01			0.01	0.01	0.01		0.01			0.01	0.01					0.01					

Supplementary references

1. Frostegard, A. & Bååth, E. The use of phospholipid fatty acid analysis to estimate bacterial and fungal biomass in soil. *Biol. Fertil. Soils* **22**, 59–65 (1996).
2. Prada-Salcedo, L. D., Wambsganss, J., Bauhus, J., Buscot, F. & Goldmann, K. Low root functional dispersion enhances functionality of plant growth by influencing bacterial activities in European forest soils. *Environ. Microbiol.* **23**, 1889–1906 (2021).
3. Bligh, E. G. & Dyer, W. J. A rapid method of total lipid extraction and purification. *Can. J. Biochem. Physiol.* **37**, 911–917 (1959).
4. Joergensen, R. G. Phospholipid fatty acids in soil—drawbacks and future prospects. *Biol. Fertil. Soils* **58**, 1–6 (2022).
5. Olsson, P. A., Bååth, E., Jakobsen, I. & Söderström, B. The use of phospholipid and neutral lipid fatty acids to estimate biomass of arbuscular mycorrhizal fungi in soil. *Mycol. Res.* **99**, 623–629 (1995).
6. Klamer, M. & Bååth, E. Estimation of conversion factors for fungal biomass determination in compost using ergosterol and PLFA 18:2ω6,9. *Soil Biol. Biochem.* **36**, 57–65 (2004).
7. Potapov, A. M. *et al.* Feeding habits and multifunctional classification of soil-associated consumers from protists to vertebrates. *Biol. Rev.* **97**, 1057–1117 (2022).
8. Waring, B. G., Averill, C. & Hawkes, C. V. Differences in fungal and bacterial physiology alter soil carbon and nitrogen cycling: insights from meta-analysis and theoretical models. *Ecol. Lett.* **16**, 887–894 (2013).
9. Fierer, N., Strickland, M. S., Liptzin, D., Bradford, M. A. & Cleveland, C. C. Global patterns in belowground communities. *Ecol. Lett.* **12**, 1238–1249 (2009).
10. Tedersoo, L. *et al.* Best practices in metabarcoding of fungi: From experimental design to results. *Mol. Ecol.* **31**, 2769–2795 (2022).
11. Prada-Salcedo, L. D. *et al.* Fungal guilds and soil functionality respond to tree community traits rather than to tree diversity in European forests. *Mol. Ecol.* **30**, 572–591 (2021).
12. Nilsson, R. H. *et al.* The UNITE database for molecular identification of fungi: handling dark taxa and parallel taxonomic classifications. *Nucleic Acids Res.* **47**, D259–D264 (2019).
13. Nguyen, N. H. *et al.* FUNGuild: An open annotation tool for parsing fungal community datasets by ecological guild. *Fungal Ecol.* **20**, 241–248 (2016).
14. Collado, E. *et al.* Divergent above- and below-ground responses of fungal functional groups to forest thinning. *Soil Biol. Biochem.* **150**, 108010 (2020).
15. Tedersoo, L. *et al.* Tree diversity and species identity effects on soil fungi, protists and animals are context dependent. *ISME J.* **10**, 346–362 (2016).
16. Hagenbo, A. *et al.* Variations in biomass of fungal guilds are primarily driven by factors related to soil conditions in Mediterranean Pinus pinaster forests. *Biol. Fertil. Soils* **58**, 487–501 (2022).
17. Cheeke, T. E., Phillips, R. P., Kuhn, A., Rosling, A. & Fransson, P. Variation in hyphal production rather than turnover regulates standing fungal biomass in temperate hardwood forests. *Ecology* **102**, e03260 (2021).
18. Ehnes, R. B., Rall, B. C. & Brose, U. Phylogenetic grouping, curvature and metabolic scaling in terrestrial invertebrates. *Ecol. Lett.* **14**, 993–1000 (2011).
19. Andrassy, I. Die rauminhalts-und gewichtsbestimmung der fadenwürmer (Nematoden). *Acta Zool. Hung.* **2**, 1–5 (1956).
20. Yeates, G. W. Soil nematodes in terrestrial ecosystems. *J. Nematol.* **11**, 213–229 (1979).
21. Yeates, G. W., Bongers, T., Degoede, R. G. M., Freckman, D. W. & Georgieva, S. S. Feeding Habits in Soil Nematode Families and Genera—An Outline for Soil Ecologists. *J. Nematol.* **25**, 315–331 (1993).

22. Tanaka, M. Ecological studies on communities of soil Collembola in Mt. Sobo, southwest Japan. *Jpn. J. Ecol.* **20**, 102–110 (1970).
23. Petersen, H. Estimation of dry weight, fresh weight, and calorific content of various Collembolan species. *Pedobiologia* **15**, 222–243 (1975).
24. Potapov, A. M. *et al.* Globally invariant metabolism but density-diversity mismatch in springtails. *Nat. Commun.* **14**, 674 (2023).
25. Petersen, H. & Luxton, M. A comparative analysis of soil fauna populations and their role in decomposition processes. *Oikos* **39**, 287–388 (1982).
26. Edwards, C. A. Relationship between weights, volumes and numbers of soil animals. in *Progress in Soil Biology* 585–591 (North-Holland Publishing Company, New York, 1967).
27. Ganault, P. *et al.* Relative importance of tree species richness, tree functional type, and microenvironment for soil macrofauna communities in European forests. *Oecologia* **196**, 455–468 (2021).
28. Mercer, R. D., Gabriel, A. G. A., Barendse, J., Marshall, D. J. & Chown, S. L. Invertebrate body sizes from Marion Island. *Antarct. Sci.* **13**, 135–143 (2001).
29. Gillespie, L. M. *et al.* Tree species mixing affects soil microbial functioning indirectly via root and litter traits and soil parameters in European forests. *Funct. Ecol.* **35**, 2190–2204 (2021).
30. Brown, J. H., Gillooly, J. F., Allen, A. P., Savage, V. M. & West, G. B. Toward a metabolic theory of ecology. *Ecology* **85**, 1771–1789 (2004).
31. Anderson, J. P. E. & Domsch, K. H. A physiological method for the quantitative measurement of microbial biomass in soils. *Soil Biol. Biochem.* **10**, 215–221 (1978).
32. Xu, X. *et al.* Global Pattern and Controls of Soil Microbial Metabolic Quotient. *Ecol. Monogr.* **87**, 429–441 (2017).
33. Sakamoto, K. & Oba, Y. Effect of fungal to bacterial biomass ratio on the relationship between CO2 evolution and total soil microbial biomass. *Biol. Fertil. Soils* **17**, 39–44 (1994).
34. Bååth, E. & Anderson, T. H. Comparison of soil fungal/bacterial ratios in a pH gradient using physiological and PLFA-based techniques. *Soil Biol. Biochem.* **35**, 955–963 (2003).
35. Sandler, S. I. & Orbey, H. On the thermodynamics of microbial growth processes. *Biotechnol. Bioeng.* **38**, 697–718 (1991).
36. Jochum, M. *et al.* Decreasing Stoichiometric Resource Quality Drives Compensatory Feeding across Trophic Levels in Tropical Litter Invertebrate Communities. *Am. Nat.* **190**, 131–143 (2017).
37. Lang, B., Ehnes, R. B., Brose, U. & Rall, B. C. Temperature and consumer type dependencies of energy flows in natural communities. *Oikos* **126**, 1717–1725 (2017).
38. Wambsganss, J., Beyer, F., Freschet, G. T., Scherer-Lorenzen, M. & Bauhus, J. Tree species mixing reduces biomass but increases length of absorptive fine roots in European forests. *J. Ecol.* **109**, 2678–2691 (2021).
39. Wambsganss, J. *et al.* Tree species mixing causes a shift in fine-root soil exploitation strategies across European forests. *Funct. Ecol.* **35**, 1886–1902 (2021).
40. McCormack, M. L. *et al.* Redefining fine roots improves understanding of below-ground contributions to terrestrial biosphere processes. *New Phytol.* **207**, 505–518 (2015).
41. Yuan, Z. Y., Chen, H. Y. H. & Reich, P. B. Global-scale latitudinal patterns of plant fine-root nitrogen and phosphorus. *Nat. Commun.* **2**, 344 (2011).
42. Dawud, S. M. *et al.* Is Tree Species Diversity or Species Identity the More Important Driver of Soil Carbon Stocks, C/N Ratio, and pH? *Ecosystems* **19**, 645–660 (2016).
43. Potapov, A. M. Multifunctionality of belowground food webs: resource, size and spatial energy channels. *Biol. Rev.* **97**, 1691–1711 (2022).

44. Barnes, A. D. *et al.* Consequences of tropical land use for multitrophic biodiversity and ecosystem functioning. *Nat. Commun.* **5**, 5351 (2014).
45. Yao, H., Chapman, S. J., Thornton, B. & Paterson, E. 13C PLFAs: a key to open the soil microbial black box? *Plant Soil* **392**, 3–15 (2015).
46. Högberg, P. *et al.* High temporal resolution tracing of photosynthate carbon from the tree canopy to forest soil microorganisms. *New Phytol.* **177**, 220–228 (2008).
47. Kaiser, C. *et al.* Exploring the transfer of recent plant photosynthates to soil microbes: mycorrhizal pathway vs direct root exudation. *New Phytol.* **205**, 1537–1551 (2015).
48. Kramer, C. & Gleixner, G. Soil organic matter in soil depth profiles: Distinct carbon preferences of microbial groups during carbon transformation. *Soil Biol. Biochem.* **40**, 425–433 (2008).
49. Treonis, A. M. *et al.* Identification of groups of metabolically-active rhizosphere microorganisms by stable isotope probing of PLFAs. *Soil Biol. Biochem.* **36**, 533–537 (2004).
50. Ling, N., Wang, T. & Kuzyakov, Y. Rhizosphere bacteriome structure and functions. *Nat. Commun.* **13**, 836 (2022).
51. Lu, W. *et al.* Impact of vegetation community on litter decomposition: Evidence from a reciprocal transplant study with 13C labeled plant litter. *Soil Biol. Biochem.* **112**, 248–257 (2017).
52. Lyu, M. *et al.* Simulated leaf litter addition causes opposite priming effects on natural forest and plantation soils. *Biol. Fertil. Soils* **54**, 925–934 (2018).
53. Shahzad, T. *et al.* Contribution of exudates, arbuscular mycorrhizal fungi and litter depositions to the rhizosphere priming effect induced by grassland species. *Soil Biol. Biochem.* **80**, 146–155 (2015).
54. Paterson, E., Sim, A., Osborne, S. M. & Murray, P. J. Long-term exclusion of plant-inputs to soil reduces the functional capacity of microbial communities to mineralise recalcitrant root-derived carbon sources. *Soil Biol. Biochem.* **43**, 1873–1880 (2011).
55. Brose, U. *et al.* Predator traits determine food-web architecture across ecosystems. *Nat. Ecol. Evol.* **3**, 919–927 (2019).
56. Barnes, A. D. *et al.* Energy Flux: The Link between Multitrophic Biodiversity and Ecosystem Functioning. *Trends Ecol. Evol.* **33**, 186–197 (2018).
57. Gauzens, B. *et al.* fluxweb: An R package to easily estimate energy fluxes in food webs. *Methods Ecol. Evol.* **10**, 270–279 (2019).
58. Manning, P. *et al.* Redefining ecosystem multifunctionality. *Nat. Ecol. Evol.* **2**, 427–436 (2018).
59. Byrnes, J. E. K. *et al.* Investigating the relationship between biodiversity and ecosystem multifunctionality: challenges and solutions. *Methods Ecol. Evol.* **5**, 111–124 (2014).
60. Byrnes, J. multifunc: Analysis of ecological drivers on ecosystem multifunctionality R package version 0.6. 2. *R Found. Stat. Comput. Vienna* (2014).
61. Pérez-Harguindeguy, N. *et al.* New handbook for standardised measurement of plant functional traits worldwide. *Aust. J. Bot.* **61**, 167–234 (2013).
62. Kattge, J. *et al.* TRY plant trait database – enhanced coverage and open access. *Glob. Change Biol.* **26**, 119–188 (2020).
63. Laliberté, E. & Legendre, P. A distance-based framework for measuring functional diversity from multiple traits. *Ecology* **91**, 299–305 (2010).
64. Garnier, E. *et al.* Plant functional markers capture ecosystem properties during secondary succession. *Ecology* **85**, 2630–2637 (2004).
65. Revelle, W. *Psych: Procedures for Psychological, Psychometric, and Personality Research.* (2020).

66. Da, R., Fan, C., Zhang, C., Zhao, X. & von Gadow, K. Are absorptive root traits good predictors of ecosystem functioning? A test in a natural temperate forest. *New Phytol.* **239**, 75–86 (2023).
67. Jucker, T., Bouriaud, O., Avacaritei, D. & Coomes, D. A. Stabilizing effects of diversity on aboveground wood production in forest ecosystems: linking patterns and processes. *Ecol. Lett.* **17**, 1560–1569 (2014).
68. Joly, F.-X. *et al.* Tree species diversity affects decomposition through modified micro-environmental conditions across European forests. *New Phytol.* **214**, 1281–1293 (2017).
69. Ampoorter, E. *et al.* Driving mechanisms of overstorey–understorey diversity relationships in European forests. *Perspect. Plant Ecol. Evol. Syst.* **19**, 21–29 (2016).
70. Fick, S. E. & Hijmans, R. J. WorldClim 2: new 1-km spatial resolution climate surfaces for global land areas. *Int. J. Climatol.* **37**, 4302–4315 (2017).
71. Yang, X. *et al.* Determination of Soil Texture by Laser Diffraction Method. *Soil Sci. Soc. Am. J.* **79**, 1556–1566 (2015).
72. Weigelt, A. *et al.* An integrated framework of plant form and function: the belowground perspective. *New Phytol.* **232**, 42–59 (2021).
73. Wild, J. *et al.* Climate at ecologically relevant scales: A new temperature and soil moisture logger for long-term microclimate measurement. *Agric. For. Meteorol.* **268**, 40–47 (2019).
74. Josse, J., Chavent, M., Liquet, B. & Husson, F. Handling missing values with regularized iterative multiple correspondence analysis. *J. Classif.* **29**, 91–116 (2012).
75. Husson, F. & Josse, J. missMDA: Handling missing values with/in multivariate data analysis (principal component methods). (2014).
76. Van Soest, P. J. & Wine, R. H. Use of detergents in the analysis of fibrous feeds. IV. Determination of plant cell-wall constituents. *J. Assoc. Off. Anal. Chem.* **50**, 50–55 (1967).
77. Gessner, M. O. & Steiner, D. Acid Butanol Assay for Proanthocyanidins (Condensed Tannins). in *Methods to Study Litter Decomposition : A Practical Guide* (eds Graça, M. A. S., Bärlocher, F. & Gessner, M. O.) 107–114 (Springer, Netherlands, 2005).
78. Cornelissen, J. H. C. *et al.* Foliar pH as a new plant trait: can it explain variation in foliar chemistry and carbon cycling processes among subarctic plant species and types? *Oecologia* **147**, 315–326 (2006).
79. Freschet, G. T., Aerts, R. & Cornelissen, J. H. C. A plant economics spectrum of litter decomposability. *Funct. Ecol.* **26**, 56–65 (2012).
80. Joly, F.-X., Scherer-Lorenzen, M. & Hättenschwiler, S. Resolving the intricate role of climate in litter decomposition. *Nat. Ecol. Evol.* **7**, 214–223 (2023).
81. R Core Team. *R: A Language and Environment for Statistical Computing*. (R Foundation for Statistical Computing, Vienna, Austria, 2022).
82. Oksanen, J. *et al.* vegan: Community Ecology Package. R package version 2.6-4. (2022).
83. Laliberté, E., Legendre, P. & Shipley, Bill. FD: measuring functional diversity from multiple traits, and other tools for functional ecology. (2014).
84. Goodrich, B., Gabry, J., Ali, I. & Brilleman, S. rstanarm: Bayesian applied regression modeling via Stan. R package version 2.21.4. (2023).
85. Vehtari, A. *et al.* LOO: Efficient leave-one-out cross-validation and WAIC for Bayesian models (2019). *R Package Version* **2**, (2022).
86. Gronau, Q. F., Singmann, H. & Wagenmakers, E.-J. bridgesampling: An R package for estimating normalizing constants. *ArXiv Prepr. ArXiv171008162* (2017).
87. Lüdecke, D., Ben-Shachar, M. S., Patil, I., Waggoner, P. & Makowski, D. performance: An R package for assessment, comparison and testing of statistical models. *J. Open Source Softw.* **6**, (2021).

88. Gabry, J. & Mahr, T. bayesplot: Plotting for Bayesian models. *R Package Version* **1**, (2021).
89. Lefcheck, J. S. piecewiseSEM: Piecewise structural equation modeling in R for ecology, evolution, and systematics. *Methods Ecol. Evol.* **7**, 573–579 (2016).
90. Bates, D., Mächler, M., Bolker, B. & Walker, S. Fitting Linear Mixed-Effects Models Using lme4. *J. Stat. Softw. Vol 1 Issue 1 2015* https://doi.org/10.18637/jss.v067.i01 (2015) doi:10.18637/jss.v067.i01.
91. Gelman, A. *et al. Bayesian Data Analysis*. (CRC Press, 2013).
92. Zuur, A. F., Ieno, E. N. & Elphick, C. S. A protocol for data exploration to avoid common statistical problems. *Methods Ecol. Evol.* **1**, 3–14 (2010).
93. Ives, A. R. For testing the significance of regression coefficients, go ahead and log-transform count data. *Methods Ecol. Evol.* **6**, 828–835 (2015).
94. Schulz, T., Saastamoinen, M. & Vanhatalo, J. Model-based variance partitioning for statistical ecology. *Ecol. Monogr.* **95**, e1646 (2025).
95. Oberpriller, J., de Souza Leite, M. & Pichler, M. Fixed or random? On the reliability of mixed-effects models for a small number of levels in grouping variables. *Ecol. Evol.* **12**, e9062 (2022).
96. Nakagawa, S. & Cuthill, I. C. Effect size, confidence interval and statistical significance: a practical guide for biologists. *Biol. Rev.* **82**, 591–605 (2007).
97. Lovakov, A. & Agadullina, E. R. Empirically derived guidelines for effect size interpretation in social psychology. *Eur. J. Soc. Psychol.* **51**, 485–504 (2021).
98. Vehtari, A., Gelman, A. & Gabry, J. Practical Bayesian model evaluation using leave-one-out cross-validation and WAIC. *Stat. Comput.* **27**, 1413–1432 (2017).
99. Shi, H. & Yin, G. Reconnecting p-Value and Posterior Probability Under One- and Two-Sided Tests. *Am. Stat.* **75**, 265–275 (2021).
100. Shipley, B. Confirmatory path analysis in a generalized multilevel context. *Ecology* **90**, 363–368 (2009).
101. Cordlandwehr, V. *et al.* Do plant traits retrieved from a database accurately predict on-site measurements? *J. Ecol.* **101**, 662–670 (2013).
102. Barr, D. J., Levy, R., Scheepers, C. & Tily, H. J. Random effects structure for confirmatory hypothesis testing: Keep it maximal. *J. Mem. Lang.* **68**, 255–278 (2013).
103. Schielzeth, H. & Forstmeier, W. Conclusions beyond support: overconfident estimates in mixed models. *Behav. Ecol.* **20**, 416–420 (2009).
104. Matuschek, H., Kliegl, R., Vasishth, S., Baayen, H. & Bates, D. Balancing Type I error and power in linear mixed models. *J. Mem. Lang.* **94**, 305–315 (2017).
105. Byrnes, J. E. K. & Dee, L. E. Causal Inference With Observational Data and Unobserved Confounding Variables. *Ecol. Lett.* **28**, e70023 (2025).
106. Joly, F.-X. *et al.* Detritivore conversion of litter into faeces accelerates organic matter turnover. *Commun. Biol.* **3**, 660 (2020).
107. Ponge, J. F. Humus forms in terrestrial ecosystems: a framework to biodiversity. *Soil Biol. Biochem.* **35**, 935–945 (2003).
108. Zanella, A. *et al.* A European morpho-functional classification of humus forms. *Geoderma* **164**, 138–145 (2011).
109. Schwarz, B. *et al.* Warming alters energetic structure and function but not resilience of soil food webs. *Nat. Clim. Change* **7**, 895–900 (2017).
110. Wan, B. *et al.* Energy flux across multitrophic levels drives ecosystem multifunctionality: Evidence from nematode food webs. *Soil Biol. Biochem.* **169**, 108656 (2022).
111. Schielzeth, H. & Nakagawa, S. Nested by design: model fitting and interpretation in a mixed model era. *Methods Ecol. Evol.* **4**, 14–24 (2013).

112. Loreau, M. Biodiversity and ecosystem functioning: recent theoretical advances. *Oikos* **91**, 3–17 (2000).
113. Loreau, M. *et al.* Biodiversity and ecosystem functioning: Current knowledge and future challenges. *Science* **294**, 804–808 (2001).
114. Barry, K. E. *et al.* The Future of Complementarity: Disentangling Causes from Consequences. *Trends Ecol. Evol.* **34**, 167–180 (2019).
115. Hooper, D. U. *et al.* Effects of biodiversity on ecosystem functioning: A consensus of current knowledge. *Ecol. Monogr.* **75**, 3–35 (2005).
116. Díaz, S. *et al.* Incorporating plant functional diversity effects in ecosystem service assessments. *Proc. Natl. Acad. Sci.* **104**, 20684–20689 (2007).
117. Grime, J. P. Benefits of plant diversity to ecosystems: immediate, filter and founder effects. *J. Ecol.* **86**, 902–910 (1998).
118. Wardle, D. A. *et al.* Ecological linkages between aboveground and belowground biota. *Science* **304**, 1629–1633 (2004).
119. Wardle, D. A. & Lavelle, P. Linkages between soil biota, plant litter quality and decomposition. in *Driven by Nature – Plant Litter Quality and Decomposition* (eds Cadisch, G. & Giller, K. E.) 107–127 (CAB International, Wallingford, 1997).
120. Zhang, Y., Chen, H. Y. H. & Reich, P. B. Forest productivity increases with evenness, species richness and trait variation: a global meta-analysis. *J. Ecol.* **100**, 742–749 (2012).
121. Henneron, L. *et al.* Forest management adaptation to climate change – A Cornelian dilemma between drought resistance and soil macro-detritivore functional diversity. *J. Appl. Ecol.* **52**, 913–927 (2015).
122. Henneron, L. *et al.* Forest plant community as a driver of soil biodiversity: experimental evidence from collembolan assemblages through large-scale and long-term removal of oak canopy trees Quercus petraea. *Oikos* **126**, 420–434 (2017).
123. Cebrian, J. Patterns in the fate of production in plant communities. *Am. Nat.* **154**, 449–468 (1999).
124. Pausch, J. & Kuzyakov, Y. Carbon input by roots into the soil: Quantification of rhizodeposition from root to ecosystem scale. *Glob. Change Biol.* **24**, 1–12 (2018).
125. Wardle, D. A., Yeates, G. W., Barker, G. M. & Bonner, K. I. The influence of plant litter diversity on decomposer abundance and diversity. *Soil Biol. Biochem.* **38**, 1052–1062 (2006).
126. Wardle, D. A. & Van der Putten, W. H. Biodiversity, ecosystem functioning and above-ground-below-ground linkages. in *Biodiversity and ecosystem functioning: synthesis and perspectives* (eds Loreau, M., Naeem, S. & Inchausti, P.) 155–168 (Oxford University Press, Oxford, 2002).
127. Hattenschwiler, S., Tiunov, A. V. & Scheu, S. Biodiversity and litter decomposition in terrestrial ecosystems. in *Annual Review of Ecology Evolution and Systematics* vol. 36 191–218 (Annual Reviews, Palo Alto, 2005).
128. Gilliam, F. S. The Ecological Significance of the Herbaceous Layer in Temperate Forest Ecosystems. *Bioscience* **57**, 845–858 (2007).
129. Nilsson, M. C. & Wardle, D. A. Understory vegetation as a forest ecosystem driver: evidence from the northern Swedish boreal forest. *Front. Ecol. Environ.* **3**, 421–428 (2005).
130. Augusto, L. *et al.* Influences of evergreen gymnosperm and deciduous angiosperm tree species on the functioning of temperate and boreal forests. *Biol. Rev.* **90**, 444–466 (2015).
131. Reich, P. B. *et al.* Linking litter calcium, earthworms and soil properties: a common garden test with 14 tree species. *Ecol. Lett.* **8**, 811–818 (2005).
132. Gillerot, L. *et al.* Forest structure and composition alleviate human thermal stress. *Glob. Change Biol.* **28**, 7340–7352 (2022).

133. Martin-Guay, M.-O. *et al.* Tree identity and diversity directly affect soil moisture and temperature but not soil carbon ten years after planting. *Ecol. Evol.* **12**, e8509 (2022).
134. Schnabel, F. *et al.* Tree Diversity Increases Forest Temperature Buffering via Enhancing Canopy Density and Structural Diversity. *Ecol. Lett.* **28**, e70096 (2025).
135. Beugnon, R. *et al.* Microclimate modulation: An overlooked mechanism influencing the impact of plant diversity on ecosystem functioning. *Glob. Change Biol.* **30**, e17214 (2024).
136. Ehnes, R. B., Rall, B. C. & Brose, U. Phylogenetic grouping, curvature and metabolic scaling in terrestrial invertebrates. *Ecol. Lett.* **14**, 993–1000 (2011).
137. Gan, H. & Wickings, K. Root herbivory and soil carbon cycling: Shedding "green" light onto a "brown" world. *Soil Biol. Biochem.* **150**, 107972 (2020).
138. Harrison, X. A. *et al.* A brief introduction to mixed effects modelling and multi-model inference in ecology. *PeerJ* **6**, e4794 (2018).
