# Supplementary material for: Tree community resource economics control soil food web multifunctionality
Source: Nature. 2026 May 6;655(8121):133–40. doi: 10.1038/s41586-026-10455-1 (PMC13322958; doi:10.1038/s41586-026-10455-1)
Supplement: Supplementary file 2 — Reporting Summary [file 41586_2026_10455_MOESM2_ESM.pdf]

nature portfolio

Corresponding author(s): Ludovic Henneron

Last updated by author(s): 16/03/2026

Reporting Summary

Nature Portfolio wishes to improve the reproducibility of the work that we publish. This form provides structure for consistency and transparency in reporting. For further information on Nature Portfolio policies, see our Editorial Policies and the Editorial Policy Checklist.

Statistics

For all statistical analyses, confirm that the following items are present in the figure legend, table legend, main text, or Methods section.

n/a	Confirmed	
☐	☒	The exact sample size (*n*) for each experimental group/condition, given as a discrete number and unit of measurement
☐	☒	A statement on whether measurements were taken from distinct samples or whether the same sample was measured repeatedly
☐	☒	The statistical test(s) used AND whether they are one- or two-sided<br>*Only common tests should be described solely by name; describe more complex techniques in the Methods section.*
☐	☒	A description of all covariates tested
☐	☒	A description of any assumptions or corrections, such as tests of normality and adjustment for multiple comparisons
☐	☒	A full description of the statistical parameters including central tendency (e.g. means) or other basic estimates (e.g. regression coefficient) AND variation (e.g. standard deviation) or associated estimates of uncertainty (e.g. confidence intervals)
☐	☒	For null hypothesis testing, the test statistic (e.g. *F*, *t*, *r*) with confidence intervals, effect sizes, degrees of freedom and *P* value noted<br>*Give P values as exact values whenever suitable.*
☐	☒	For Bayesian analysis, information on the choice of priors and Markov chain Monte Carlo settings
☐	☒	For hierarchical and complex designs, identification of the appropriate level for tests and full reporting of outcomes
☐	☒	Estimates of effect sizes (e.g. Cohen's *d*, Pearson's *r*), indicating how they were calculated

Our web collection on statistics for biologists contains articles on many of the points above.

Software and code

Policy information about availability of computer code

Data collection	Soil metagenomic sequencing was performed using the metabarcoding amplicon pipeline DeltaMP (https://github.com/lentendu/DeltaMP). The OTUs were then assigned to trophic guilds using the FUNGuild tool (https://github.com/UMNFuN/FUNGuild).
Data analysis	Data analysis was performed using R Statistical Software (v4.3.0) with the Rstudio interface (2023.03.1+446). The following packages were used: readxl (v1.4.3), xlsx (v0.6.5), erer (v3.1), purrr (v1.0.1), Matrix (v1.5-4.1), FactoMineR (1.34), fluxweb (v0.2.0), vegan (v2.6-8), FD (v1.0-12.1), missMDA (1.11), rstanarm (v2.21.4), loo (v2.6.0), bridgesampling (v1.1-2), performance (v0.12.3), bayesplot (v1.10.0), piecewiseSEM (v2.3.0), lme4 (1.1-33), multifunc (v0.9.4), and dplyr (v1.1.2). All R scripts for computation of food-web energy fluxes and statistical analyses are accessible in the figshare repository, available at https://doi.org/10.6084/m9.figshare.31700881.

For manuscripts utilizing custom algorithms or software that are central to the research but not yet described in published literature, software must be made available to editors and reviewers. We strongly encourage code deposition in a community repository (e.g. GitHub). See the Nature Portfolio guidelines for submitting code & software for further information.

Data

Policy information about availability of data

All manuscripts must include a data availability statement. This statement should provide the following information, where applicable:

- Accession codes, unique identifiers, or web links for publicly available datasets
- A description of any restrictions on data availability
- For clinical datasets or third party data, please ensure that the statement adheres to our policy

All data generated or analysed during this study are accessible in the figshare repository, available at https://doi.org/10.6084/m9.figshare.31700881.
This study also used previously published data accessible in the following databases: FunDivEUROPE (https://data.botanik.uni-halle.de/fundiveurope), Nemaplex (http://nemaplex.ucdavis.edu), BETSI (https://portail.betsi.cnrs.fr), TRY Plant Trait Database (https://www.try-db.org), DriloBASE (http://taxo.drilobase.org), UNITE (https://unite.ut.ee), and WorldClim (https://www.worldclim.org).

Research involving human participants, their data, or biological material

Policy information about studies with human participants or human data. See also policy information about sex, gender (identity/presentation), and sexual orientation and race, ethnicity and racism.

Reporting on sex and gender	n/a
Reporting on race, ethnicity, or other socially relevant groupings	n/a
Population characteristics	n/a
Recruitment	n/a
Ethics oversight	n/a

Note that full information on the approval of the study protocol must also be provided in the manuscript.

Field-specific reporting

Please select the one below that is the best fit for your research. If you are not sure, read the appropriate sections before making your selection.

☐ Life sciences ☐ Behavioural & social sciences ☒ Ecological, evolutionary & environmental sciences

For a reference copy of the document with all sections, see nature.com/documents/nr-reporting-summary-flat.pdf

Ecological, evolutionary & environmental sciences study design

All studies must disclose on these points even when the disclosure is negative.

Study description	The study investigates how the diversity and composition of naturally assembled tree communities influence multiple soil trophic functions across a range of environmental contexts in European forests. The study was conducted using a pan-European network of 64 mature forest plots (30 monospecific stands and 34 three-species mixture stands) distributed across four geographic locations in different countries (Finland, Poland, Romania, and Italy), spanning boreal to Mediterranean climates and representing highly contrasting European forest types
Research sample	The study sampled soil organisms, including microbes and faunal groups, including nematodes, microarthropods (springtails and mites), and macroinvertebrates (earthworms, gastropods, and macroarthopods).
Sampling strategy	Within each location, we followed a stratified sampling design by selecting three-species mixture stands with varying tree species compositions (n = 34 plots), along with corresponding monospecific stands (n= 30 plots). In each plot, we selected five 10 × 10 m subplots. Samples were taken equidistantly from three trees of either the same species in monospecific stands or of different species in three-species mixture stands. For each subplot, soil samples for microbial analyses and nematode extraction were collected by taking five soil cores (10 cm depth, 5.3 cm diameter), spaced approximately 35 cm apart around the equidistant point between the three trees, weighted by tree individual size (i.e. individual diameter at breast height). The five cores were gently sieved through 6 mm mesh (to avoid damaging nematodes), homogenized, and pooled at the subplot level for nematode extraction. The pooled soil was then sieved through 2 mm mesh for microbial analyses. For microarthropod extraction, an intact core (10 cm depth, 10 cm diameter), including both the litter and soil layers, was collected from each of three subplots along a southwest-northeast transect. For hand-sorting of soil macroinvertebrates, an intact monolith (25 cm depth, 25 × 25 cm surface), including both litter and soil layers, was collected from the same three subplots. No procedure was used to predetermine the sampling size. This was because no prior information about the size of tested effects was available. Sampling size was instead based on the trade-off between statistical power and feasibility in term of labor and cost involved.

Data collection	Authors of the paper applied a combination of collection methods to assess various groups of soil organisms, including microbes (phospholipid fatty acids extraction and analysis, metagenomic amplicon sequencing and bioinformatics), nematodes (sugar flotation extraction), microarthopods (Berlese-Tullgren funnel extraction) and macroinvertebrates (hand-sorting).
Timing and spatial scale	Soil organisms were sampled in all plots during the phenological spring of 2017, a period of high soil biological activity. We sampled both the litter layer (unfragmented aboveground litter, OL horizon) and the soil layer (including both fragmented/humified organic matter and mineral soil, OF/OH/A horizons).
Data exclusions	No data were excluded from the analysis.
Reproducibility	As is typical for large-scale and ongoing ecological field experiments, the data presented arises from specific spatial and temporal ecological conditions that may change over time, making exact replication of the experiment inherently challenging. This underlines the importance of sampling across a wide range of conditions (e.g. multiple forest types, tree species and species combinations, climatic conditions, and soil types) such as was as done in this study. This approach allows us to identify general and potentially reproducible patterns rather than site-specific trends.
Randomization	Randomization was not relevant to our study because we were using an already established experimental setup.
Blinding	Blinding was not relevant to our study.

Did the study involve field work? ☒ Yes ☐ No

Field work, collection and transport

Field conditions	The sites used in this study are part of a permanent network of mature forest plots across Europe established in 2011-2012. We included four sites spanning a large climatic gradient: North Karelia, Finland (MAT 2.1°C; MAP 700 mm); Białowieża, Poland (MAT 6.9°C; MAP 627 mm); Râşca, Romania (MAT 6.8°C; MAP 800 mm); and Colline Metallifere, Italy (MAT 13°C; MAP 850 mm).
Location	The geographic coordinates of the sites are: North Karelia, Finland (latitude, 62.6°, longitude 29.9°); Białowieża, Poland (52.7, 23.9°); Râşca, Romania (47.3°, 26.0°); and Colline Metallifere, Italy (43.2°, 11.2°). These locations correspond to typical boreal forests, hemiboreal mixed broadleaved-coniferous, mountainous mixed beech, and Mediterranean thermophilous forests, respectively. Within each site, we selected 30 m × 30 m forest plots dominated by either one tree species (monospecific plots) or three tree species (mixture plots): North Karelia (6 monospecific plots, 3 mixture plots); Białowieża (6 monospecific stands, 14 mixture plots); Râşca (8 monospecific plots, 8 mixture plots); and Colline Metallifere (10 monospecific plots, 9 mixture plots).
Access & import/export	All plots were accessible by car or on foot, and we were accompanied by local scientists or guides hired by the local site management responsible for the permanent plots. Soil samples were collected within Europe, and no permits were required for transportation.
Disturbance	When accessing the plots, efforts were made to minimize disturbance by following established trails and avoiding unnecessary movement through the forest. Within the plots, disturbance of the forest floor litter was unavoidable; however, soil cores were refilled after sampling and the litter layer was replaced over the sampled area.

Reporting for specific materials, systems and methods

We require information from authors about some types of materials, experimental systems and methods used in many studies. Here, indicate whether each material, system or method listed is relevant to your study. If you are not sure if a list item applies to your research, read the appropriate section before selecting a response.

Materials & experimental systems

n/a	Involved in the study	
☒	☐	Antibodies
☒	☐	Eukaryotic cell lines
☒	☐	Palaeontology and archaeology
☐	☒	Animals and other organisms
☒	☐	Clinical data
☒	☐	Dual use research of concern
☐	☒	Plants

Methods

n/a	Involved in the study	
☒	☐	ChIP-seq
☒	☐	Flow cytometry
☒	☐	MRI-based neuroimaging

Animals and other research organisms

Policy information about studies involving animals; ARRIVE guidelines recommended for reporting animal research, and Sex and Gender in Research

Laboratory animals	This study did not involve laboratory animals.
Wild animals	Wild animals were not used in this study.
Reporting on sex	n/a
Field-collected samples	Only invertebrate animals (nematodes, arthropods, gastropods and earthworms) were collected and killed during sampling and extraction to assess biomass and community composition. Macroinvertebrates were hand-sorted in the field for each subplot and fixed in 70% ethanol. Microarthropods were extracted from intact cores (including both the litter and soil layers) within 72 h after sampling for each subplot using the Berlese-Tullgren funnel method and were fixed in 70% ethanol. Nematodes were extracted for each subplot within 72 h after sampling from approximately 100 g of fresh soil using a modified sugar flotation method, after which they were heat-killed and fixed in 4% formaldehyde.
Ethics oversight	No ethical approval or guidance was required. We did not work with dangerous nor foreign materials (i.e. exotic species, pathogens, etc.).

Note that full information on the approval of the study protocol must also be provided in the manuscript.

Dual use research of concern

Policy information about dual use research of concern

Hazards

Could the accidental, deliberate or reckless misuse of agents or technologies generated in the work, or the application of information presented in the manuscript, pose a threat to:

No	Yes	
☒	☐	Public health
☒	☐	National security
☒	☐	Crops and/or livestock
☒	☐	Ecosystems
☒	☐	Any other significant area

Experiments of concern

Does the work involve any of these experiments of concern:

No	Yes	
☒	☐	Demonstrate how to render a vaccine ineffective
☒	☐	Confer resistance to therapeutically useful antibiotics or antiviral agents
☒	☐	Enhance the virulence of a pathogen or render a nonpathogen virulent
☒	☐	Increase transmissibility of a pathogen
☒	☐	Alter the host range of a pathogen
☒	☐	Enable evasion of diagnostic/detection modalities
☒	☐	Enable the weaponization of a biological agent or toxin
☒	☐	Any other potentially harmful combination of experiments and agents

Plants

Seed stocks	n/a
Novel plant genotypes	n/a
Authentication	n/a
