# Supplementary material for: Tree community resource economics control soil food web multifunctionality
Source: Nature. 2026 May 6;655(8121):133–40. doi: 10.1038/s41586-026-10455-1 (PMC13322958; doi:10.1038/s41586-026-10455-1)
Supplement: Supplementary file 3 — Peer Review file [file 41586_2026_10455_MOESM3_ESM.pdf]

nature portfolio

Peer Review File

Tree community resource economics control soil food web multifunctionality

Corresponding Author: Dr Ludovic Henneron

Any redactions in this file are there to maintain patient confidentiality, the confidentiality of unpublished data, or to remove third-party material.

This file contains all reviewer reports in order by version, followed by all author rebuttals in order by version.

Version 0:

Reviewer comments:

Referee #1

(Remarks to the Author)
This manuscript presents an intriguing analysis of the influence of tree functional diversity on soil food web multifunctionality across a range of European forests. I would like to say that I have thoroughly enjoyed reading about this study, which has a range of interesting findings and valuable insights into the drivers of variability in soil food web energy fluxes. The manuscript shows clear evidence for the strong role of leaf and fine-root economic traits for driving variability in multiple single functions (or energy fluxes), as well as on combined multifunctionality metrics derived from consumer-resources energy fluxes. To my knowledge, no such study exists, whereby the influence of plant functional diversity on soil food web energy fluxes and derived ecosystem multifunctionality is investigated, especially at a large spatial scale (beyond typical local-scale grassland experiments). Furthermore, I have carefully reviewed the methodology applied to estimate energy fluxes (ranging from body mass calculations, to estimates of assimilation efficiencies, to food web reconstruction and estimation of energy fluxes), as well as the statistical analyses, all of which appear to be very carefully thought through and appropriately applied.

That said, while I was very intrigued by the aim of comparing the relative effects of plant taxonomic diversity (spp. richness) versus functional diversity on soil food web multifunctionality, the data derived from the FunDivEUROPE experiment frankly do not seem appropriate to test this question. This is because there is very limited scope to test for a taxonomic diversity effect, given the highly constrained variation in species richness in the experimental design. Specifically, I am highly skeptical that a comparison of plots with either 1 or 3 tree species is sufficient to characterize diversity effects based on species richness, especially when this is compared to functional diversity which of course has a far broader range as it is not specifically constrained by the number of species planted. The effects of taxonomic richness are limited to the specific context of 1 versus 3 species, which cannot really be fairly compared to the effects of functional diversity, that incorporates continuous variation across varying community composition (as well as richness). Thus, while I do not doubt the authors' argument that these richness levels are “typical for European forest ecosystems”, this still does not justify the broader conclusions that could be drawn from this comparison between taxonomic and functional diversity effects and rather brings into question whether these “typical” European forest ecosystems should, in fact, be used to investigate this comparison of diversity facets. Ultimately, I just do not see why the authors have decided to devote a large part of the manuscript to comparing the effects of tree species richness versus tree functional diversity on soil food web functioning. This detracts from the main findings of the study (with respect to the resource economic traits), but also has led to some overly bold claims about the low importance of species richness that are not sufficiently supported by the underlying dataset.

I also have a few specific comments that arose as I was reading through the manuscript:

Line 107: Line 107: This does not only reflect an expectation of fundamental energetic structuring of natural food webs (i.e., pyramid shaped trophic structure), but also simply the top-down methodology used with the food web energetics approach. In other words, fluxes must be greater at lower trophic levels simply because their gains must at least match their losses to their predators. Thus, I do not feel this is a particularly noteworthy pattern to highlight in the results.

Line 108: "As well as for microbes than for fauna" - This pattern is clearly reported here:

Schwarz, B., Barnes, A.D., Thakur, M.P., Brose, U., Ciobanu, M., Reich, P.B., et al. (2017). Warming alters energetic structure and function but not resilience of soil food webs. Nat. Clim. Chang., 7, 895–900.

Line 167 - 170: This conclusion is highly speculative as this is not necessarily attributable to increased consumer metabolic demands, but could rather be an emergent result of increased primary productivity and input of dead organic matter in warmer conditions, leading to bottom-up effects on secondary productivity of consumer populations (thus indirectly increasing consumer metabolic demands). It is also possible that warmer climates reduce consumer population densities if they are unable to meet their increased metabolic demands, which is highly dependent on concurrent changes in primary productivity. Nevertheless, there is again some support for the assertion made by the authors here in the study by Schwarz et al (2017) Nature Climate Change.

Line 173 - 174: Yes, but these BEF studies typically include a much larger range of plant diversity, rather than an extremely simple categorical comparison between monoculture and 3-species mixtures (see my comment above). Therefore, I do not think the dataset used in this present study is appropriate to make such bold claims about the lack of, or "surprising" negative effect of, plant species richness on multifunctionality. My recommendation would be to rather focus on functional diversity and even species composition, which this dataset appears to be far better suited to address.

(Remarks on code availability)

Referee #2

(Remarks to the Author)
Summary of the key results

This study investigates how tree community diversity and functional composition shape soil food web functioning across 64 forest plots spanning four European biogeographic regions. Using a trophic energy flux modelling approach, the authors show that species diversity has weak or even negative effects on soil multifunctionality. In contrast, functional composition, particularly traits related to leaf and root economics, explains a significant proportion of the observed variability. Forests dominated by resource-acquisitive species (characterized by fast growth and higher nutritional quality/nitrogen-rich foliage) exhibited enhanced soil trophic functioning, likely driven by higher litter quality and warmer microclimatic conditions. These findings reinforce the view that functional trait composition (and resource-economic traits), rather than species richness per se, is a key determinant of belowground ecosystem processes.

Originality and significance

This study presents a highly original and timely approach by modelling soil trophic energy fluxes in naturally assembled forest communities. This is both novel and necessary in the context of biodiversity and ecosystem functioning research. The geographic scope is impressive: 64 plots across four European countries covering an important biogeographic gradient from boreal to Mediterranean forests.
Data & methodology: validity of approach, quality of data, quality of presentation
I am impressed by the quality of the design. The fact that the authors selected the plots while controlling for confounding factors such as topography greatly improves the robustness of inference in a non-experimental setting. They include replicates of different three species mixture treatments with different three species combinations and include monocultures of the same species. This design plus their plot selection allows the study to approximate to a BEF experiment. While a true BEF experiment is not feasible in a natural setting the careful selection of plots allows this study to closely approximate one. I also appreciate the integration of both foliar and root traits into metrics of diversity and community-weighted means. This design allows a clear comparison between functional diversity and mass-ratio hypotheses. It adds a deeper mechanistic understanding about how diversity operates, contrasting the effects of functionally diverse communities in which complementary mechanisms are expected to dominate vs. the effects of the dominant species (higher abundances or biomass) and their traits/attributes.

Appropriate use of statistics and treatment of uncertainties:

The analytical framework is solid; I really appreciate using structural equation modelling which in my opinion are a great choice to disentangle complex relationships, including direct and indirect effects. While I'm not an expert in multilevel Bayesian models, I see the added value compared to more conventional mixed-effects approaches. That said, I am not in a position to fully assess this particular part of the manuscript.
Something that I couldn´t find in the manuscript (apologize if I am wrong) is a clear validation of the Bayesian LMMs. As I said, I am not familiar with Bayesian statistics. Coming from frequentist statistics, I always validate the models and assumptions and include weights when needed. Adding this diagnosis checks greatly improves the rigor of the statistical methods. Although a Bayesian approach does not depend on a maximum likelihood the conclusions of the model are still based on a parametric structure. In such a case, I guess that checking fundamental model's assumptions is essential to ensure reliability of the results. I am curious whether there is an analog to frequentist statistics to assess the models fit.

Suggested improvements: experiments, data for possible revision. Conclusions.

I detected some inconsistency in trait data sources. The authors mention that trait data were "mostly derived from in situ measured values". While N content was measured in all spp and plots, SLA and LDMC were measured in situ only in

Finland and Romania plots, implying that for some species and sites (Italy and Poland) they relied on database values (they used TRY). This mixing of in situ and external trait data can introduce biases, particularly in the calculation of community-weighted means (CWMs) and functional diversity metrics (FDis), as trait expression is context-dependent (e.g., varies with soil, light, or climate conditions). While this is reasonable, given the impressive geographic scope and complexity of the sampling design, it does introduce a degree of inconsistency that may affect the CWM and FDis metrics. Maybe a sensitivity analysis should be provided to assess whether results are robust to this variation in trait sources. If this is not feasible, at least a more explicit discussion of the limitations introduced by this inconsistency would be appropriate. This is just a minor comment in the context of the whole study.
Probably the most important comment I can make concerns some weakness in the comparison between diversity and composition effects. While my opinion and own experience is aligned with their findings and the idea that species composition tends to play a stronger role than diversity in driving ecosystem functioning, the strength of this conclusion in the present study might be limited/affected by the limited species richness gradient evaluated (the authors are comparing only monocultures to three-species mixtures). Such a narrow gradient constrains the ability to robustly assess the role of diversity and limits the generalizability of the conclusions regarding its relative importance. This limitation should be more explicitly acknowledged and discussed in the manuscript. All these make me think that the authors might be overinterpreting the absence of diversity effects.
The manuscript is highly complex and includes a strong technical component. This is not necessarily an issue, in fact the quantitative approach is powerful but the combination of extensive field data, strong analytical approach, sophisticated methods, detailed analytical procedures make the paper challenging to follow, particularly to readers that are not familiar with some of the parts. My expertise aligns well with the subject of the manuscript and still I found some parts of the methods and Suppl. Information difficult to follow (and difficult to assess with confidence), simply due to the depth and extend of the study. Additionally, the large number of extended figures and supplementary material tends to disrupt the narrative flow (while they are valuable).
This is not a fundamental flaw (on the contrary the technical complexity underpins the strength of this study). However, I think that the clarity of the study could benefit from the inclusion of an infographic or conceptual summary figure early in the main manuscript (something summarizing the data layers, design, analytical steps…)

Clarity and context, credit to previous work.

The manuscript is clearly written, concise and well structured. The abstract, introduction, approach and conclusions are appropriate and contextualized in the literature. The authors give credit to previous work including foundational references. Some references that align well with this study that could be checked and included if appropriate. The last one adds a theoretical perspective that aligns well with the approach of this work.
- Hu Z, Michaletz ST, Johnson DJ, McDowell NG, Huang Z, Zhou X, Xu C. Traits drive global wood decomposition rates more than climate. Glob Chang Biol. 2018 Nov;24(11):5259-5269. doi: 10.1111/gcb.14357. Epub 2018 Jul 3. PMID: 29901246. https://pubmed.ncbi.nlm.nih.gov/29901246/
- Michaletz, S. T., Kerkhoff, A. J., & Enquist, B. J. (2018). Drivers of terrestrial plant production across broad geographical gradients. Global Ecology and Biogeography, 27, 166–174. https://doi.org/10.1111/geb. 12685
- Chacón-Labella, J. Hinojo-Hinojo, C., Bohner, T., Castorena, M., Violle, C., Vandvik, V., Enquist, B.J. How to improve scaling from traits to ecosystem processes. Trends in Ecology & Evolution. Volume 38, Issue 3. 2023. 228-237. https://doi.org/10.1016/j.tree.2022.10.007

My only remark is somewhat the strong tone in some conclusions, particularly regarding the role of species diversity, which should be interpreted with more caution, given the narrow richness gradient used.

(Remarks on code availability)

Referee #3

(Remarks to the Author)
This paper compares stands of trees of different diversity and species composition at 4 European sites to examine soil food web structure. It finds that soil food webs are most strongly influenced by location, aspects of species composition and to a lesser degree diversity. It presents a large amount of data on an understudied important topic (soil food webs) and appears to have been well analysed and presented (in general). My feedback concerns aspects of the analysis and the written presentation, as follows:

- Methods: The methods should include equations for the statistical models (best in statistical notation but at least in the syntax of the software - defining the terms and operators). This includes clarifying the analysis in the next point.

- Analysis: One of the key results is that species composition effects (and biogeography/locations) are larger than the diversity effects. There are various ways to assess this and the analysis is complex enough that I was not totally clear on which way(s) you used. One way is to look at percent variation explained. However, the caveat of this is that, all else equal, terms with more Degrees of Freedom will generally explain more. Here you compare richness (1 DF) with composition (33 DF). In terms of percent of variation composition explains more - but it is also more complex. Another approach is using variance components, including a 'Bayesian multilevel ANOVA' as proposed by Gelman (2005: J. Royal Stats Soc.) and Gelman & Hill (2006/7 book). You cite an application of this approach in your Ref. 15 that looked at richness and composition and found they were of a similar magnitude. I don't think you apply the approach to your data but you easily

could (extract variance components from your model(s)). As I understand it the idea is that (based on niche theory) richness is a simply (in statistical terms - single DF) frequently collected and available proxy for greater ecological/functional/phylogenetic diversity (which the literature seems to show it often is). It would be interesting to do the equivalent analysis for your data and compare to the results of ref. 15 (and perhaps other applications in the literature, if there are any). If I misunderstood your analysis and you have already done this then see point 1 about the need for more explanation and inclusion of model formula (I know you provide code but not everyone will use the same software as you and readers should not have to go line by line through code to understand an analysis - I am just dealing with the same point for a paper of my own!).

- Analysis: Your (complex) analysis (see points above re. need for more info and models) attempts to deal with causation. Approaches for this are only now coming into Ecology (largely from econometrics - e.g. from Laura Dee, Jarret Byrnes and colleagues). Are you applying these methods in your analysis (if so which approaches exactly). You apply model selection but some recent advice suggests this is not appropriate for causal analyses (if I understand it model selection uncertainty means variables essential for getting at causation can be removed by model selection pathways). This is a very new and active area in Ecology (and generally). See Arif & MacNeil 2022 Ecology Letters & Laubach et al. 2021 PRSB.

- Analysis: Your data includes single-species and multispecies stands at the same location - at least for many combinations. This means you could use the single stand data to examine how the same species do in mixture. Do the species under/overyield. The general point here is that the performance of the trees in single and mixed species stands is an important part of understanding the diversity and composition effects - I suspect you could go further here than the current paper does and give greater understanding of why you see the effects that you do in terms of the response of the trees to the different stands.

- Intro/Discussion; Background/Interpretation: Following from the point on tree performance in the different stands - it would be good to compare the results from your 4-site study with the wider literature including meta-analyses. I am sure there will be more recent papers but one I have to hand is: Zhang, Y. et al (2012) Forest productivity increases with evenness, species richness and trait variation: a global meta- analysis. Journal of Ecology 100, 742–49 [doi: 10.1111/j.1365-2745.2011.01944.x].

- Interpretation/discussion: In some cases it seems Biogeography/location is the largest effect? To what degree are location and species composition correlated. The point here is that if environment determines species composition then is knowing location enough (detailed ecological data not needed?!)?

(Remarks on code availability)

Version 1:

Reviewer comments:

Referee #1

(Remarks to the Author)
I have carefully assessed the revised version of this manuscript and feel that the authors have comprehensively addressed my concerns on the initial version, as well as the concerns raised by other reviewers. I would like to commend the authors on this large effort to produce what I think is a very important and interesting study.

Referee #3

(Remarks to the Author)
TO AUTHORS

The revised MS is much improved, especially on clarity of the details of what has been done. However, I don't feel the edits to the text fully reflected changes - the abstract was relatively unchanged for example. A further round of minor revision could help the MS better reflect the changes made in review.

- One feature I think could use a little more discussion is the new result (in response to initial reviews) that the trees overyield, despite (or alongside) the soil community results (I think readers would not have expected that). I'm not sure how far you can go in explaining the tree overyielding result in relation to the soil community results but I think it is an intriguing result that suggests some complex and interesting ecology that future studies might want to investigate further.

- This is a good example of my general comment that the text could do with a final edit to better reflect some of the changes made in response to review:

"Overall, our results sharply contrast with most of the studies on species mixing effects, including biodiversity-ecosystem functioning experiments, which typically rely on randomly assembled communities and report positive effects of diversity on ecosystem functions especially for plant diversity"

This doesn't seem to take into account the overyielding result which I think you characterise as in line with previous results?! This suggests a more nuanced view where mixing can lead to overyielding but alongside the reported below-ground results? This and similar sections need some editing for a bit more nuance especially with regard to changes made during revision. This seems in line with some of the comments by the original review 1 on the original text.

- Comparing variable importance with variances vs. variance components
[I apologize for this rather 'techy' point but I think it is important (and interesting from a philosophy of science perspective) as quantifying the 'importance' of variables is not as simple as we often assume.]
Thanks for clarifying where variances have been used vs. variance components. This is a difficulty of the literature as the terms are often used inconsistently (especially in abbreviated labels in software) when the details of which is used can matter for interpretation. Statistically, it is only in the case of the residual error variance that variance and variance components are the same; for all other terms the variance is composed of the error variance / variance component plus one or more other variance components (the Rails data set example in the Pinheiro and Bates book is one simple example of how variances are made up of their constituent variance components). Whether variables are being compared in terms of the variances or variances components can matter as, all else equal, more complex terms (as reflected in higher numbers of DFs) would be expected to explain more. Simple terms (as quantified statistically in their DFs may explain less, but at a lesser cost and hence the advantage of simplicity (Occam's razor). Variance components are one (fundamental) measure that does not have the same issue of DFs (although no doubt has its own limits etc.). The point here is that, for clarity, I think it is better to use the term 'variance' when referring to variances in the classical mean squares (e.g. the numbers given in the table of variances in ANOVA tables - MS = SS divided by DFs) and variance components for variance components. At a few places in the MS you compare the percent variance explained by different terms - clarify if this is classical variances (e.g. add up all the variances in the ANOVA table and express each as a percentage of that total ~ percent total deviance; ~ R2) or whether the numbers are percentage of the summed variance components (i.e. add up the variance components and express each as a proportion/percent of the summed total - the example I use of this distinction when teaching is the kiwifruit example from Maindonald and Braun Data Analysis and Graphics and the associated DAAG R package) where it is thoroughly explained.

Version 2:

Reviewer comments:

Referee #3

(Remarks to the Author)
Thank you for your revised version – I have only one final small suggestion:

“This enabled us to contrast patterns consistent with the ‘niche complementarity’ hypothesis, which states that functionally more diverse communities promote ecosystem functioning through greater resource use complementarity”

It is true that early complementarity work focused on resource use, and there is evidence for this, but in some cases the complementarity comes via other mechanisms, including natural enemies (reduced pests and diseases in mixtures), facilitation (N fixation, hydraulic lift, nurse plant effects etc.) so perhaps consider a final minor tweak to acknowledge this wide range of possible niche and complementarity effects (something like):

“…promote ecosystem functioning through complementarity species interactions, with patterns…”

Open Access This Peer Review File is licensed under a Creative Commons Attribution 4.0 International License, which permits use, sharing, adaptation, distribution and reproduction in any medium or format, as long as you give appropriate credit to the original author(s) and the source, provide a link to the Creative Commons license, and indicate if changes were made.
In cases where reviewers are anonymous, credit should be given to 'Anonymous Referee' and the source.
The images or other third party material in this Peer Review File are included in the article's Creative Commons license, unless indicated otherwise in a credit line to the material. If material is not included in the article's Creative Commons license and your intended use is not permitted by statutory regulation or exceeds the permitted use, you will need to obtain permission directly from the copyright holder.
To view a copy of this license, visit https://creativecommons.org/licenses/by/4.0/

RESPONSE LETTER

Manuscript Nature 2025-02-03133
Thank you for considering our work and for giving us the opportunity to revise our manuscript. We are also grateful to the three reviewers for their constructive and insightful comments, which have helped us improve the manuscript.

In this response letter, we address the comments and suggestions of all three reviewers point by point, referring to changes made in the revised manuscript (highlighted in blue font). In response to Reviewer 1's main concern, we have toned down interpretations related to species richness effects and instead shifted the focus toward comparisons between tree monocultures and mixed stands. Further, to address Reviewer 2's suggestion and clarify our study's conceptual framework, we have included a new conceptual illustration outlining the workflow of our study (see Extended Data Fig. 1). In response to Reviewer 3, we have clarified the statistical analyses and strengthened the justification for the causal inferences presented. Additionally, we have conducted two new sensitivity analyses, which are now described together with the two previous sensitivity analyses in a new section in the Methods.

Please note that the important changes are highlighted in red into the revised manuscript, including both the main text and Supplementary Information. As required, we also provide another version containing tracked changes to facilitate review. Line numbers mentioned in our responses refer to the version *without* tracked changes.

Sincerely yours,

The authors

REVIEWER COMMENTS
Reviewer #1 (Remarks to the Author):

Comment: This manuscript presents an intriguing analysis of the influence of tree functional diversity on soil food web multifunctionality across a range of European forests. I would like to say that I have thoroughly enjoyed reading about this study, which has a range of interesting findings and valuable insights into the drivers of variability in soil food web energy fluxes. The manuscript shows clear evidence for the strong role of leaf and fine-root economic traits for driving variability in multiple single functions (or energy fluxes), as well as on combined multifunctionality metrics derived from consumer-resources energy fluxes. To my knowledge, no such study exists, whereby the influence of plant functional diversity on soil food web energy fluxes and derived ecosystem multifunctionality is investigated, especially at a large spatial scale (beyond typical local-scale grassland experiments). Furthermore, I have carefully reviewed the methodology applied to estimate energy fluxes (ranging from body mass calculations, to estimates of assimilation efficiencies, to food web reconstruction and estimation of energy fluxes), as well as the statistical analyses, all of which appear to be very carefully thought through and appropriately applied.

Response: We thank the reviewer for their thorough evaluation of our study and particularly the part describing the methodology. We are glad to see that the reviewer appreciates our large-scale study design, statistical analyses, and our application of the food web energetics approach.

Comment: That said, while I was very intrigued by the aim of comparing the relative effects of plant taxonomic diversity (spp. richness) versus functional diversity on soil food web multifunctionality, the data derived from the FunDivEUROPE experiment frankly do not seem appropriate to test this question. This is because there is very limited scope to test for a taxonomic diversity effect, given the highly constrained variation in species richness in the

experimental design. Specifically, I am highly skeptical that a comparison of plots with either 1 or 3 tree species is sufficient to characterize diversity effects based on species richness, especially when this is compared to functional diversity which of course has a far broader range as it is not specifically constrained by the number of species planted. The effects of taxonomic richness are limited to the specific context of 1 versus 3 species, which cannot really be fairly compared to the effects of functional diversity, that incorporates continuous variation across varying community composition (as well as richness). Thus, while I do not doubt the authors' argument that these richness levels are "typical for European forest ecosystems", this still does not justify the broader conclusions that could be drawn from this comparison between taxonomic and functional diversity effects and rather brings into question whether these "typical" European forest ecosystems should, in fact, be used to investigate this comparison of diversity facets. Ultimately, I just do not see why the authors have decided to devote a large part of the manuscript to comparing the effects of tree species richness versus tree functional diversity on soil food web functioning. This detracts from the main findings of the study (with respect to the resource economic traits), but also has led to some overly bold claims about the low importance of species richness that are not sufficiently supported by the underlying dataset.

Response: We thank the reviewer for this thoughtful comment highlighting an important point about the scope of inference for tree diversity effects in our study. We fully agree that our design, which is based on contrasts between monocultures and three-species mixtures, does not allow for a comprehensive test of species richness effects across a broad diversity gradient. However, we would like to clarify that directly comparing the relative effects of species richness *versus* functional diversity was not the aim of our study. Instead, our goal was to test how tree community composition and diversity (monocultures *versus* mixed stands) influence soil food web multifunctionality using both taxonomic and functional (trait-based) approaches. While the effects of tree species mixing and functional diversity appeared similar in magnitude within their respective approaches, this observation was presented very briefly and was not the central focus of the manuscript. To clarify the aim of our study, we have now modified the following sentences in the Introduction so to better highlight the general objectives focusing on different aspects of plant community properties (beyond the common approach of most BEF studies that are only testing species richness effects) and to make it more obvious that our aim was not to directly compare the relative effects of species richness *versus* functional diversity:

> L. 55-58. "Biological communities are experiencing major changes across trophic levels and habitats globally as a result of climate change, land transformation and species invasions[10]. Ecosystem processes such as productivity, decomposition and biogeochemical cycling are shaped by multiple community-level properties, including diversity and composition in terms of both species and trait spectra[11–13]."
>
> L. 102-105. "Here, we investigated how different properties of naturally assembled tree communities, including their diversity (three-species mixture *versus* monospecific stands) and their composition in terms of taxonomic identity and trait values, influence multiple soil trophic functions across a variety of environmental contexts in European forests."

Further, we agree that the terminology used in the original submission was confusing in some parts of the manuscript as it was not always clear what "diversity" referred to. We have therefore now opted for a clearer terminology by referring to "tree species mixing effects" throughout the manuscript when referring to the three-tree species mixture *versus* monospecific stands contrast (*i.e.* the taxonomic diversity aspect of the study), while avoiding using the term "tree species richness effects". We have now also reserved the term "functional diversity" for referring to the diversity of tree trait values calculated based on a functional dispersion index.

Accordingly, we have now reformulated our hypotheses as follow:

> L. 105-108. "We hypothesized that: (1) tree species mixing, through associated increases in functional diversity, positively affect soil food web multifunctionality, and (2) tree species composition exerts a strong effect on soil food web multifunctionality, owing to the key role of the economic traits of dominant tree species."

Our mention of 'tree diversity effects' now remains only when presenting the general background and aim of this study.

To de-emphasize reference to tree diversity effects in the revised manuscript, we have also performed multiple changes in the main text. First, we agree that the narrow range of richness levels in our study precludes inferences about the shape of richness-function relationships or the broader generalization of richness effects beyond our single one *versus* three species contrast, which has now been clarified as follow:

> L. 207-210. "It is important to note, however, that the diversity contrast in our study was limited to comparisons between monocultures and three-species mixtures. Future research across a broader gradient of tree species richness would be needed to better characterize the shape of the relationship between tree diversity and soil food web multifunctionality."
>
> L. 219-221. "This relatively minor role of species mixing relative to species composition may partially reflect the limited range of tree species richness represented in our study."

We also acknowledge our finding may not generalize to other ecosystems with higher levels of plant diversity, which we now state explicitly:

> L. 210-214. "While the relatively low species diversity tested here reflects typical conditions in European forest stands at the spatial scale that we considered[49], it remains an open question how tree diversity influences soil food web multifunctionality in more diverse forests in other regions of the worlds, where the occurrence likelihood of species with rare functional attributes is greater."

We have also revised our conclusions to more accurately reflect this limitation:

> L. 289-292. "In contrast, tree species mixing (three species mixtures *versus* monocultures) and functional diversity had much smaller and, generally, negative effects reflecting a shift in energy channelling from fine roots to litter, alongside a cooling effect on forest microclimate."

We hope that clarifying terminology and highlighting the limitations of our study regarding the test of tree diversity effects make our message clearer and avoids the impression that we intended to quantify species richness effects more generally, which is not our goal and that our study including only single and three species stands is not designed to test.

I also have a few specific comments that arose as I was reading through the manuscript:

Comment: - Line 107: Line 107: This does not only reflect an expectation of fundamental energetic structuring of natural food webs (i.e., pyramid shaped trophic structure), but also simply the top-down methodology used with the food web energetics approach. In other words, fluxes must be greater at lower trophic levels simply because their gains must at least match their losses to their predators. Thus, I do not feel this is a particularly noteworthy pattern to highlight in the results.

Response: We agree and have removed this sentence accordingly.

Comment: - Line 108: "As well as for microbes than for fauna" - This pattern is clearly reported here:
Schwarz, B., Barnes, A.D., Thakur, M.P., Brose, U., Ciobanu, M., Reich, P.B., et al. (2017). Warming alters energetic structure and function but not resilience of soil food webs. Nat. Clim. Chang., 7, 895–900.

Response: Thank you for pointing us to this reference. In the end, we decided to remove this sentence, because it adds relatively little to the developed context and is not particularly novel (see for instance Yi *et al.* 2025 for a recent example). However, we now do cite Schwarz *et al.* (2017) in Line 192 where we discuss microclimate effects on soil food web functioning (see our response to the next comment).

Comment: - Line 167 - 170: This conclusion is highly speculative as this is not necessarily attributable to increased consumer metabolic demands, but could rather be an emergent result of increased primary productivity and input of dead organic matter in warmer conditions, leading to bottom-up effects on secondary productivity of consumer populations (thus indirectly increasing consumer metabolic demands). It is also possible that warmer climates reduce consumer population densities if they are unable to meet their increased metabolic demands, which is highly dependent on concurrent changes in primary productivity. Nevertheless, there is again some support for the assertion made by the authors here in the study by Schwarz et al (2017) Nature Climate Change.

Response: We thank the reviewer for this thoughtful comment. We agree that the observed relationship between warmer microclimates and increased consumer energy fluxes could potentially reflect multiple underlying mechanisms, including both increased metabolic demands and bottom-up effects of enhanced primary productivity and organic matter inputs. However, our results provide limited support for the latter explanation in the context of our system. If warmer microclimates primarily increased consumer energy fluxes throughout enhanced aboveground productivity or litter inputs, we would expect microclimate to positively influence tree aboveground biomass or litterfall, but the results of our SEM model do not support this pathway (Fig. 4a). We emphasize that our microclimate measurements were taken at the ground level, which may not fully capture canopy-level thermal conditions; this clarification has now been added to Line 186. We have also revised the manuscript to discuss the possibility of bottom-up effects resulting from changes in belowground productivity and organic matter inputs, as follows:

> L. 194-198. "An alternative explanation is that lower energy fluxes in mixed stands arise from bottom-up effects, *i.e.* cooler ground-level conditions may decrease belowground primary productivity and input of plant root litter, which in turn lead to a decline in secondary productivity and the metabolic demands of consumer populations. However, our data show that the effects of microclimate on soil food web multifunctionality occurred independently of root biomass (Fig. 4)."

In response to the suggestion that warmer climates could reduce consumer population densities if metabolic demands exceed energy availability, we note that if this was indeed the case then this outcome would have been reflected in our estimates, which are based on trophic guild biomasses and therefore integrate both population sizes and energetic activity. Specifically, if higher metabolic costs under warmer conditions limited consumer population, then we would expect negative effect of microclimate on energy fluxes. However, our results consistently showed positive effect of warmer ground-level microclimates on soil food web energy fluxes, suggesting that metabolic demands were being met, and possibly exceeded, under these conditions.

We added a reference to Schwarz *et al.* (2017) in Line 192 in relation to this point, thank you for bringing it to our attention.

Comment: - Line 173 - 174: Yes, but these BEF studies typically include a much larger range of plant diversity, rather than an extremely simple categorical comparison between monoculture and 3-species mixtures (see my comment above). Therefore, I do not think the dataset used in

this present study is appropriate to make such bold claims about the lack of, or "surprising" negative effect of, plant species richness on multifunctionality. My recommendation would be to rather focus on functional diversity and even species composition, which this dataset appears to be far better suited to address.

Response: We appreciate the reviewer's comment and fully agree that our study does not encompass the broad gradient of species richness that typically characterizes dedicated BEF experiments. As outlined in our response to the main comment above, we have revised our manuscript to clearly acknowledge this limitation (*e.g.* lines 207-214, lines 219-221, and lines 289-292), and we have de-emphasized any text that was previously focused on species richness effects.

In line with the reviewer's suggestion, we have now shifted the focus of the manuscript toward testing the effects of different aspects of plant community properties, for which our dataset is indeed better suited. We believe that retaining the comparison between monocultures and three-species mixtures remains valuable and of interest to a broad readership, particularly given that this contrast reflects the typical range of diversity found in temperate and boreal forests in Europe and elsewhere, where monoculture and low-diversity mixtures are dominant and widely used in forest management. However, we now refer to these as species mixing effects rather than species diversity effects.

Referee #2 (Remarks to the Author):

Summary of the key results
Comment: This study investigates how tree community diversity and functional composition shape soil food web functioning across 64 forest plots spanning four European biogeographic regions. Using a trophic energy flux modelling approach, the authors show that species diversity has weak or even negative effects on soil multifunctionality. In contrast, functional composition, particularly traits related to leaf and root economics, explains a significant proportion of the observed variability. Forests dominated by resource-acquisitive species (characterized by fast growth and higher nutritional quality/nitrogen-rich foliage) exhibited enhanced soil trophic functioning, likely driven by higher litter quality and warmer microclimatic conditions. These findings reinforce the view that functional trait composition (and resource-economic traits), rather than species richness per se, is a key determinant of belowground ecosystem processes.

Response: We thank the reviewer for their accurate summary of our study.

Originality and significance
Comment: This study presents a highly original and timely approach by modelling soil trophic energy fluxes in naturally assembled forest communities. This is both novel and necessary in the context of biodiversity and ecosystem functioning research. The geographic scope is impressive: 64 plots across four European countries covering an important biogeographic gradient from boreal to Mediterranean forests.

Response: We are happy to see that the reviewer appreciates the originality and novelty of our study.

Data & methodology: validity of approach, quality of data, quality of presentation
Comment: I am impressed by the quality of the design. The fact that the authors selected the plots while controlling for confounding factors such as topography greatly improves the robustness of inference in a non-experimental setting. They include replicates of different three species mixture treatments with different three species combinations and include monocultures of the same species. This design plus their plot selection allows the study to approximate to a BEF experiment. While a true BEF experiment is not feasible in a natural setting the careful

selection of plots allows this study to closely approximate one. I also appreciate the integration of both foliar and root traits into metrics of diversity and community-weighted means. This design allows a clear comparison between functional diversity and mass-ratio hypotheses. It adds a deeper mechanistic understanding about how diversity operates, contrasting the effects of functionally diverse communities in which complementary mechanisms are expected to dominate vs. the effects of the dominant species (higher abundances or biomass) and their traits/attributes.

Response: We are glad that the reviewer appreciates the quality of our study design.

Appropriate use of statistics and treatment of uncertainties:
Comment: The analytical framework is solid; I really appreciate using structural equation modelling which in my opinion are a great choice to disentangle complex relationships, including direct and indirect effects. While I'm not an expert in multilevel Bayesian models, I see the added value compared to more conventional mixed-effects approaches. That said, I am not in a position to fully assess this particular part of the manuscript. Something that I couldn´t find in the manuscript (apologize if I am wrong) is a clear validation of the Bayesian LMMs. As I said, I am not familiar with Bayesian statistics. Coming from frequentist statistics, I always validate the models and assumptions and include weights when needed. Adding this diagnosis checks greatly improves the rigor of the statistical methods. Although a Bayesian approach does not depend on a maximum likelihood the conclusions of the model are still based on a parametric structure. In such a case, I guess that checking fundamental model's assumptions is essential to ensure reliability of the results. I am curious whether there is an analog to frequentist statistics to assess the models fit.

Response: Although Bayesian and frequentist linear mixed-effects models use different algorithms for model fitting (*i.e.* Markov Chain Monte Carlo for Bayesian models *versus* Restricted maximum likelihood for frequentist models) and exhibit some differences in the interpretation of some outputs (for instance, a 95% credible interval represents the range within which the parameter lies with 95% probability, whereas a 95% confidence interval does not carry this probabilistic interpretation), both approaches still rely on the same underlying statistical assumptions. We carefully checked these assumptions by various graphical checks and statistical tests to ensure the robustness of our inferences. While this was previously explained in the Supplementary Methods, we agree that this is too important to omit from the main text. We have now explicitly addressed this point as follows:

> L. 599-601 in the main text. "…each model was checked for their compliance with statistical assumptions, including normality and independence of residuals, normality of random effects, homogeneity of variance, linearity of relationships, and absence of multicollinearity."

Suggested improvements: experiments, data for possible revision. Conclusions.
Comment: I detected some inconsistency in trait data sources. The authors mention that trait data were "mostly derived from in situ measured values". While N content was measured in all spp and plots, SLA and LDMC were measured in situ only in Finland and Romania plots, implying that for some species and sites (Italy and Poland) they relied on database values (they used TRY). This mixing of in situ and external trait data can introduce biases, particularly in the calculation of community-weighted means (CWMs) and functional diversity metrics (FDis), as trait expression is context-dependent (e.g., varies with soil, light, or climate conditions). While this is reasonable, given the impressive geographic scope and complexity of the sampling design, it does introduce a degree of inconsistency that may affect the CWM and FDis metrics. Maybe a sensitivity analysis should be provided to assess whether results are robust to this variation in trait sources. If this is not feasible, at least a more explicit discussion of the

limitations introduced by this inconsistency would be appropriate. This is just a minor comment in the context of the whole study.

Response: We thank the reviewer for their helpful suggestion. We agree that trait values retrieved from database could potentially introduce some bias when combined with field-measured trait values due to intraspecific trait variability. Following the reviewer's suggestion, we have therefore performed a sensitivity analysis as described below to test the impact of this uncertainty on our inferences:

> L. 663-674. "Second, we addressed the potential uncertainty arising from the use of SLA and LDMC traits data retrieved from the TRY database for tree species in Poland and Italy, which may have limitations relative to on-site measurements because of intraspecific trait variation[89]. To assess how this might have affected our inferences, we performed 1,000 simulations. In each simulation, we fitted a Bayesian LMM (as described in Equation 2) using functional metrics (FDis and the first two principal components of CWM traits) computed from trait data in which SLA and LDMC values for the plots in Poland and Italy were randomly varied by around 30% around their original values retrieved from the TRY database. This 30% value was chosen because the coefficients of variation of the trait values retrieved from the TRY database for each tree species present in Poland and Italy were on average 25.3 % for SLA and 28.4% for LDMC. For each simulation, we extracted the standardized partial slopes and p-values of each variable, and visually checked their distributions (Supplementary Fig. 6). We found that this uncertainty in trait values had minimal effect on our inferences regarding soil food web multifunctionality."

Comment: Probably the most important comment I can make concerns some weakness in the comparison between diversity and composition effects. While my opinion and own experience is aligned with their findings and the idea that species composition tends to play a stronger role than diversity in driving ecosystem functioning, the strength of this conclusion in the present study might be limited/affected by the limited species richness gradient evaluated (the authors are comparing only monocultures to three-species mixtures). Such a narrow gradient constrains the ability to robustly assess the role of diversity and limits the generalizability of the conclusions regarding its relative importance. This limitation should be more explicitly acknowledged and discussed in the manuscript. All these make me think that the authors might be overinterpreting the absence of diversity effects.

Response: We thank the reviewer for this constructive comment, which is in line with Reviewer 1's main comment. In response to these comments, we use now more explicit and accurate terminology to avoid potential misunderstandings about species richness throughout the manuscript and have modified the Introduction to more clearly indicate the study's main focus. We also now acknowledge in several places in the manuscript that our study is not designed in such a way as to enable broader assessment of species richness effects (*e.g.* lines 207-214, lines 219-221, and lines 289-292) and in dealing with Reviewer 1's comments on this point, we now focus on 'species mixing effects' rather than 'species diversity effects'. We do however maintain that the comparison of monocultures and three-species mixtures are ecologically relevant for temperate and boreal forests in Europe and elsewhere, where low-diversity stands dominate and where species mixing is a widely applied management practice. Thus, while our findings may not generalize to ecosystems with broader richness gradients, they do offer insights into the functional consequences of tree species mixing under realistic, management-relevant conditions. For additional context, please also see our response to the main comment of Reviewer 1.

Comment: The manuscript is highly complex and includes a strong technical component. This is not necessarily an issue, in fact the quantitative approach is powerful but the combination of

extensive field data, strong analytical approach, sophisticated methods, detailed analytical procedures make the paper challenging to follow, particularly to readers that are not familiar with some of the parts. My expertise aligns well with the subject of the manuscript and still I found some parts of the methods and Suppl. Information difficult to follow (and difficult to assess with confidence), simply due to the depth and extend of the study. Additionally, the large number of extended figures and supplementary material tends to disrupt the narrative flow (while they are valuable). This is not a fundamental flaw (on the contrary the technical complexity underpins the strength of this study). However, I think that the clarity of the study could benefit from the inclusion of an infographic or conceptual summary figure early in the main manuscript (something summarizing the data layers, design, analytical steps…)

Response: The depth of our quantitative analyses in assessing the effects of multiple components of tree diversity and composition, along with biogeography, based on both taxonomic and functional approaches, provides novel mechanistic insights but indeed also adds a level of complexity to our study. We really appreciate the suggestion of adding an infographic or summary figure. In response, we have now included a new conceptual summary figure showing the study workflow (Extended Data Fig. 1).

Clarity and context, credit to previous work.

Comment: The manuscript is clearly written, concise and well structured. The abstract, introduction, approach and conclusions are appropriate and contextualized in the literature. The authors give credit to previous work including foundational references. Some references that align well with this study that could be checked and included if appropriate. The last one adds a theoretical perspective that aligns well with the approach of this work.

- Hu Z, Michaletz ST, Johnson DJ, McDowell NG, Huang Z, Zhou X, Xu C. Traits drive global wood decomposition rates more than climate. Glob Chang Biol. 2018 Nov;24(11):5259-5269. doi: 10.1111/gcb.14357. Epub 2018 Jul 3. PMID: 29901246. https://pubmed.ncbi.nlm.nih.gov/29901246/

- Michaletz, S. T., Kerkhoff, A. J., & Enquist, B. J. (2018). Drivers of terrestrial plant production across broad geographical gradients. Global Ecology and Biogeography, 27, 166–174. https://doi.org/10.1111/geb. 12685

- Chacón-Labella, J. Hinojo-Hinojo, C., Bohner, T., Castorena, M., Violle, C., Vandvik, V., Enquist, B.J. How to improve scaling from traits to ecosystem processes. Trends in Ecology & Evolution. Volume 38, Issue 3. 2023. 228-237. https://doi.org/10.1016/j.tree.2022.10.007

Response: Thank you for pointing us to these important references. The Chacón-Labella *et al.* (2023) reference is indeed relevant to our discussion of the (trait-based) approach and has now been cited in line 143. The first reference (Hu *et al.* 2018) is also relevant as it emphasizes the role of plant economic traits in ecosystem functioning, an aspect that aligns closely with our study. We have therefore added a new sentence to acknowledge this work alongside other key studies on this topic:

> L. 246-248. "More broadly, this aligns with a growing body of evidence highlighting the importance of plant economic traits for terrestrial ecosystem functioning[9,50–53]"

Comment: My only remark is somewhat the strong tone in some conclusions, particularly regarding the role of species diversity, which should be interpreted with more caution, given the narrow richness gradient used.

Response: As discussed above in response to the comments from both this reviewer and Rev. #1's comments, we agree on this point and modified the text throughout the manuscript to more cautiously refer to species richness effects and what our study can contribute to this issue, *e.g.* lines 207-214, lines 219-221, and lines 289-292.

Referee #3 (Remarks to the Author):

Comment: This paper compares stands of trees of different diversity and species composition at 4 European sites to examine soil food web structure. It finds that soil food webs are most strongly influenced by location, aspects of species composition and to a lesser degree diversity. It presents a large amount of data on an understudied important topic (soil food webs) and appears to have been well analysed and presented (in general). My feedback concerns aspects of the analysis and the written presentation, as follows:

Response: We thanks the reviewer for their insightful comments, which have helped us to further improve the manuscript.

Comment: - Methods: The methods should include equations for the statistical models (best in statistical notation but at least in the syntax of the software - defining the terms and operators). This includes clarifying the analysis in the next point.

Response: We have now provided the required equations and have modified the text in the Methods, *i.e.* line 585, line 593, and line 597.

Comment: - Analysis: One of the key results is that species composition effects (and biogeography/locations) are larger than the diversity effects. There are various ways to assess this and the analysis is complex enough that I was not totally clear on which way(s) you used. One way is to look at percent variation explained. However, the caveat of this is that, all else equal, terms with more Degrees of Freedom will generally explain more. Here you compare richness (1 DF) with composition (33 DF). In terms of percent of variation composition explains more - but it is also more complex. Another approach is using variance components, including a 'Bayesian multilevel ANOVA' as proposed by Gelman (2005: J. Royal Stats Soc.) and Gelman & Hill (2006/7 book). You cite an application of this approach in your Ref. 15 that looked at richness and composition and found they were of a similar magnitude. I don't think you apply the approach to your data but you easily could (extract variance components from your model(s)). As I understand it the idea is that (based on niche theory) richness is a simply (in statistical terms - single DF) frequently collected and available proxy for greater ecological/functional/phylogenetic diversity (which the literature seems to show it often is). It would be interesting to do the equivalent analysis for your data and compare to the results of ref. 15 (and perhaps other applications in the literature, if there are any). If I misunderstood your analysis and you have already done this then see point 1 about the need for more explanation and inclusion of model formula (I know you provide code but not everyone will use the same software as you and readers should not have to go line by line through code to understand an analysis - I am just dealing with the same point for a paper of my own!).

Response: We thank the reviewer for this constructive comment. However, although we were perhaps not as clear as we should have been, we did actually perform variance partitioning using the method proposed based on variance components. As explained by Hector *et al.* (2011, previously ref. 15), this method is suitable for comparing the relative importance of different explanatory variables with contrasting degrees of freedom. We have now rewritten this section, providing more detailed explanation on the procedure used:

> L. 602-606. “For each model, we performed variance partitioning following a model-based approach based on variance components[98] to quantify the proportion of variance in the dependent variable explained by three groups of predictors: diversity (tree species richness or functional dispersion, FDis), composition (tree species composition or CWM trait PC1 and PC2), and biogeography (abiotic conditions PC1 and PC2, and location)[99].”
>
> L. 619-635 in the Supplementary Methods. “To perform variance partitioning, we adopted a model-based approach based on variance components following Schulz *et al.* (2025)[84]. This allowed us to quantify the proportion of variance in the dependent variable explained by

three groups of predictors: diversity (tree species richness or FDis), composition (tree species composition or CWM trait PCs 1 and 2), and biogeography (abiotic condition PCs 1 and 2, as well as location), along with the model residuals (ε). In this framework, a variance component refers to the variance associated with a given model component (a_j), which can either be fixed ($x_j\beta_j$) or random (δ_j or ε). Each model component is assigned to a single predictor groups (m), so the group-level effect can be formally expressed as $b_l = \sum_{j \in B_l} a_j$ where $l = 1, \dots, m$ and B_l is a set of model components associated with the lth group. The variance of a group-level model component is then $Var[b_l] = Var\left[\sum_{j \in B_l} a_j\right] = \sum_{j \in B_l} \sum_{j' \in B_l} Cov\left[a_j, a'_j\right]$. Using this, we computed the diagonal variance partitioning for each group as:

$$V_{b_l}^{diag} = \frac{Var[b_l]}{\sum_{j'=1}^{m} Var[b_{l'}]} \quad (15)$$

This so-called *diagonal variance partition* ensures that the different group-level variance components sum up to one. However, it also ignores the covariances in the denominator, and hence also does not indicate the presence of confounding related to covariance between group-level model components."

Comment: - Analysis: Your (complex) analysis (see points above re. need for more info and models) attempts to deal with causation. Approaches for this are only now coming into Ecology (largely from econometrics - e.g. from Laura Dee, Jarret Byrnes and colleagues). Are you applying these methods in your analysis (if so which approaches exactly). You apply model selection but some recent advice suggests this is not appropriate for causal analyses (if I understand it model selection uncertainty means variables essential for getting at causation can be removed by model selection pathways). This is a very new and active area in Ecology (and generally). See Arif & MacNeil 2022 Ecology Letters & Laubach et al. 2021 PRSB.

Response: We thank the reviewer for highlighting this rapidly evolving area in ecological research. Our statistical analyses were indeed designed with the goal of causal inference. We followed the principles of the 'structural causal model' framework, which emphasizes the use of causal diagrams to guide variable selection. This approach allows for the inclusion of appropriate confounders while avoiding the inclusion of colliders and mediators that could bias causal estimates. Importantly, this approach does not rely on model selection for identifying causal variable but is grounded in theoretical reasoning about the underlying causal structure. This has now been clarified in the revised text:

L. 572-577. "To ensure that the effects of tree communities were not biased by confounding factors, we also explicitly included abiotic covariates into the models, *i.e.* the first two PCA axes representing abiotic conditions (Supplementary Fig. 2a). This statistical adjustment allowed us to control for variation in abiotic conditions both among and within locations. Following the principle of the 'structural causal model' framework, controlling for confounding factors allows to satisfy the 'backdoor criterion' required for quantifying unbiased causal relationships from observational data[96,97]."

In the second step of statistical workflow, we used structural equation modeling (SEM) to test *a priori* hypotheses regarding multivariate causal pathways. This included direct and indirect effects of tree diversity and composition on soil food web multifunctionality, potentially mediated through plant traits and micro-environmental conditions (Grace and Irvine, 2020; Correia *et al.* 2025). Here, hypothesised causal relationships are represented using component linear mixed-effects models, each testing how specific causal variables affects a given response variable. These component models were then simplified by removing unsupported pathways based on model selection procedures. We acknowledge recent concerns suggesting that predictive approaches and associated model selection procedures may be inappropriate for

drawing causal conclusions (Arif et MacNeil, 2022), though we refer to Nichols and Cooch (2025) for counter-arguments. We fully agree that initiating model selection from a naïve model including a large number of covariates without consideration of their roles as causal variables, confounders, mediators, or colliders, can lead to biased causal inferences, especially due to collider bias. However, we argue model simplification remains useful for excluding unsupported causal pathways, provided that the initial model is carefully constructed to include only plausible causal variables (Grace *et al.* 2012; Grace *et al.* 2015; Nichols and Cooch, 2025).

To clarify our SEM approach, we have implemented the following changes in the revised manuscript:

> L. 634-646. "Following a local estimation approach[106], we tested hypothesized causal relationships by fitting component LMMs with random intercepts across locations using Bayesian modelling, along with the use of variance partitioning and effect size computation methods in a manner similar to those described above. For each component LMM, abiotic covariates (see above) were included to ensure that mediation effects were not biased by confounding factors[105], thereby satisfying the 'backdoor criterion'. The initial SEM model was then simplified by removing unsupported linkages[106,107] using information-theoretic methods to select the most parsimonious SEM model. For each component LMM within the initial SEM model, stepwise backward selection was performed by removing the predictor with the highest p-value, verifying at each step that model simplification improved goodness-of-fit using Pareto smoothed importance-sampling leave-one-out (PSIS-LOO) cross-validation[108] (Supplementary Methods). To test the goodness-of-fit of the selected SEM model, we used the Shipley's test of directional separation[109], which assesses whether any important causal pathways may be missing (Supplementary Methods)."

We have also simplified and improved the presentation of the causal diagram to better explain that it was established based on ecological theory and our *prior* knowledge of the system, as now shown in our new Supplementary Fig. 3.

Finally, we performed a sensitivity test to assess potential omitted variable bias, following the approach proposed by Byrnes and Dee (2025). Given the possibility of bias in mixed-effects model when random effects correlate with causal variable, we applied a 'Mundlak' correction to account for potential unmeasured location-level confounding:

> L. 685-700. "we checked whether omitted variable bias due to potential unmeasured confounding variables at location level could affect our inference[113]. In ecological studies, linear mixed-effect model (LMMs) are commonly used to account for unmeasured cluster-level variables by incorporating them into random effects. However, this approach can lead to biased causal inference when random effects are correlated with the causal variable of interest. This issue is particularly relevant for variables not directly controlled by the study design, as is the case in our functional approach. To address this, we applied a correlated random effects approach ('Mundlak' approach), which incorporates group-level means of the causal variables to control for unmeasured confounders at the cluster (location) level. Including these group means serves as a proxy for potential confounding variables. This allowed us to estimate contextual effects, i.e., the between-location effects, of the causal variable. If the contextual effect is close to zero, it suggests that a standard LMM is sufficient and that omitted variable bias is likely minimal. For each causal variable used in the functional approach, we therefore fitted a Bayesian LMM (similar to the one described in Equation 2), that additionally included the location-level mean of the causal variable ($\bar{x}_i$). For all three causal variables, we found that the effect estimates remained consistent, and that the contextual effects ($\beta_{contextual}$) were close to zero (Supplementary Table 9). This indicates that omitted variable bias was not a concern in our study, given that we also accounted for measured abiotic confounding variables."

Comment: - Analysis: Your data includes single-species and multispecies stands at the same location - at least for many combinations. This means you could use the single stand data to examine how the same species do in mixture. Do the species under/overyield. The general point here is that the performance of the trees in single and mixed species stands is an important part of understanding the diversity and composition effects - I suspect you could go further here than the current paper does and give greater understanding of why you see the effects that you do in terms of the response of the trees to the different stands.

Response: We thank the reviewer for this constructive comment. In response, we calculated the relative yield of each species by measuring its biomass in a multi-species community as a proportion of its biomass in monoculture, and the relative yield total (RYT) of a multi-species community as the sum of the relative yields of all component species (Hector, 1998). The relative yield total is now provided in the Extended Data Table 1. Briefly, these results show that "the mean RYT was 1.17 when taking equal weight for all countries, showing an overyielding of 17%", as now stated in the caption of Extended Data Table 1 (see lines 1120-1121).

Comment: - Intro/Discussion; Background/Interpretation: Following from the point on tree performance in the different stands - it would be good to compare the results from your 4-site study with the wider literature including meta-analyses. I am sure there will be more recent papers but one I have to hand is: Zhang, Y. et al (2012) Forest productivity increases with evenness, species richness and trait variation: a global meta- analysis. Journal of Ecology 100, 742–49 [doi: 10.1111/j.1365- 2745.2011.01944.x].

Response: Good suggestion. We have now added such a comparison based on a more recent meta-analysis, *i.e.* Feng *et al.* (2022), in the caption of Extended Data Table 1, see lines 1121-1123: "This is in line with previous studies showing that tree species mixing increase tree aboveground biomass at stand-level by 25.5%, mostly due to complementary effects[115]."

Comment: - Interpretation/discussion: In some cases it seems Biogeography/location is the largest effect? To what degree are location and species composition correlated. The point here is that if environment determines species composition then is knowing location enough (detailed ecological data not needed?!)?

Response: Thank you for raising this relevant point. Indeed, biogeography accounted for a substantial proportion of the variance in our models. This result reflects, in part, the high turnover in tree species composition across locations, leading to potential covariance between biogeography and functional composition. This can complicate the interpretation of variance partitioning results, particularly when attempting to isolate the independent contributions of each group of predictors. To address this issue, and to assess the extent to which variance components may be correlated, we computed three complementary variance partition metrics following Schulz *et al.* (2025) that help disentangle overlapping effects. These are now presented in the new Extended Data Table 2, and described in detail lines 614-620 in the main text and lines 635-651 in the Supplementary Methods.

These new analyses allowed us to clarify our results in the main text as follows:

> L. 228-239. "In line with the 'mass-ratio' hypothesis[32], the functional composition of tree communities corresponding to community-level economic traits explained an even larger proportion of the variance in soil food web multifunctionality than did species composition within geographic locations alone (36.7% versus 22.3%, Fig. 3b,d). This likely reflects the fact that functional composition captures both species turnover and intraspecific variability across geographic locations. Moreover, unlike species composition, which only considers species presence or absence, functional composition incorporates the relative abundances of species within communities[30]. Functional composition also explained more variance in soil food web multifunctionality than did biogeography (20.1%) and functional diversity (1.6%,

Fig. 3d). Further analyses showed a strong positive correlation between the variance components of functional composition and biogeography, suggesting that much of the biogeographic influence on soil food web multifunctionality is mediated through community-level variation in economic traits across locations (Extended Data Table 3, Supplementary results).”

Please see also the Supplementary Results in lines 722-745 of the Supplementary Information.

References

Arif, S. et MacNeil, M.A. (2022) « Predictive models aren’t for causal inference », *Ecology Letters*, 25(8), p. 1741-1745. Disponible sur: https://doi.org/10.1111/ele.14033.

Byrnes, J.E.K. et Dee, L.E. (2025) « Causal Inference With Observational Data and Unobserved Confounding Variables », *Ecology Letters*, 28(1), p. e70023. Disponible sur: https://doi.org/10.1111/ele.70023.

Correia, H.E., Dee, L.E. et Ferraro, P.J. (2025) « Designing causal mediation analyses to quantify intermediary processes in ecology », *Biological Reviews*, n/a(n/a). Disponible sur: https://doi.org/10.1111/brv.70011.

Feng, Y. *et al.* (2022) « Multispecies forest plantations outyield monocultures across a broad range of conditions », *Science*, 376(6595), p. 865-868. Disponible sur: https://doi.org/10.1126/science.abm6363.

Grace, J.B. *et al.* (2012) « Guidelines for a graph-theoretic implementation of structural equation modeling », *Ecosphere*, 3(8), p. art73. Disponible sur: https://doi.org/10.1890/es12-00048.1.

Grace, J.B. et Irvine, K.M. (2020) « Scientist’s guide to developing explanatory statistical models using causal analysis principles », *Ecology*, 101(4), p. e02962. Disponible sur: https://doi.org/10.1002/ecy.2962.

Grace, J.B., Scheiner, S.M. et Schoolmaster Jr, D.R. (2015) « Structural equation modeling: building and evaluating causal models », in G.A. Fox, S. Negrete-Yanlelevich, et V.J. Sosa (éd.) *Ecological statistics: contemporary theory and application*. Oxford, UK: Oxford University Press, p. 168-199.

Hector, A. (1998) « The effect of diversity on productivity: detecting the role of species complementarity », *Oikos*, 82(3), p. 597-599. Disponible sur: https://doi.org/10.2307/3546380.

Hector, A. *et al.* (2011) « BUGS in the Analysis of Biodiversity Experiments: Species Richness and Composition Are of Similar Importance for Grassland Productivity », *PLoS ONE*, 6(3), p. e17434. Disponible sur: https://doi.org/10.1371/journal.pone.0017434.

Nichols, J.D. et Cooch, E.G. (2025) « Predictive models are indeed useful for causal inference », *Ecology*, 106(1), p. e4517. Disponible sur: https://doi.org/10.1002/ecy.4517.

Schulz, T., Saastamoinen, M. et Vanhatalo, J. (2025) « Model-based variance partitioning for statistical ecology », *Ecological Monographs*, 95(1), p. e1646. Disponible sur: https://doi.org/10.1002/ecm.1646.

Schwarz, B. *et al.* (2017) « Warming alters energetic structure and function but not resilience of soil food webs », *Nature Climate Change*, 7(12), p. 895-900. Disponible sur: https://doi.org/10.1038/s41558-017-0002-z.

Yi, H. *et al.* (2025) « Belowground energy fluxes determine tree diversity effects on above- and belowground food webs », *Current Biology*, 35(8), p. 1870-1882.e6. Disponible sur: https://doi.org/10.1016/j.cub.2025.03.034.

RESPONSE LETTER
Manuscript Nature 2025-02-03133A

Dear Dr. [redacted],

Thank you again for your interest in our work and for giving us the opportunity to further revise our manuscript and to clarify a few remaining issues. We are glad to see that Reviewer 1 is happy with the current version and we are grateful to Reviewer 3 for the additional feedback. We appreciate the time that they dedicated to evaluate the revised version of our manuscript.

In this response letter, we address the comments and suggestions of Reviewer 3 point by point, with references to the corresponding changes made in the revised manuscript (highlighted in blue). In particular, we have made final edits to the Abstract to better reflect a more cautious interpretation regarding the relative low importance of tree species mixing *versus* species composition. We have also revised the concluding part of the Results section on tree mixing effects to more clearly outline the contrasting effects of tree species mixing on ecosystem functioning above- and belowground. This point has also been outlined in the Abstract. Finally, we performed some text adjustments to clarify our discussion of the mass ratio hypothesis.

Further, to address your request to enhance the focus on the functioning of the soil food web in the abstract, we have revised it to better emphasize this:

> L. 37-40. "Plants affect terrestrial ecosystem functioning by shaping microenvironments[1], and by providing the primary production that fuels energy flow into food webs[2]. However, how plant community properties affect ecosystem functioning via energy fluxes in food webs has been little studied[3,4], especially for soil food webs that channel most plant-derived energy[2,5]."

Additionally, we have given the manuscript a thorough final read to check for grammar and inconsistencies and have made some minor linguistic changes to the text.

Please note that all changes related to the reviewer's points are now highlighted in red in the revised manuscript, including both the Main Text and Supplementary Information. As requested, we also provide a version with tracked changes to facilitate review. Line numbers cited in our responses refer to the version without tracked changes.

Sincerely yours,

The authors

REVIEWER COMMENTS
Reviewer #1 (Remarks to the Author):

Comment: I have carefully assessed the revised version of this manuscript and feel that the authors have comprehensively addressed my concerns on the initial version, as well as the concerns raised by other reviewers. I would like to commend the authors on this large effort to produce what I think is a very important and interesting study.

Response: We thank the reviewer for his/her careful evaluation of the revised manuscript and for the positive assessment. We appreciate the recognition of our effort and are pleased that the revisions have addressed the concerns raised.

Referee #3 (Remarks to the Author):

Comment: The revised MS is much improved, especially on clarity of the details of what has been done. However, I don't feel the edits to the text fully reflected changes - the abstract was relatively unchanged for example. A further round of minor revision could help the MS better reflect the changes made in review.

Response: We thank the reviewer for this constructive comment. In response, we have revised the Abstract to better reflect the changes made during the review process. We have toned down the previous statement regarding the low importance of tree species mixing relative to species composition and biogeography by removing the original sentence and replacing it with a more cautious formulation that emphasizes the role of tree species composition and biogeography, without explicit reference to species mixing:

> L. 45-47. "Accordingly, tree species composition explained a large portion of the variation in soil food web multifunctionality, comparable to that explained by biogeographic differences among locations"

We also now emphasize the contrasting effects of tree diversity above- and belowground in the Abstract (see our responses below).

Comment: One feature I think could use a little more discussion is the new result (in response to initial reviews) that the trees overyield, despite (or alongside) the soil community results (I think readers would not have expected that). I'm not sure how far you can go in explaining the tree overyielding result in relation to the soil community results but I think it is an intriguing result that suggests some complex and interesting ecology that future studies might want to investigate further. This is a good example of my general comment that the text could do with a final edit to better reflect some of the changes made in response to review: "Overall, our results sharply contrast with most of the studies on species mixing effects, including biodiversity-ecosystem functioning experiments, which typically rely on randomly assembled communities and report positive effects of diversity on ecosystem functions especially for plant diversity". This doesn't seem to consider the overyielding result which I think you characterise as in line with previous results?! This suggests a more nuanced view where mixing can lead to overyielding but alongside the reported below-ground results? This and similar sections need some editing for a bit more nuance especially with regard to changes made during revision. This seems in line with some of the comments by the original review 1 on the original text.

Response: We agree with the reviewer that the tree overyielding result which emerged during the revision process warrants a more nuanced discussion of species mixing effects across aboveground and belowground compartments. We have now revised the relevant section of the results text (L. 199-210) to explicitly integrate this new finding and to reconcile it with the contrasting belowground responses. In this revised text, we emphasize that the observed 17% overyielding in aboveground tree biomass is consistent with previous studies showing complementary effects of tree species mixing and we contrast this positive aboveground response with the weakly negative effects of tree species mixing on soil food web multifunctionality, highlighting that species mixing can simultaneously enhance aboveground tree productivity while altering root allocation and forest microclimate in ways that reduce belowground energy fluxes, thereby leading to divergent responses across ecosystem compartments:

> L. 199-210. "Previous studies have shown that tree species mixing can increase aboveground tree biomass and productivity through resource use complementary[6,7]. In line with this, we observed a 17% overyielding overall in aboveground tree biomass in three-species relative to single-species stands (Extended Data Table 1, see also Fig. 4a and Extended Data Table 2). Meanwhile, tree species mixing has previously been found to reduce the biomass of

absorptive fine roots without affecting overall soil occupation, pointing to morphological root trait adjustments associated with higher resource use efficiency[8]. Consistent with this, the buffering of forest microclimate induced by tree species mixing[9] was associated in our study with lower mean temperatures and reduced thermal energy at ground level. Our results indicate that these changes can collectively lead to a relatively weak decline in soil food web multifunctionality. Together, these findings suggest that tree diversity can exert contrasting effects on ecosystem functioning in different compartments of real-world forests[10], whereby positive responses in aboveground productivity may coincide with, or even contribute to, negative responses belowground."

We also now emphasize the contrasting effects of tree diversity above- and belowground in the Abstract and the Conclusions:

L. 47-50. "In contrast, mixtures of three tree species had weakly negative effects relative to single-species stands, mostly due to shifts in energy channelling from living fine roots to litter and a cooling effect on the forest microclimate. **This occurred despite an overyielding effect in aboveground tree biomass production, suggesting contrasting diversity effects above- and belowground.**"

L. 297-302. "In contrast, tree species mixing (three-species versus single-species stands) and functional diversity had much smaller and, generally, negative effects reflecting a shift in energy channelling from fine roots to litter, alongside a cooling effect on forest microclimate. **This was observed despite tree species mixing having positive effects on aboveground tree biomass production, highlighting the contrasting responses of aboveground and belowground subsystems to higher plant diversity.**"

<u>Comment</u>: Comparing variable importance with variances vs. variance components [I apologize for this rather 'techy' point but I think it is important (and interesting from a philosophy of science perspective) as quantifying the 'importance' of variables is not as simple as we often assume.]

Thanks for clarifying where variances have been used vs. variance components. This is a difficulty of the literature as the terms are often used inconsistently (especially in abbreviated labels in software) when the details of which is used can matter for interpretation. Statistically, it is only in the case of the residual error variance that variance and variance components are the same; for all other terms the variance is composed of the error variance / variance component plus one or more other variance components (the Rails data set example in the Pinheiro and Bates book is one simple example of how variances are made up of their constituent variance components). Whether variables are being compared in terms of the variances or variances components can matter as, all else equal, more complex terms (as reflected in higher numbers of DFs) would be expected to explain more. Simple terms (as quantified statistically in their DFs may explain less, but at a lesser cost and hence the advantage of simplicity (Occam's razor). Variance components are one (fundamental) measure that does not have the same issue of DFs (although no doubt has its own limits etc.). The point here is that, for clarity, I think it is better to use the term 'variance' when referring to variances in the classical mean squares (e.g. the numbers given in the table of variances in ANOVA tables - MS = SS divided by DFs) and variance components for variance components. At a few places in the MS you compare the percent variance explained by different terms - clarify if this is classical variances (e.g. add up all the variances in the ANOVA table and express each as a percentage of that total ~ percent total deviance; ~ R2) or whether the numbers are percentage of the summed variance components (i.e. add up the variance components and express each as a proportion/percent of the summed total - the example I use of this distinction when teaching is the kiwifruit example from Maindonald and Braun Data Analysis and Graphics and the associated DAAG R package) where it is thoroughly explained.

Response: We thank the reviewer for this insightful comment and fully agree the distinction between classical variance and variance components is often blurred in the literature. In our analyses, all statements regarding relative importance of predictor groups are based exclusively on variance components, rather than on classical ANOVA variances or mean squares. Specifically, we performed variance partitioning using a model-based approach in which variance components were estimated for predefined groups of predictors (diversity, composition, and biogeography). The contribution of each group was then expressed as a proportion of the sum of all group-level variance components, including the residual variance component, thereby avoiding confounding effects of differing degrees of freedom among predictor groups.

This procedure is described in the Methods section:

> L. 613-623. "For each model, we performed variance partitioning following a model-based approach using variance components[11] to quantify the proportion of variance in the dependent variable explained by three groups of predictors: diversity (tree species richness or functional dispersion, FDis), composition (tree species composition or CWM trait PC1 and PC2), and biogeography (abiotic conditions PC1 and PC2, and location)[12]… Variance components of each group were standardized by the sum of all group-level variance components, including the residuals."

We agree, however, that this distinction should be made more visible to the readers. To improve clarity, we have therefore added an explicit statement to the legend of Fig. 3:

> L. 1033-1034. "Variance components of each predictor group were standardized by the sum of all group-level variance components, including the residuals."

Further, we now explicitly specify that these results are expressed as 'percentage of the summed variance components', including in the abscissa label text of Fig. 3d and when referring to these results in main text (L. 222, 226, 233-234, and 282-283).

EDITORIAL NOTE: Referee #3 has also provided the following comments on your responses to Referee #2's remaining concerns. We ask that you address these points as well in your revision. Reviewer #3: 'The revised MS is much improved, especially on clarity of the details of what has been done. You might consider some final minor edits to the wording based on the comments below.'

Response: We have addressed the remaining comments below. We have also given the manuscript a thorough final read to check for grammar and inconsistencies and made some minor linguistic changes to the text.

Comment: "Data & Methodology…" The reviewer appreciated the quality of the work. I think there is one-point worth making here in relation to new results that emerged in response to reviews: The mass-ratio hypothesis is described as mechanistic, but I think that is questionable. The progression of this MS illustrates this well. The last step of analysis in response to reviews revealed tree mixing effects – tree mixtures overyield. Before this analysis the tree mixtures appeared a sum of their parts (with the larger parts having the most effect in line with the mass ratio hypothesis). However, the analysis of relative yields based on null expectations derived from performance in monoculture reveals complementary interactions in mixtures, something missed with the previous interpretation. To put it another way, the MR hypothesis cannot discriminate intrinsic effects of species (i.e. trees perform in mixture in the same way as they do in monoculture) from interaction effects (complementarity). Indeed, the MR hypothesis arguably often over exaggerates the effects of dominant species since (without an analysis like the RY method applied here) interaction effects (i.e. including other species) are effectively assigned to the dominant species. (Indeed, is the MR hypothesis even falsifiable – see below).

Regardless, the point here is that the overyielding revealed in the review process missed suggests some reconsideration of the MR hypothesis in interpreting the results – see below.

Response: We thank the reviewer for highlighting how our new results on tree overyielding during the review process informs the interpretation of the mass ratio hypothesis. We do recognize that the analysis of relative yields revealed some complementary effects, most likely due to complementary resource use among co-occurring tree species in mixtures that were not apparent in earlier analyses, and which requires careful consideration.

At the same time, we emphasize that while aboveground tree productivity is an important ecosystem process, it is not a central focus of our study and has already been extensively studied in the literature. The novelty of our manuscript lies in extending biodiversity-ecosystem functioning research to belowground processes by quantifying soil food web multifunctionality using an energy flow framework. From this perspective, the contrasting responses of aboveground productivity and belowground soil food web functioning to tree species mixing emerges as an important result of our study and we agree with the reviewer that we did not highlight this sufficiently well.

A more detailed discussion of the mechanistic and conceptual implications of these findings is provided below, in response to the reviewer's specific final comment below.

Comment: "…use of statistics". The reviewer raises the issues of checks of model fit, assumptions etc. You appear to have done a thorough job of this (and the code will be available for readers to re-run the analysis and make their own judgements). The Bayesian methods also include some 'diagnostics' for stability of the results (R-hat etc.) – I believe these were already mentioned in the MS (but please ensure they are and note any places of concern – in an 'all models are wrong, some models are useful' spirit).

Response: We agree that checking model assumptions is very important. Model convergence and stability diagnostics were performed for all Bayesian analyses, as outlined in the Supplementary Information:

> L. 604-606. "We tested for model convergence by examining trace plots and evaluating the R-hat (*i.e.* the ratio of between-chain variance to within-chain variance) and N_{eff} (*i.e.* the ratio of the effective sample size to the overall number of iterations) statistics."

No indications of problematic convergence or model instability were detected across the fitted models. To improve clarity, we have now explicitly stated in the Main Text (Methods) that all models were checked for their convergence and compliance with statistical assumptions:

> L. 609-611. "Bayesian LMMs were fitted with Markov Chain Monte Carlo (MCMC) methods using non-informative priors for all parameters and each model was checked for their ***convergence*** and compliance with statistical assumptions,…"

In addition, we note that all code will be made publicly available, which will allow readers to re-run the models, inspect diagnostics, and evaluate robustness independently.

Comment: "Probably the most important comment I can make concerns some weaknesses in the comparison between diversity and composition effects…the authors might be over-interpreting the absence of diversity effects". All 3 reviews touched on this general issue. In response the text has been edited including a focus on species mixing rather than species diversity (due to the limited richness gradient). I think this issue has generally been dealt with, but I think the MS would benefit for a final check that the tree overyielding is appropriately taken into account (this result only emerged during review so was not part of the original manuscript). The tree overyielding effects are broadly in line with what is observed in the literature so the text needs to carefully distinguish where mixing effects are weaker (in the soil) vs. where they are typical (at the tree mixture level) so that any general statements could not be

taken to imply a lack of mixing effects overall (i.e. ignoring the tree level effect for the soil effect). For me this difference is one of the most intriguing – that there appear to be typical overyielding effects at the tree level but that these effects are not reflected within the soil community.

Response: We have carefully checked that the tree overyielding results are appropriately taken into account. New text addressing this point has been added to the Main Text:

> L. 200-202. "In line with this, we observed 17% overyielding overall in aboveground tree biomass in three-species relative to single-species stands (Extended Data Table 1, see also Fig. 4a and Extended Data Table 2)."

Comment: "…somewhat strong tone…regarding the role of species richness, which should be interpreted with more caution given the narrow richness gradient used". The MS has been edited to reflect this point. A final careful reading considering some final minor edits may be warranted, especially regarding the typical level of tree overyielding (mixing / richness effects) that emerged during the review process.

Response: We have carefully checked all text on the role of species richness. Further, as explained above, the text on the role of species richness in the Abstract has been revised (lines 45-47) and new text has been added to the Main Text addressing the tree overyielding results and its contrast with belowground responses (lines 199-210).

Comment: Relative yields. This analysis introduced during the review process showed a fairly typical level of overyielding of 17% - see the comments above on ensuring this new result is fully reflected in the final text.

Response: This result is now discussed in lines 200-202. See also our response to comments raised above by Reviewer 3.

Comment L228-239. I have already pointed out some short-comings of the mass ratio hypothesis above: it cannot distinguish complementarity; it can therefore over-exaggerate the effects of dominant species (by attributing interaction effects among two or more species to the dominant alone); that it is arguably vague and therefore it is unclear how to quantify it, how to test it and how falsifiable it is (see below). While it seems likely it is broadly true in general there may be exceptions (e.g. potential impacts of N fixing species that are relatively large even though the species may not be a dominant?) and it ignores interactions (or attributes them to the dominant species). In addition, I am not sure the interpretation here is in line with the original publication. In this MS, the MR hypothesis is interpreted in terms of contrasting species composition vs. functional diversity (one particular type – functional dispersion diversity). In the abstract of Grime 1998 it is not defined in terms of species composition vs. functional composition/diversity: "A 'mass ratio' theory proposes that immediate controls are in proportion to inputs to primary production, are determined to an overwhelming extent by the traits and functional diversity of the dominant plants and are relatively insensitive to the richness of subordinates and transients.". In the main text of Grime 1998 it is described slightly differently (biomass vs. primary production which are not necessarily always strongly correlated): "…the extent to which a plant species affects ecosystem functions is likely to be closely predictable from its contribution to the total plant biomass" From these definitions it seems a direct test would: 1) Quantify individual species contributions to functions. 2) Quantify species biomass / primary productivity (net? Aboveground, belowground or combined?). Analyse 1 and a function of 2.

When the ecosystem function in question is productivity (or surrogates like herbaceous plant biomass), as it often is, then it would seem to risk becoming somewhat circular given a definition of dominant that relates to high relative abundance (biomass explains biomass)? Given this, is your result really support for the efficacy of your functional definitions (since

functional composition is better than species definition) rather than the MR hypothesis? If so, consider adjusting the final text accordingly.

Response: We thank the reviewer for this thoughtful conceptual comment. We agree that the mass ratio hypothesis taken alone may oversimplify community effects, because it can then overemphasize the role of dominant species while underestimating the contributions of other factors such as resource use complementarity. However, we believe that mass ratio and resource use complementarity represent two separate mechanisms of community effects on ecosystem functioning, both implying clear and distinct predictions that can effectively be tested through statistical modelling based on specific metrics related to each mechanism. Though we agree these two mechanisms can operate simultaneously and can therefore be challenging to distinguish, we believe that an integrative statistical framework, as proposed by Díaz *et al.* (2007)[13], that combines mass ratio through community-weighted-mean of traits, niche complementarity through a trait variability metric, and abiotic factors altogether can allow to distinguish between mass ratio and resource use complementarity effects. This is true as long as multicollinearity between functional dispersion and community-weighted-mean of trait spectra is sufficiently low, and we now provide evidence that this was not an issue in our study in a new table in Supplementary Methods (Supplementary Table 16). Finally, with regard to the reviewer's suggestion of a direct test of the mass ratio hypothesis based on species-level relationships between biomass and ecosystem function, we agree that such an approach would be appropriate for functions measured at the level of individual species (*e.g.* productivity). However, this is not feasible for soil food web multifunctionality, which is an emergent property measured at the plot level.

That being said, we agree that some text adjustments were necessary to clarify our discussion of the mass ratio hypothesis and accordingly, we have revised the manuscript as follows:

> L. 142-148. "To gain deeper mechanistic insights, we also adopted a functional (trait-based) approach[13–15] to assess the effects of both functional diversity and functional composition of tree communities, alongside biogeographic differences among locations. This enabled us to ***contrast patterns consistent with the 'niche complementarity'*** hypothesis, which states that functionally more diverse communities promote ecosystem functioning through ***greater resource use*** complementarity[13], with ***patterns consistent with*** the 'mass-ratio' hypothesis, which states that ecosystem functioning is primarily driven by the mean attributes (trait values) of species weighted by their relative contribution to community biomass[16]."
>
> L. 231-239. "Soil food web multifunctionality ***was even more strongly associated with the functional composition of tree communities (quantified as community-weighted mean values of traits that are related to resource economics)*** than with species composition within geographic locations alone (36.7% *versus* 22.3% of summed variance components, Fig. 3b,d). This ***pattern is consistent with expectations derived from the 'mass ratio' hypothesis[16], whereby ecosystem functioning is primarily determined by the traits of the most abundant species. Although resource use complementarity may also contribute to ecosystem functioning, our analyses explicitly evaluated mass ratio effects through community-weighted mean traits alongside resource use complementarity effects through trait dispersion, allowing these mechanisms to be considered as distinct, though potentially co-occurring, drivers[13]."***

Please note that we renamed the 'functional diversity' hypothesis (which was a little too vague) as the 'niche complementarity' hypothesis.

Comment I hope these final points are useful.

Response: They were indeed! We thank you very much for your constructive comments that helped us further improve the manuscript.

References

1. Jones, C. G., Lawton, J. H. & Shachak, M. Positive and negative effects of organisms as physical ecosystem engineers. *Ecology* **78**, 1946–1957 (1997).
2. Cebrian, J. Patterns in the fate of production in plant communities. *Am. Nat.* **154**, 449–468 (1999).
3. Cardinale, B. J. *et al.* The functional role of producer diversity in ecosystems. *Am. J. Bot.* **98**, 572–592 (2011).
4. Eisenhauer, N. *et al.* Chapter One - A multitrophic perspective on biodiversity–ecosystem functioning research. in *Advances in Ecological Research* (eds Eisenhauer, N., Bohan, D. A. & Dumbrell, A. J.) vol. 61 1–54 (Academic Press, 2019).
5. Wardle, D. A. *et al.* Ecological linkages between aboveground and belowground biota. *Science* **304**, 1629–1633 (2004).
6. Jucker, T., Bouriaud, O., Avacaritei, D. & Coomes, D. A. Stabilizing effects of diversity on aboveground wood production in forest ecosystems: linking patterns and processes. *Ecol. Lett.* **17**, 1560–1569 (2014).
7. Feng, Y. *et al.* Multispecies forest plantations outyield monocultures across a broad range of conditions. *Science* **376**, 865–868 (2022).
8. Wambsganss, J., Beyer, F., Freschet, G. T., Scherer-Lorenzen, M. & Bauhus, J. Tree species mixing reduces biomass but increases length of absorptive fine roots in European forests. *J. Ecol.* **109**, 2678–2691 (2021).
9. Schnabel, F. *et al.* Tree Diversity Increases Forest Temperature Buffering via Enhancing Canopy Density and Structural Diversity. *Ecol. Lett.* **28**, e70096 (2025).
10. van der Plas, F. Biodiversity and ecosystem functioning in naturally assembled communities. *Biol. Rev.* **94**, 1220–1245 (2019).
11. Gelman, A. *et al. Bayesian Data Analysis*. (CRC Press, 2013).
12. Schulz, T., Saastamoinen, M. & Vanhatalo, J. Model-based variance partitioning for statistical ecology. *Ecol. Monogr.* **95**, e1646 (2025).
13. Díaz, S. *et al.* Incorporating plant functional diversity effects in ecosystem service assessments. *Proc. Natl. Acad. Sci.* **104**, 20684–20689 (2007).
14. Reiss, J., Bridle, J. R., Montoya, J. M. & Woodward, G. Emerging horizons in biodiversity and ecosystem functioning research. *Trends Ecol. Evol.* **24**, 505–514 (2009).
15. Chacón-Labella, J. *et al.* How to improve scaling from traits to ecosystem processes. *Trends Ecol. Evol.* **38**, 228–237 (2023).
16. Grime, J. P. Benefits of plant diversity to ecosystems: immediate, filter and founder effects. *J. Ecol.* **86**, 902–910 (1998).

RESPONSE LETTER
Manuscript Nature 2025-02-03133B

Dear Dr. [redacted],

Thank you again for your interest in our work. Please find below our response to the one final point raised by Reviewer 3.

Sincerely yours,

The authors

REVIEWER COMMENTS

Referee #3 (Remarks to the Author):

Thank you for your revised version – I have only one final small suggestion:

Comment: "This enabled us to contrast patterns consistent with the 'niche complementarity' hypothesis, which states that functionally more diverse communities promote ecosystem functioning through greater resource use complementarity"

It is true that early complementarity work focused on resource use, and there is evidence for this, but in some cases the complementarity comes via other mechanisms, including natural enemies (reduced pests and diseases in mixtures), facilitation (N fixation, hydraulic lift, nurse plant effects etc.) so perhaps consider a final minor tweak to acknowledge this wide range of possible niche and complementarity effects (something like): "…promote ecosystem functioning through complementarity species interactions, with patterns…"

Response: We thank the reviewer for this final suggestion. We agree that complementarity effects can extend beyond resource use partitioning and also include abiotic facilitation and biotic feedbacks processes. In response, we now refer to 'complementarity effects' instead of 'resource use complementarity' to be more inclusive of these multiple processes. Accordingly, we have revised the sentence raised as follow:

> L. 144-146. "This enabled us to contrast patterns consistent with the 'niche complementarity' hypothesis, which states that functionally more diverse communities promote ecosystem functioning through greater ***complementarity effects***".

Similar text adjustments have been performed lines 236 and 237.
